# Understanding the potential bias of variance components estimators when using genomic models

**DOI:** 10.1186/s12711-018-0411-0

**Published:** 2018-08-06

**Authors:** Beatriz C. D. Cuyabano, A. Christian Sørensen, Peter Sørensen

**Affiliations:** Center for Quantitative Genetics and Genomics, Department of Molecular Biology and Genetics, Aarhus University, Blichers Allé 20, Postboks 50, 8830 Tjele, Denmark

## Abstract

**Background:**

Genomic models that link phenotypes to dense genotype information are increasingly being used for infering variance parameters in genetics studies. The variance parameters of these models can be inferred using restricted maximum likelihood, which produces consistent, asymptotically normal estimates of variance components under the true model. These properties are not guaranteed to hold when the covariance structure of the data specified by the genomic model differs substantially from the covariance structure specified by the true model, and in this case, the likelihood of the model is said to be misspecified. If the covariance structure specified by the genomic model provides a poor description of that specified by the true model, the likelihood misspecification may lead to incorrect inferences.

**Results:**

This work provides a theoretical analysis of the genomic models based on splitting the misspecified likelihood equations into components, which isolate those that contribute to incorrect inferences, providing an informative measure, defined as $\varvec{\kappa }$, to compare the covariance structure of the data specified by the genomic and the true models. This comparison of the covariance structures allows us to determine whether or not bias in the variance components estimates is expected to occur.

**Conclusions:**

The theory presented can be used to provide an explanation for the success of a number of recently reported approaches that are suggested to remove sources of bias of heritability estimates. Furthermore, however complex is the quantification of this bias, we can determine that, in genomic models that consider a single genomic component to estimate heritability (assuming SNP effects are all *i.i.d.*), the bias of the estimator tends to be downward, when it exists.

**Electronic supplementary material:**

The online version of this article (10.1186/s12711-018-0411-0) contains supplementary material, which is available to authorized users.

## Background

Genomic models that incorporate dense genotype information are increasingly being used and studied to infer variance parameters [1–4]. We define a genomic model as any linear mixed model (LMM) that links a phenotype to multiple genotypes without knowledge of those that are associated with the phenotype. We refer to a general set of genotypes as single nucleotide polymorphisms (SNPs) and to the set of genotypes associated with the phenotype as quantitative trait loci (QTL). The variance parameters of the LMM can be inferred using restricted maximum likelihood (REML) [5], which produces consistent, asymptotically normal estimators of variance components, even if normality does not hold and the number of QTL increases dramatically, tending to infinity [6]. These asymptotic properties of the REML estimators are not guaranteed to hold when the likelihood of the genomic model used for inference differs substantially from the likelihood of the true model that conceptually generated the data. In such a situation, the likelihood is said to be misspecified. In a Gaussian setup, given the fixed effects, this will be the case when the covariance structures of the data specified by the genomic and the true models differ.

**Fig. 1:**
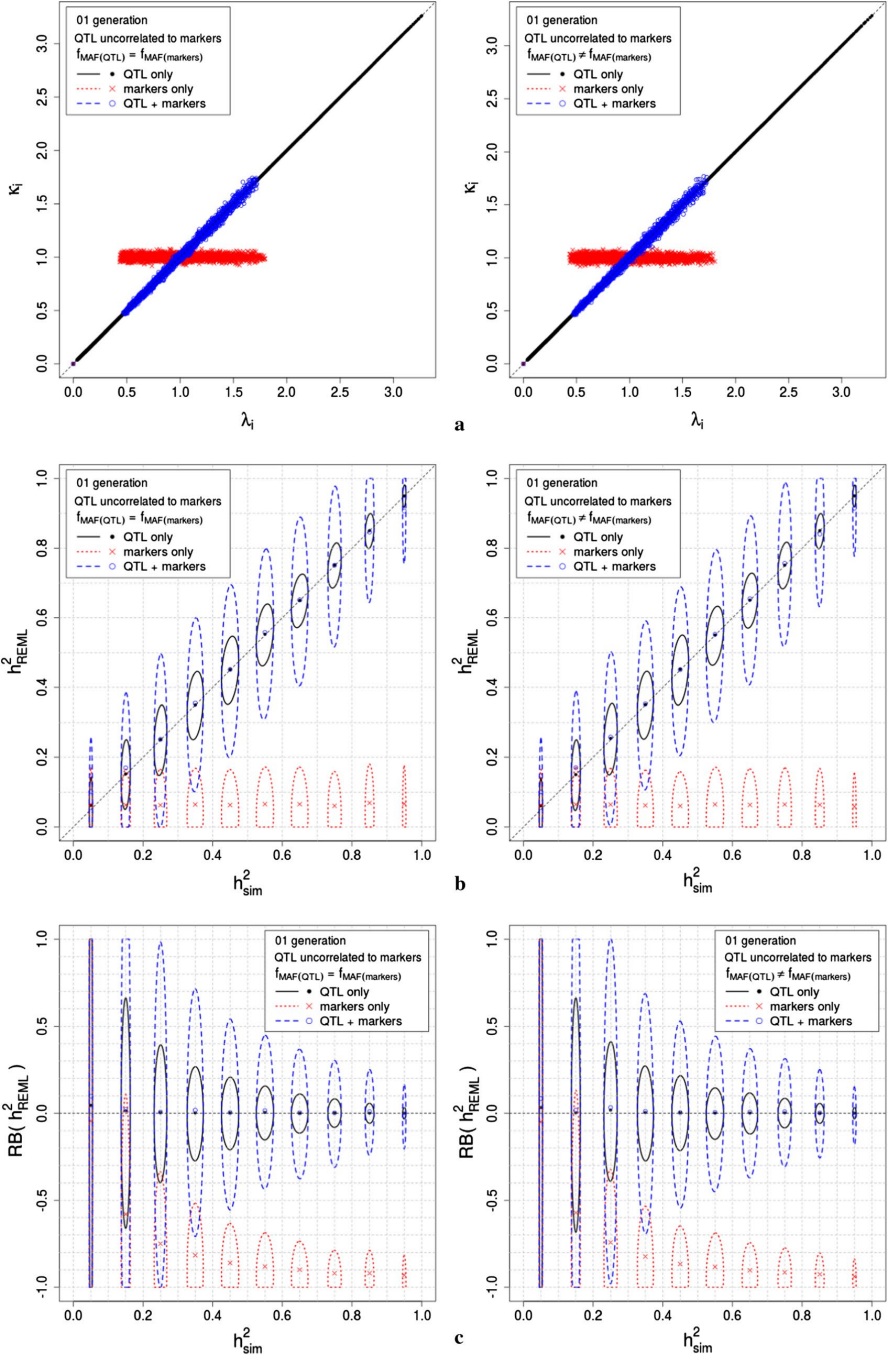
Simulation results for scenarios 1 and 2, consisting of one generation of completely unrelated individuals, with QTL and markers in complete linkage equilibrium (LE), for both $\hbox {f}_{\mathrm{MAF}_{_{\mathrm{QTL}}}} = \hbox {f}_{\mathrm{MAF}_{_{\mathrm{markers}}}}$ and $\hbox {f}_{\mathrm{MAF}_{_{\mathrm{QTL}}}} \ne \hbox {f}_{\mathrm{MAF}_{_{\mathrm{markers}}}}$. Simulations were performed with 3000 QTL generating the phenotypes, replicated 500 times. **a** shows the relationship between $\lambda _{\mathrm{i}}$ and $\kappa _{\mathrm{i}}$, for the true model (QTL only) and for both genomic models evaluated (QTL plus markers and markers only); **b** presents the confidence ellipses for the simulated heritabilities ($\hbox {h}_{\mathrm{sim}}^{2} = \gamma _{_{\mathrm{sim}}}/(1+\gamma _{_{\mathrm{sim}}})$), with a simulation parameter $\hbox {h}^{2}=0.05,0.15,\ldots ,0.95$, and the heritabilities estimated using REML ($\hbox {h}_{\mathrm{REML}}^{2} = \gamma _{_{\mathrm{REML}}}/(1+\gamma _{_{\mathrm{REML}}})$), for the true model (QTL only) and for both genomic models evaluated (QTL plus markers and markers only); **c** presents the confidence ellipses for the simulated heritabilities ($\hbox {h}_{\mathrm{sim}}^{2} = \gamma _{_{sim}}/(1+\gamma _{_{\mathrm{sim}}})$), with a simulation parameter $\hbox {h}^{2}=0.05,0.15,\ldots ,0.95$, and the relative bias of $\hbox {h}_{\mathrm{REML}}^{2}$ ($\hbox {RB}(\hbox {h}_{\mathrm{REML}}^{2}) = (\hbox {h}_{\mathrm{REML}}^{2}-\hbox {h}_{\mathrm{sim}}^{2})/\hbox {h}_{\mathrm{sim}}^{2}$)

**Fig. 2:**
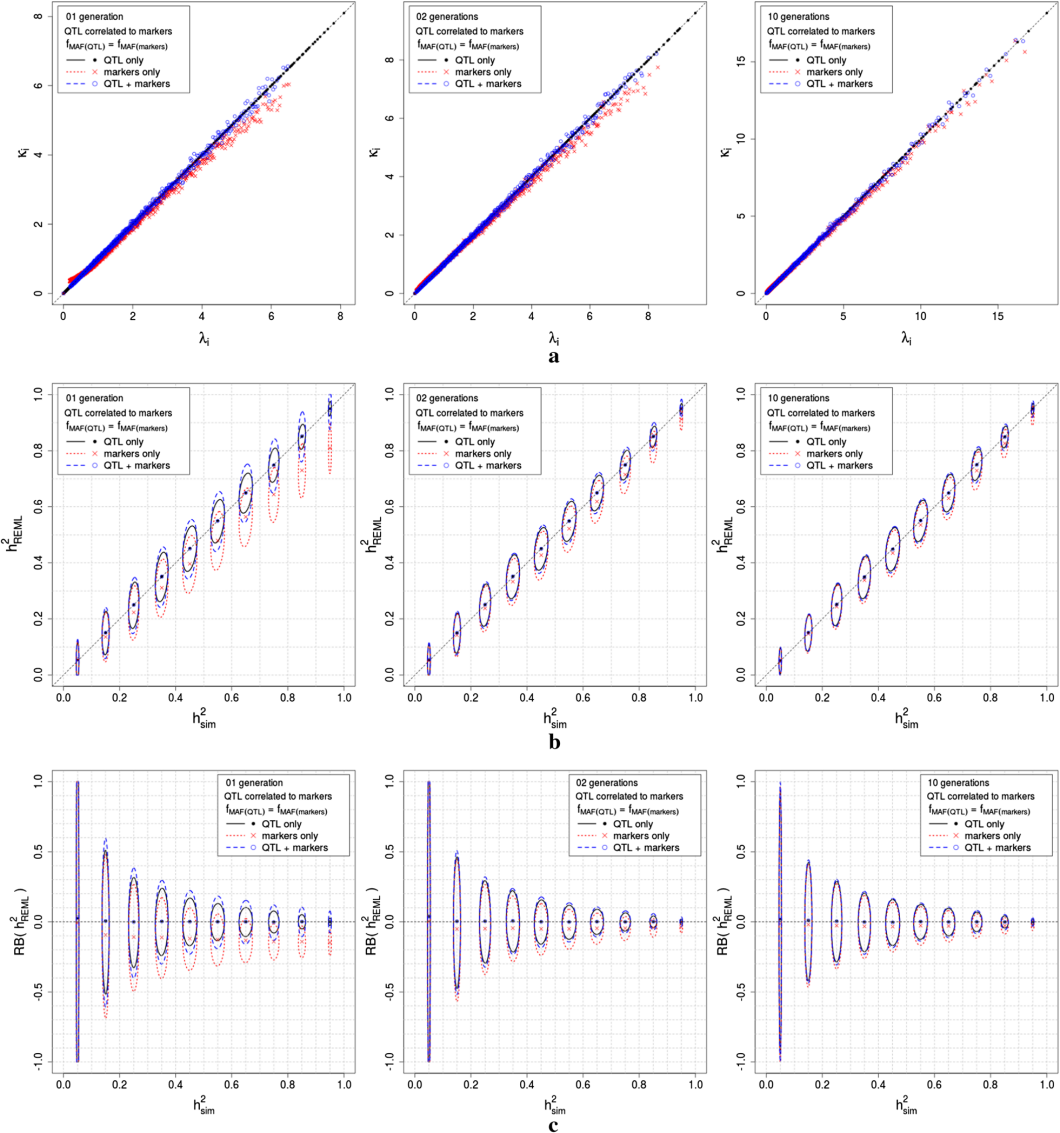
Simulation results for scenarios 3, 4 and 5, consisting of one generation of completely unrelated individuals and two and 10 generations of related individuals, with QTL and markers in LD, for $\hbox {f}_{\mathrm{MAF}_{_{\mathrm{QTL}}}} = \hbox {f}_{\mathrm{MAF}_{_{\mathrm{markers}}}}$. Simulations were performed with 3000 QTL generating the phenotypes, replicated 500 times. **a** shows the relationship between $\lambda _{\mathrm{i}}$ and $\kappa _{\mathrm{i}}$, for the true model (QTL only) and for both genomic models evaluated (QTL plus markers and markers only); **b** presents the confidence ellipses for the simulated heritabilities ($\hbox {h}_{\mathrm{sim}}^{2} = \gamma _{_{\mathrm{sim}}}/(1+\gamma _{_{\mathrm{sim}}})$), with a simulation parameter $\hbox {h}^{2}=0.05,0.15,\ldots ,0.95$, and the heritabilities estimated using REML ($\hbox {h}_{\mathrm{REML}}^{2} = \gamma _{_{\mathrm{REML}}}/(1+\gamma _{_{\mathrm{REML}}})$), for the true model (QTL only) and for both genomic models evaluated (QTL plus markers and markers only); **c** presents the confidence ellipses for the simulated heritabilities ($\hbox {h}_{\mathrm{sim}}^{2} = \gamma _{_{\mathrm{sim}}}/(1+\gamma _{_{\mathrm{sim}}})$), with a simulation parameter $\hbox {h}^{2}=0.05,0.15,\ldots ,0.95$, and the relative bias of $\hbox {h}_{\mathrm{REML}}^{2}$ ($\hbox {RB}(\hbox {h}_{\mathrm{REML}}^{2}) = (\hbox {h}_{\mathrm{REML}}^{2}-\hbox {h}_{\mathrm{sim}}^{2})/\hbox {h}_{\mathrm{sim}}^{2}$)

**Fig. 3:**
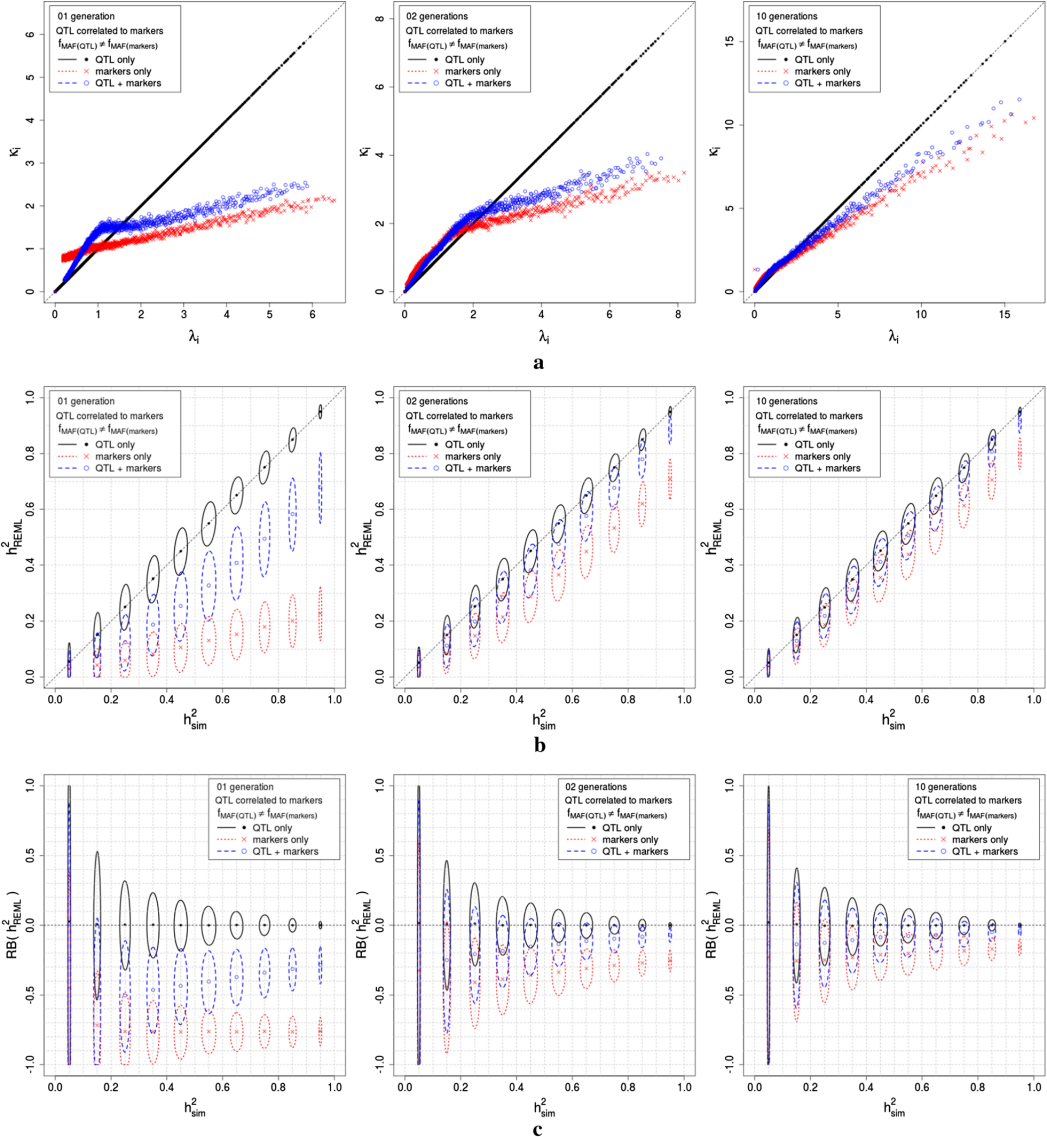
Simulation results for scenarios 6, 7 and 8, consisting of one generation of completely unrelated individuals and two and 10 generations of related individuals, with QTL and markers in LD, for $\hbox {f}_{\mathrm{MAF}_{_{\mathrm{QTL}}}} \ne \hbox {f}_{\mathrm{MAF}_{_{\mathrm{markers}}}}$. Simulations were performed with 3000 QTL generating the phenotypes, replicated 500 times. Panel (a) shows the relationship between $\lambda _{\mathrm{i}}$ and $\kappa _{\mathrm{i}}$, for the true model (QTL only) and for both genomic models evaluated (QTL plus markers and markers only); panel (b) presents the confidence ellipses for the simulated heritabilities ($\hbox {h}_{\mathrm{sim}}^{2} = \gamma _{_{\mathrm{sim}}}/(1+\gamma _{_{\mathrm{sim}}})$), with a simulation parameter $\hbox {h}^{2}=0.05,0.15,\ldots ,0.95$, and the heritabilities estimated using REML ($\hbox {h}_{\mathrm{REML}}^{2} = \gamma _{_{\mathrm{REML}}}/(1+\gamma _{_{\mathrm{REML}}})$), for the true model (QTL only) and for both genomic models evaluated (QTL plus markers and markers only); panel (c) presents the confidence ellipses for the simulated heritabilities ($\hbox {h}_{\mathrm{sim}}^{2} = \gamma _{_{\mathrm{sim}}}/(1+\gamma _{_{\mathrm{sim}}})$), with a simulation parameter $\hbox {h}^{2}=0.05,0.15,\ldots ,0.95$, and the relative bias of $\hbox {h}_{\mathrm{REML}}^{2}$ ($\hbox {RB}(\hbox {h}_{\mathrm{REML}}^{2}) = (\hbox {h}_{\mathrm{REML}}^{2}-\hbox {h}_{\mathrm{sim}}^{2})/\hbox {h}_{\mathrm{sim}}^{2}$)

The correct covariance structure (referred to in our work as $\mathbf{G }_{\mathrm{Q}}$) requires knowledge of the QTL. Since these are typically unknown, in practice, the genomic model makes use of the available SNP genotypes instead in order to compute a covariance structure (referred to in our work as $\mathbf{G }$), leading to misspecification of the likelihood. The patterns of realized relationships at different sets of loci vary across the genome [7]. Because of this, $\mathbf{G }$ may provide a poor description of $\mathbf{G }_{\mathrm{Q}}$, and the likelihood misspecification may lead to biased estimators of variance parameters.

REML was first implemented with a genomic model in [1], where the focus of inference was the proportion of the variance of a quantitative trait explained by the LMM, including all genotyped SNPs simultaneously. In more recent years, concerns have been raised about the quality of the inferred variance parameters when genomic models are used without directly addressing the problem of likelihood misspecification. Speed et al. [4] argued that uneven linkage disequilibrium (LD) between SNPs can lead to upward or downward bias of variance parameters estimators. The consequences of using $\mathbf{G }$ instead of $\mathbf{G }_{\mathrm{Q}}$ on the likelihood were also investigated by [8]. These authors used the singular value decomposition of $\mathbf{G }$ and expressed the likelihood function of the genomic model as a function of these decompositions, concluding that the likelihood-based estimators are unreliable because they are sensitive to small perturbations on the eigen-values. This work generated back-and-forth discussions [9, 10].

The problem of misspecification of the likelihood of the genomic model was first raised by [11] and was studied using simulation by [12]. However, in [12] the authors addressed the problem by redefining the variance parameters according to the genomic models. In our work, we compare the variance parameters estimators to the parameters defined by the true model, as previously studied by [13]. The assumptions posed by [13] on the true model, however, differ substantially from those posed by our study, which can lead to different conclusions. Jiang et al. [13] assumed that the number of QTL associated with a phenotype are large enough to be considered infinite, an assumption which we consider rather unrealistic, and therefore our study assumes that the number of QTL is finite, although possibly very large.

This work provides a theoretical analysis based on splitting the likelihood equations into components, isolating those that contribute to incorrect inferences. We describe a true model that associates a phenotype with QTL, and we use its likelihood as a basis for comparison with the likelihood of the genomic models. This theory was used to understand the potential bias of REML estimators of variance components under different scenarios, each with different assumptions on the true model, that are of interest in quantitative genetics studies.

**Table 1:** Relationship between $\gamma$ and the REML estimators obtained from data with QTL plus markers ($\hat{\gamma }_{\mathrm{QM}}$) and markers only ($\hat{\gamma }_{\mathrm{M}}$), assuming $\hbox {q}$ fixed and finite, $\hbox {m}$ very large, $\hbox {m} > \hbox {q}$, and $\hbox {q}+\hbox {m}>>> \hbox {n}$

Scenario	Population	MAF	QTL/markers	${\lim _{\mathrm{n}\rightarrow \infty }}$	
				${\mathbb {E}(\hat{\gamma }_{\mathrm{QM}})}$	${\mathbb {E}(\hat{\gamma }_{\mathrm{M}})}$
1	1 generation$^{*}$	$\hbox {f}_{\mathrm{MAF}_{_{\mathrm{QTL}}}}=\hbox {f}_{\mathrm{MAF}_{_{\mathrm{makers}}}}$	Complete LE	$\gamma$	0
2	1 generation$^{*}$	$\hbox {f}_{\mathrm{MAF}_{_{\mathrm{QTL}}}}\ne \hbox {f}_{\mathrm{MAF}_{\mathrm{makers}}}$	Complete LE	$\gamma$	0
3	1 generation$^{*}$	$\hbox {f}_{\mathrm{MAF}_{_{\mathrm{QTL}}}}=\hbox {f}_{\mathrm{MAF}_{\mathrm{makers}}}$	LD	$\gamma$	$<<< \mathbb {E}(\hat{\gamma }_{\mathrm{QM}})$
4	2 generations	$\hbox {f}_{\mathrm{MAF}_{_{\mathrm{QTL}}}}=\hbox {f}_{\mathrm{MAF}_{\mathrm{makers}}}$	LD	$\gamma$	$<< \mathbb {E}(\hat{\gamma }_{\mathrm{QM}})$
5	10 generations	$\hbox {f}_{\mathrm{MAF}_{_{\mathrm{QTL}}}}=\hbox {f}_{\mathrm{MAF}_{\mathrm{makers}}}$	LD	$\gamma$	$< \mathbb {E}(\hat{\gamma }_{\mathrm{QM}})^{\dagger }$
6	1 generation$^{*}$	$\hbox {f}_{\mathrm{MAF}_{_{\mathrm{QTL}}}}\ne \hbox {f}_{\mathrm{MAF}_{\mathrm{makers}}}$	LD	$<<< \gamma$	$<<< \mathbb {E}(\hat{\gamma }_{\mathrm{QM}})$
7	2 generations	$\hbox {f}_{\mathrm{MAF}_{_{\mathrm{QTL}}}}\ne \hbox {f}_{\mathrm{MAF}_{\mathrm{makers}}}$	LD	$<< \gamma$	$<< \mathbb {E}(\hat{\gamma }_{\mathrm{QM}})$
8	10 generations	$\hbox {f}_{\mathrm{MAF}_{_{\mathrm{QTL}}}}\ne \hbox {f}_{\mathrm{MAF}_{\mathrm{makers}}}$	LD	$< \gamma ^{\dagger }$	$< \mathbb {E}(\hat{\gamma }_{\mathrm{QM}})^{\dagger }$

*Completely unrelated individuals

$^{\dagger }\lim _{\mathrm{g}\uparrow }\mathrm{h}^{2}_{\mathrm{M}}=\lim _{\mathrm{g}\uparrow }\mathrm{h}^{2}_{\mathrm{QM}}=\mathrm{h}^{2}$ for a large number $\hbox {g}$ of generations (strongly related individuals)

## Methods

### True and genomic models

We start this section by describing a general model that links phenotypes of a complex trait to genotype data:

1 $$\begin{aligned} \mathbf{y }= & \mathbf{1 }_{\mathrm{n}}\mu + \mathbf{W }\mathbf{b } + \varvec{\varepsilon }, \end{aligned}$$

where $\mu$ is the overall mean, $\mathbf{W }$ is a $\hbox {n} \times \hbox {s}$ standardized SNP genotypes matrix (with $\hbox {W}_{\mathrm{ij}} = (\hbox {Z}_{\mathrm{ij}} - 2\theta _{\mathrm{j}})/\sqrt{2\theta _{\mathrm{j}}(1 - \theta _{\mathrm{j}})}$, $\mathbb {E}(\hbox {W}_{\mathrm{ij}}) = 0$, and $\mathbb {V}\hbox {ar}(\hbox {W}_{\mathrm{ij}}) = 1$; $\hbox {Z}_{\mathrm{ij}} \in \left\{ 0,1,2\right\}$ is the count of the minor allele at the $\hbox {j-th}$ SNP with minor allele frequency (MAF) $\theta _{\mathrm{j}}$, of the $\hbox {i-th}$ individual, for all $\hbox {i}=1,\ldots ,\hbox {n}$ and $\hbox {j}=1,\ldots ,\hbox {s}$), $\mathbf{b } \sim \hbox {N}(\mathbf{0 },\mathbf{I }_{\mathrm{s}}\sigma ^{2}_{\mathrm{b}})$ is an $\hbox {s}\times 1$ vector of random SNP effects and $\varvec{\varepsilon } \sim \hbox {N}(\mathbf{I }_{\mathrm{n}}\sigma ^{2}_{\varepsilon _{Q}})$ is an $\hbox {n} \times 1$ vector of the model’s residuals. In the true model, $\mathbf{W } \doteq \mathbf{W }_{\mathrm{Q}}$ is a $\hbox {n} \times \hbox {q}$ ($\hbox {q} \le \hbox {s}$) matrix containing only the QTL, and all the elements and parameters that describe the model are sub-indexed with $\hbox {Q}$. In the genomic model, $\mathbf{W }$ contains $\hbox {s} = \hbox {m} + \hbox {q}$ SNPs, with $\hbox {m}=0,\ldots ,\hbox {s}$ the number of markers (non causative mutations). When $\hbox {m} = 0$, we are in fact in the case of the true model, and when $\hbox {m} = \hbox {s}$ the genomic model contains no QTL in the SNP data.

### Variance components and REML estimation

The covariance structure specified by the true and genomic models are $\mathbf{G }_{\mathrm{Q}} = \hbox {q}^{-1}\mathbf{W }_{\mathrm{Q}}\mathbf{W }_{\mathrm{Q}}'$ and $\mathbf{G } = \hbox {s}^{-1}\mathbf{W }\mathbf{W }'$, respectively, which also define the relationships between individuals at the genotype level [14]. Let $\sigma ^{2}_{\mathrm{T}} = \hbox {s}\sigma ^{2}_{\mathrm{b}}$ (total variance due to the genotypes), and $\gamma = \sigma ^{2}_{\mathrm{T}}/\sigma ^{2}_{\varepsilon }$, and define the matrix $\mathbf{V }(\gamma ) \doteq \mathbf{V } = \gamma \mathbf{G } + \mathbf{I }_{\mathrm{n}}$, we then have that $\mathbb {V}\hbox {ar}(\mathbf{y } \,\, \mid \,\, \mathbf{W }) = \mathbb {V}\hbox {ar}(\mathbf{W } \mathbf{b }) + \mathbb {V}\hbox {ar}(\varvec{\varepsilon }) = \sigma ^{2}_{\mathrm{b}}\mathbf{W }\mathbf{W }' + \sigma ^{2}_{\varepsilon }\mathbf{I }_{\mathrm{n}} = \sigma ^{2}_{\mathrm{T}}\mathbf{G } + \sigma ^{2}_{\varepsilon }\mathbf{I }_{\mathrm{n}} = \sigma ^{2}_{\varepsilon }\mathbf{V }$.

In genetics studies, the interest lies in estimating the narrow-sense heritability, *i. e.* the proportion of phenotypic variance explained by the genotypes. Under the true model, the heritability is defined as $\hbox {h}^{2} = \sigma ^{2}_{\mathrm{T}_{\mathrm{Q}}}/(\sigma ^{2}_{\mathrm{T}_{\mathrm{Q}}} + \sigma ^{2}_ {\varepsilon _{\mathrm{Q}}}) = \gamma _{_{\mathrm{Q}}}/(\gamma _{_{\mathrm{Q}}} + 1)$. Analogously, under the genomic model we have $\hbox {h}^{2}_{\mathrm{gen}} = \gamma /(\gamma + 1)$. When we fit the true model (QTL only), $\lim _{\mathrm{n}\rightarrow \infty }\hat{\gamma }_{_{\mathrm{Q}}} = \gamma _{_{\mathrm{Q}}}$ and consequently $\lim _{\mathrm{n}\rightarrow \infty }\hat{\mathrm{h}}^{2} = \lim _{\mathrm{n}\rightarrow \infty }\hat{\gamma }_{_{\mathrm{Q}}}/(\hat{\gamma }_{_{\mathrm{Q}}} + 1) = \hbox {h}^{2}$, for any number of QTL [5], even if normality does not hold, as demonstrated by [6] using large-sample theory. We also used large-sample theory to evaluate the likelihood of the genomic model, which is misspecified to the likelihood of the model that conceptually generated the phenotypes. Intuitively, if when we fit the genomic model, we obtain $\lim _{\mathrm{n}\rightarrow \infty }\hat{\gamma } = \gamma _{_{\mathrm{Q}}}$, then $\lim _{\mathrm{n}\rightarrow \infty }\hat{\hbox {h}}^{2}_{\mathrm{gen}} = \lim _{\mathrm{n}\rightarrow \infty }\hat{\gamma }/(\hat{\gamma } + 1) = \hbox {h}^{2}$. This means that if $\lim _{\mathrm{n}\rightarrow \infty }\mathbb {E}(\hat{\gamma })=\gamma _{_{\mathrm{Q}}}$, then $\lim _{\mathrm{n}\rightarrow \infty }\mathbb {E}(\hat{\hbox {h}}^{2}_{\mathrm{gen}})=\hbox {h}^{2}$. Therefore, because REML will yield the estimator $\hat{\gamma }$, we focus our analysis on $\mathbb {E}(\hat{\gamma })$.

Define $\mathbf{P }(\gamma ) \doteq \mathbf{P } = \mathbf{V }^{-1} - \mathbf{V }^{-1}\mathbf{1 }_{\mathrm{n}}\left( \mathbf{1 }_{\mathrm{n}}'\mathbf{V }^{-1}\mathbf{1 }_{\mathrm{n}}\right) ^{-1}\mathbf{1 }_{\mathrm{n}}'\mathbf{V }^{-1}$. The REML log-likelihood of the genomic model is [15]:

2 $$\begin{aligned} \ell \left( \gamma ,\sigma ^{2}_{\varepsilon } \mid \mathbf{W }\right)\propto & -\log \left( \sigma ^{\mathrm{2n}}_{\varepsilon }\mid \mathbf{V } \mid \right) - \log \left( \sigma ^{-2}_{\varepsilon }\mid \mathbf{1 }_{\mathrm{n}}'\mathbf{V }^{-1}\mathbf{1 }_{\mathrm{n}} \mid \right) -\sigma ^{-2}_{\varepsilon }\mathbf{y }'\mathbf{P }\mathbf{y }, \end{aligned}$$

and by equating its gradient to zero at the point of maximum, $\hat{\gamma }$ is the root of the REML equation [16]:

3 $$\begin{aligned} \frac{\mathbf{y }'\mathbf{P }\mathbf{G }\mathbf{P }\mathbf{y }}{\hbox {tr}\left( \mathbf{P }\mathbf{G }\right) } - \frac{\mathbf{y }'\mathbf{P }^{2}\mathbf{y }}{\hbox {tr}\left( \mathbf{P }\right) } =\, 0. \end{aligned}$$

Using the eigen-decomposition $\mathbf{G } = \mathbf{U }\varvec{\Lambda }\mathbf{U }'$, the REML Eq. (3) can be written as a function of $\gamma$ (see “Appendix 1”):

4 $$\begin{aligned} g(\gamma )= & \sum _{\mathrm{i}=1}^{\mathrm{n}-1}\sum _{\mathrm{j}=1}^{\mathrm{n}-1}\frac{(\mathbf{y }'\mathbf{U }_{\mathrm{i}})^{2}}{(1 + \gamma \lambda _{\mathrm{i}})^{2}}\left( \frac{\lambda _{\mathrm{i}} - \lambda _{\mathrm{j}}}{1 + \gamma \lambda _{\mathrm{j}}}\right) + \hbox {n}\bar{\mathrm{y}}^{2}\sum _{\mathrm{j}=1}^{\mathrm{n}-1}\frac{\lambda _{\mathrm{j}}}{1 + \gamma \lambda _{\mathrm{j}}}, \end{aligned}$$

such that $\hat{\gamma }$ is the root of $\hbox {g}(\gamma )$. Now, since $(\mathbf{y }'\mathbf{U }_{\mathrm{i}})^{2} \longrightarrow \mu ^{2}(\mathbf{1 }_{\mathrm{n}}'\mathbf{U }_{\mathrm{i}})^{2} + (\mathbf{b }_{\mathrm{Q}}'\mathbf{W }_{\mathrm{Q}}'\mathbf{U }_{\mathrm{i}})^{2}$ for $\hbox {n}$ sufficiently large (see “Appendix 2”), with $(\mathbf{b }_{\mathrm{Q}}'\mathbf{W }_{\mathrm{Q}}'\mathbf{U }_{\mathrm{i}})^{2} \propto \sum _{\mathrm{k}=1}^{\mathrm{n}}(\mathbf{U }_ {\mathrm{i}}'\mathbf{U }_{\mathrm{Qk}})^{2}\lambda _{\mathrm{Qk}}$, we can write the non-observable REML function at the root as the following (see “Appendix 3”):

5 $$\begin{aligned} \sum _{\mathrm{i}=1}^{\mathrm{n}-1}\sum _{\mathrm{j}=1}^{\mathrm{n}-1}\frac{\sum _{\mathrm{k}=1}^{\mathrm{n}}(\mathbf{U }_ {\mathrm{i}}'\mathbf{U }_{\mathrm{Qk}})^{2}\lambda _{\mathrm{Qk}}}{(1 + \hat{\gamma }\lambda _{\mathrm{i}})^{2}}\left( \frac{\lambda _{\mathrm{i}} - \lambda _{\mathrm{j}}}{1 + \hat{\gamma }\lambda _{\mathrm{j}}}\right) =\, 0. \end{aligned}$$

We refer to Eq. (5) as non-observable because it is written as a function of $\mathbf{U }_{\mathrm{Q}}$ and $\varvec{\Lambda }_{\mathrm{Q}}$, which cannot be observed directly when only phenotype and genomic data are available, and we have no knowledge about the QTL. The use of such function is purely theoretical as an aid to obtaining deeper understanding of REML mechanisms. In practical implementations, we find the root of Eq. (4) to obtain $\hat{\gamma }$.

### Genomic models for scenarios of interest in quantitative genetics

We evaluated genomic models for SNP data consisting of QTL and markers that can be either uncorrelated or correlated, considering two configurations: (i) QTL plus markers and (ii) markers only. The covariance structure specified by these configurations, as well as their eigen-decomposition are denoted respectively by $\mathbf{G }_{\mathrm{QM}} = \mathbf{U }_{\mathrm{QM}}\varvec{\Lambda }_{\mathrm{QM}}\mathbf{U }_{\mathrm{QM}}'$ and $\mathbf{G }_{\mathrm{M}} = \mathbf{U }_{\mathrm{M}}\varvec{\Lambda }_{\mathrm{M}}\mathbf{U }_{\mathrm{M}}'$.

Before we proceed to define our scenarios of interest and evaluate $\kappa _{\mathrm{i}}$ for each of them, we provide a brief discussion about our assumptions for the true model. In the study by Jiang et al. [13], the authors assumed that both the number of QTL ($\hbox {q}$) and the number of markers ($\hbox {m}$) increase simultaneously with increasing SNP data density ($\hbox {q},\hbox {m}\rightarrow \infty$). Define $\mathbf{A }$ as the matrix of expected relationships between individuals, such that $\mathbb {E}(\mathbf{G }_{\mathrm{Q}}) = \mathbb {E}(\mathbf{G }) = \mathbf{A }$ [17]. Then $\lim _{\mathrm{q},\mathrm{m} \rightarrow \infty }\mathbf{G } = \lim _{\mathrm{q} \rightarrow \infty }\mathbf{G }_{\mathrm{Q}} = \mathbf{A }$, and using the eigen-decompositions $\mathbf{A } = \mathbf{U }_{\mathrm{A}}\varvec{\Lambda }_{\mathrm{A}}\mathbf{U }_{\mathrm{A}}'$, $\mathbf{G }_{\mathrm{Q}} = \mathbf{U }_{\mathrm{Q}}\varvec{\Lambda }_{\mathrm{Q}}\mathbf{U }_{\mathrm{Q}}'$ and $\mathbf{G } = \mathbf{U }\varvec{\Lambda }\mathbf{U }'$, we can state that $\lim _{\mathrm{q},\mathrm{m} \rightarrow \infty }\mathbf{U } = \lim _{\mathrm{q} \rightarrow \infty }\mathbf{U }_{\mathrm{Q}} = \mathbf{U }_{\mathrm{A}}$ and $\lim _{\mathrm{q},\mathrm{m} \rightarrow \infty }\varvec{\Lambda } = \lim _{\mathrm{q} \rightarrow \infty }\varvec{\Lambda }_{\mathrm{Q}} = \varvec{\Lambda }_{\mathrm{A}}$. Therefore, $\lim _{\mathrm{q},\mathrm{m} \rightarrow \infty }\hbox {g}(\gamma ) = \lim _{\mathrm{q} \rightarrow \infty }\hbox {g}_{_{\mathrm{Q}}}(\gamma ) = \hbox {g}_{_{\mathrm{A}}}(\gamma ) = \sum _{\mathrm{i}=1}^{\mathrm{n}-1}\sum _{\mathrm{j}=1}^{\mathrm{n}-1}(\mathbf{y }'\mathbf{U }_{\mathrm{Ai}})^{2}(\lambda _{\mathrm{Ai}} - \lambda _{\mathrm{Aj}})/[(1 + \gamma \lambda _{\mathrm{Ai}})^{2}(1 + \gamma \lambda _{\mathrm{Aj}})] + \hbox {n}\bar{\hbox {y}}^{2}\sum _{\mathrm{j}=1}^{\mathrm{n}-1}\lambda _{\mathrm{Aj}}/(1 + \gamma \lambda _{\mathrm{Aj}})$, meaning that the REML functions of the true and genomic models become equal with increasing SNP data density. Because $\lim _{\mathrm{n}\rightarrow \infty }\mathbb {E}(\hat{\gamma }_{_{\mathrm{Q}}})=\gamma _{_{\mathrm{Q}}}$ [6], if $\lim _{\mathrm{q},\mathrm{m} \rightarrow \infty }\hbox {g}(\gamma ) = \lim _{\mathrm{q} \rightarrow \infty }\mathrm{g}_{_{\mathrm{Q}}}(\gamma )$, it is straightforward that $\lim _{\mathrm{n}\rightarrow \infty }\mathbb {E}(\hat{\gamma })=\gamma _{_{\mathrm{Q}}}$.

We consider that the assumption that both $\hbox {q}$ and $\hbox {m}$ increase simultaneously with increasing SNP data density is too strong. Unless we consider the true model to be the infinitesimal model (in which a phenotype is generated by a countable infinite number of QTL, each with very small effect), it is most likely that the number of QTL will be finite (it may still be large, but finite). Thus we assumed a fixed and finite number of QTL for the true model, and therefore $\mathbf{G }_{\mathrm{Q}} \ne \mathbf{A }$. Our main objective was to evaluate how much of the variability in the phenotypes can be captured by $\mathbf{G }$, potentially leading to $\hat{\gamma }$ being biased to $\gamma _{_{\mathrm{Q}}}$.

We used simulations to support the theoretical analysis of the REML estimators of $\hbox {h}^{2}=\gamma _{_{\mathrm{Q}}}/(\gamma _{_{\mathrm{Q}}}+1)$ when using genomic models. A preliminary study indicated that 2000 individuals were enough to ensure the asymptotic properties of REML under the true model. The simulations were performed for eight scenarios that differed in population structure (completely unrelated or related individuals) and genetic architecture, in the linkage disequilibrium between QTL and markers, and the MAF of the QTL. We assumed independence between MAF and effect sizes when simulating QTL effects, and phenotypes were simulated using scaled genotypes, with a heritability parameter $\hbox {h}^{2}=0.05,0.15,\ldots ,0.95$. 20,000 SNPs were simulated and for each scenario we estimated the heritability for 500 replicates that assigned 100 SNPs as QTL, and for 500 replicates that assigned 3000 SNPs as QTL. The algorithms used for the simulations can be found in appendices G to J. For each scenario, the heritability was estimated using the true model (QTL only), and two genomic models, one containing the QTL plus the markers, and one containing the markers only.

## Results

### Conditions for unbiased or biased REML estimators

The key to evaluating the bias of $\hat{\gamma }$ to the true parameter $\gamma _{_{\mathrm{Q}}}$ is the term $\sum _{\mathrm{k}=1}^{\mathrm{n}}(\mathbf{U }_ {\mathrm{i}}'\mathbf{U }_{\mathrm{Qk}})^{2}\lambda _{\mathrm{Qk}}$ for every $\hbox {i}= 1,\ldots ,\hbox {n}$, in Eq. (5). This term corresponds to the diagonal of $\mathbf{U }'\mathbf{U }_{\mathrm{Q}}\varvec{\Lambda }_{\mathrm{Q}}\mathbf{U }_{\mathrm{Q}}'\mathbf{U }$. The off-diagonals of this matrix do not feature in the likelihood of the genomic models, as can be seen in Eq. (5), and thus, are not relevant to the bias of $\hat{\gamma }$. Nonetheless, we performed a brief analysis of the off-diagonal elements in our simulations, and verified that their values are centered at zero and within the interval (− 0.15,0.15), regardless of the scenario. To simplify notation, we define:

6 $$\begin{aligned} \sum _{\mathrm{k}=1}^{\mathrm{n}}(\mathbf{U }_ {\mathrm{i}}'\mathbf{U }_{\mathrm{Qk}})^{2}\lambda _{\mathrm{Qk}} = \kappa _{\mathrm{i}}, \quad \forall \quad \hbox {i}=1,\ldots ,\hbox {n}. \end{aligned}$$

Here, we set out the conditions for asymptotically unbiased or biased $\hat{\gamma }$, and in the following subsections we give details on how we arrived at these conditions:

1. $$\kappa _{\mathrm{i}} = \lambda _{\mathrm{i}}, \quad \forall \,\, \hbox {i}=1,\ldots ,\hbox {n} \quad \Longrightarrow \quad \lim _{\mathrm{n}\rightarrow \infty }\mathbb {E}(\hat{\gamma }) = \gamma _{_{\mathrm{Q}}}$$    (unbiased)
2. $\kappa _{\mathrm{i}} = \hbox {c}, \quad \forall \,\, \hbox {i}=1,\ldots ,\hbox {n} \quad \Longrightarrow \quad \lim _{\mathrm{n}\rightarrow \infty }\mathbb {E}(\hat{\gamma }) = 0$    ($\gamma _{_{\mathrm{Q}}}$ cannot be estimated)
3. $$\kappa _{\mathrm{i}}< \lambda _{\mathrm{i}}, {\text{ for most }} \hbox {i}=1,\ldots ,\hbox {n} \, \Longrightarrow \, \lim _{\mathrm{n}\rightarrow \infty }\mathbb {E}(\hat{\gamma }) < \gamma _{_{\mathrm{Q}}}$$    (biased: downwards)
4. $$\kappa _{\mathrm{i}}> \lambda _{\mathrm{i}}, {\text{ for most }} \hbox {i}=1,\ldots ,\hbox {n} \, \Longrightarrow \, \lim _{\mathrm{n}\rightarrow \infty }\mathbb {E}(\hat{\gamma }) > \gamma _{_{\mathrm{Q}}}$$    (biased: upwards)

Note that $\hbox {h}^{2}$ and $\hbox {h}^{2}_{\mathrm{gen}}$ are monotone increasing to $\gamma _{_{\mathrm{Q}}}$ and $\gamma$ respectively, meaning that if we have $0< \gamma _{-}< \gamma _{\mathrm{Q}} < \gamma _{+}$, such that $\hbox {h}^{2}_{-} = \gamma _{-}/(\gamma _{-}+1)$ and $\hbox {h}^{2}_{-} = \gamma _{+}/(\gamma _{+}+1)$, then $\hbox {h}^{2}_{-}< \hbox {h}^{2} < \hbox {h}^{2}_{+}$. Thus, the direction of the bias of $\hat{\hbox {h}}^{2}_{\mathrm{gen}}$ for $\hbox {h}^{2}$ is the same as that of the bias of $\hat{\gamma }$ for $\gamma _{_{\mathrm{Q}}}$.

#### Unbiased estimators

We know that $\lim _{\mathrm{n}\rightarrow \infty }\mathbb {E}(\hat{\gamma }_{_{\mathrm{Q}}})=\gamma _{_{\mathrm{Q}}}$ [6]. This holds for any number $\hbox {q}$ of QTL, regardless of their MAF and correlation between them. Moreover, in the non-observable REML equation, $\kappa _{\mathrm{Qi}} = \lambda _{\mathrm{Qi}}$. This means that for any set of eigen-values from any $\mathbf{G }$, for $\hbox {n}$ sufficiently large, $\lim _{\mathrm{n}\rightarrow \infty }\mathbb {E}(\hat{\gamma })=\gamma _{_{\mathrm{Q}}}$ if, and only if, the structure of the non-observable REML equation is as:

7 $$\begin{aligned} \sum _{\mathrm{i}=1}^{\mathrm{n}-1}\sum _{\mathrm{j}=1}^{\mathrm{n}-1}\frac{\lambda _{\mathrm{i}}}{(1 + \hat{\gamma }\lambda _{\mathrm{i}})^{2}}\left( \frac{\lambda _{\mathrm{i}} - \lambda _{\mathrm{j}}}{1 + \hat{\gamma }\lambda _{\mathrm{j}}}\right) = 0, \end{aligned}$$

meaning that $\hat{\gamma }$ is unbiased for $\gamma _{_{\mathrm{Q}}}$ if, and only if, $\kappa _{\mathrm{i}} = \lambda _{\mathrm{i}}$, for all $\hbox {i}=1,\ldots ,\hbox {n}$.

#### Biased estimators

There are two cases in which $\hat{\gamma }$ is biased to $\gamma _{_{\mathrm{Q}}}$: (1) $\kappa _{\mathrm{i}} = \hbox {c}$ for $\hbox {i}=1,\ldots ,\hbox {n}-1$, such that $\hbox {c}$ is a positive constant; (2) $\kappa _{\mathrm{i}} = \hbox {a}_{\mathrm{i}} \ne \lambda _{\mathrm{i}}$ for $\hbox {i}=1,\ldots ,\hbox {n}-1$, such that $\hbox {a}_{\mathrm{i}} > 0$. Note that because the $\mathbf{G }$ matrix is built with centered and scaled genotypes, its eigen-decomposition has $\hbox {n}-1$ degrees of freedom, and $\kappa _{\mathrm{n}} = \lambda _{\mathrm{n}} = 0$ always.

In the first case that $\hat{\gamma }$ is biased to $\gamma _{_{\mathrm{Q}}}$, where $\kappa _{\mathrm{i}} = \hbox {c}$ for $\hbox {i}=1,\ldots ,\hbox {n}-1$, we have:

8 $$\begin{aligned} \sum _{\mathrm{i}=1}^{\mathrm{n}-1}\sum _{\mathrm{j}=1}^{\mathrm{n}-1}\frac{\mathrm{c}}{(1 + \hat{\gamma }\lambda _{\mathrm{i}})^{2}}\left( \frac{\lambda _{\mathrm{i}} - \lambda _{\mathrm{j}}}{1 + \hat{\gamma }\lambda _{\mathrm{j}}}\right) = 0. \end{aligned}$$

Only $\hat{\gamma } = 0$ guarantees the identity in Eq. (8). Therefore, when $\kappa _{\mathrm{i}} = \hbox {c}$ for $\hbox {i}=1,\ldots ,\hbox {n}-1$, no variance from the genomic data can be captured by REML.

We now analyze the second case where $\kappa _{\mathrm{i}} = \hbox {a}_{\mathrm{i}}$ for $\hbox {i}=1,\ldots ,\hbox {n}-1$. If the relationship between $\hbox {a}_{\mathrm{i}}$ and $\lambda _{\mathrm{i}}$ is linear, *i.e.* $\hbox {a}_{\mathrm{i}} = \hbox {b}\lambda _{\mathrm{i}}$, then Eq. (7) ensures $\lim _{\mathrm{n}\rightarrow \infty }\mathbb {E}(\hat{\gamma })=\gamma _{_{\mathrm{Q}}}$. However, because $\sum _{\mathrm{i}=1}^{\mathrm{n}}\kappa _{\mathrm{i}} = \sum _{\mathrm{i}=1}^{\mathrm{n}}\lambda _{\mathrm{i}} = \mathrm{n}-1$, $\mathrm{a}_{\mathrm{i}}$ and $\lambda _{\mathrm{i}}$ cannot be linearly related, and $\hat{\gamma }$ will be biased to $\gamma _{_{\mathrm{Q}}}$. We have now:

9 $$\begin{aligned} \sum _{\mathrm{i}=1}^{\mathrm{n}-1}\sum _{\mathrm{j}=1}^{\mathrm{n}-1}\frac{\hbox {a}_{\mathrm{i}}}{(1 + \hat{\gamma }\lambda _{\mathrm{i}})^{2}}\left( \frac{\lambda _{\mathrm{i}} - \lambda _{\mathrm{j}}}{1 + \hat{\gamma }\lambda _{\mathrm{j}}}\right) = 0. \end{aligned}$$

To understand the bias in this case, we will go through some details about $\hbox {a}_{\mathrm{i}} > 0$. Let $\hbox {a}_{\mathrm{i}} = \lambda _{\mathrm{i}} + \hbox {b}_{\mathrm{i}}$, with $\hbox {b}_{\mathrm{i}} \ge -\lambda _{\mathrm{i}}$ and $\sum _{\mathrm{i}=1}^{\mathrm{n}}\hbox {b}_{\mathrm{i}} = 0$ (because $\sum _{\mathrm{i}=1}^{\mathrm{n}}\kappa _{\mathrm{i}} = \sum _{\mathrm{i}=1}^{\mathrm{n}}\lambda _{\mathrm{i}}$). Thus, $\hat{\gamma }$ satisfies

10 $$\begin{aligned} \sum _{\mathrm{i}=1}^{\mathrm{n}-1}\sum _{\mathrm{j}=1}^{\mathrm{n}-1}\frac{\lambda _{\mathrm{i}}}{(1 + \hat{\gamma }\lambda _{\mathrm{i}})^{2}}\left( \frac{\lambda _{\mathrm{i}} - \lambda _{\mathrm{j}}}{1 + \hat{\gamma }\lambda _{\mathrm{j}}}\right) + \sum _{\mathrm{i}=1}^{\mathrm{n}-1}\sum _{\mathrm{j}=1}^{\mathrm{n}-1}\frac{\hbox {b}_{\mathrm{i}}}{(1 + \hat{\gamma }\lambda _{\mathrm{i}})^{2}}\left( \frac{\lambda _{\mathrm{i}} - \lambda _{\mathrm{j}}}{1 + \hat{\gamma }\lambda _{\mathrm{j}}}\right) = 0. \end{aligned}$$

From the unbiased case, we know that $\sum _{\mathrm{i}=1}^{\mathrm{n}-1}\sum _{\mathrm{j}=1}^{\mathrm{n}-1}\lambda _{\mathrm{i}}(\lambda _{\mathrm{i}} - \lambda _{\mathrm{j}})/[(1 + \hat{\gamma }\lambda _{\mathrm{i}})^{2}(1 + \hat{\gamma }\lambda _{\mathrm{j}})]\mid _{\hat{\gamma } = \gamma _{\mathrm{Q}}} = 0$. This means that if $\sum _{\mathrm{i}=1}^{\mathrm{n}-1}\sum _{\mathrm{j}=1}^{\mathrm{n}-1}\mathrm{b}_{\mathrm{i}}(\lambda _{\mathrm{i}} - \lambda _{\mathrm{j}})/[(1 + \hat{\gamma }\lambda _{\mathrm{i}})^{2}(1 + \hat{\gamma }\lambda _{\mathrm{j}})] < 0$, (10) will hold only if $\sum _{\mathrm{i}=1}^{\mathrm{n}-1}\sum _{\mathrm{j}=1}^{\mathrm{n}-1}\lambda _{\mathrm{i}}(\lambda _{\mathrm{i}} - \lambda _{\mathrm{j}})/[(1 + \hat{\gamma }\lambda _{\mathrm{i}})^{2}(1 + \hat{\gamma }\lambda _{\mathrm{j}})] > 0$. Because the latter is monotone decreasing on $\hat{\gamma }$, only an estimator $\hat{\gamma } < \gamma _{\mathrm{Q}}$ will result in $\sum _{\mathrm{i}=1}^{\mathrm{n}-1}\sum _{\mathrm{j}=1}^{\mathrm{n}-1}\frac{\lambda _{\mathrm{i}}}{(1 + \hat{\gamma }\lambda _{\mathrm{i}})^{2}}\left( \frac{\lambda _{\mathrm{i}} - \lambda _{\mathrm{j}}}{1 + \hat{\gamma }\lambda _{\mathrm{j}}}\right)$ > 0. Now, if $\sum _{\mathrm{i}=1}^{\mathrm{n}-1}\sum _{\mathrm{j}=1}^{\mathrm{n}-1}\mathrm{b}_{i}(\lambda _{\mathrm{i}} - \lambda _{\mathrm{j}})/[(1 + \hat{\gamma }\lambda _{\mathrm{i}})^{2}(1 + \hat{\gamma }\lambda _{\mathrm{j}})] > 0$, then we have analogously that $\hat{\gamma } > \gamma _{\mathrm{Q}}$.

### Genomic models for scenarios of interest in quantitative genetics

#### QTL uncorrelated to markers

In “Appendix 5”, we show that for genomic models that include the QTL plus markers, in which markers are uncorrelated to the QTL,

11 $$\begin{aligned} \kappa _{_{\mathrm{QM}}\mathrm{i}} = \lambda _{\mathrm{QMi}} + \frac{\mathrm{m}}{\mathrm{q}}\left( \lambda _{\mathrm{QMi}} - 1 - \delta _{\mathrm{i}}\right) , \quad \forall \quad \mathrm{i}=1,\ldots ,\mathrm{n}-1, {\text{ with }} \mathbb {E}\left( \delta _{\mathrm{i}}\right) = 0. \end{aligned}$$

Assuming that the number of SNPs is always much larger than the number of individuals ($\hbox {q}+\hbox {m}>>>\hbox {n}$), $\lambda _{\mathrm{QM}1}>\cdots>\lambda _{\mathrm{QM},\mathrm{n}-1}>\lambda _{\mathrm{QMn}}=0$. Since $\sum _{\mathrm{i}=1}^{\mathrm{n}}\lambda _{\mathrm{QMi}} = \hbox {n}-1$ and because $\lambda _{\mathrm{QM1}},\ldots ,\lambda _{\mathrm{QMn}}$ follow the Mar$\breve{c}$enko-Pastur distribution when $\hbox {n}$ is sufficiently large [18], we have:

12 $$\begin{aligned} \left( 1-\sqrt{\frac{\hbox {n}}{\hbox {q}+\hbox {m}}}\right) ^{2}< \lambda _{\mathrm{QM},\mathrm{n}-1}< \cdots< \lambda _{\mathrm{QM}1} < \left( 1+\sqrt{\frac{\hbox {n}}{\hbox {q}+\hbox {m}}}\right) ^{2}. \end{aligned}$$

Note in Eq. (12) that increasing the number $\hbox {m}$ of markers will concentrate the eigen-values more strongly around 1. Hence, for $\hbox {m}$ very large, $\left( \lambda _{\mathrm{QMi}} - 1\right) \rightarrow 0$ at a faster rate than $\hbox {m}/\hbox {q}$ increases, and $\kappa _{_{\mathrm{QM}}\mathrm{i}} \rightarrow \lambda _{\mathrm{QMi}} - (\mathrm{m}/\mathrm{q})\delta _{\mathrm{i}}$. Since $\mathbb {E}\left( \delta _{\mathrm{i}}\right) = 0$, the ratio $\hbox {m}/\hbox {q}$ determines only the variance of $\kappa _{_{\mathrm{QM}}\mathrm{i}}$ around $\lambda _{\mathrm{QMi}}$. Therefore, a genomic model that contains QTL plus markers that are uncorrelated to the QTL will yield $\hat{\gamma }_{_{\mathrm{QM}}}$ such that $\lim _{\mathrm{n}\rightarrow \infty }\mathbb {E}(\hat{\gamma }_{_{\mathrm{QM}}})=\gamma _{_{\mathrm{Q}}}$.

We also show in “Appendix 5” that for genomic models that include markers only,

13 $$\begin{aligned} \kappa _{_{\mathrm{M}}\mathrm{i}} = 1 + \delta _{\mathrm{i}}, \quad \forall \quad \mathrm{i}=1,\ldots ,\mathrm{n}-1, {\text{ with }} \mathbb {E}\left( \delta _{\mathrm{i}}\right) = 0. \end{aligned}$$

Therefore, $\kappa _{\mathrm{i}}$ is a constant, and a genomic model that only contains markers that are uncorrelated to the QTL in the SNP data will always obtain $\hat{\gamma }_{\mathrm{M}} = 0$, when REML is used.

#### QTL correlated to markers

In “Appendix 6”, we show that for genomic models that include the QTL plus markers, in which markers are correlated to the QTL,

14 $$\begin{aligned} \kappa _{_{\mathrm{QM}}\mathrm{i}} = \lambda _{\mathrm{QMi}} + \delta _{\mathrm{i}}, \quad \forall \quad \mathrm{i}=1,\ldots ,\mathrm{n}-1, {\text{ with }} \mathbb {E}\left( \delta _{\mathrm{i}}\right) = \sum _{\mathrm{j}=1}^{\mathrm{n}}\sum _{\mathrm{l}=1}^{\mathrm{n}}(\sigma _{\mathrm{ijl,Qjl}} - \sigma _{\mathrm{ijl,jl}}), \end{aligned}$$

where $\sigma _{\mathrm{ijl},\mathrm{Qjl}} = \mathbb {C}\hbox {ov}(\hbox {U}_{\mathrm{QMij}}\hbox {U}_{\mathrm{QMil}},\hbox {G}_{\mathrm{Qjl}})$ and $\sigma _{\mathrm{ijl},\mathrm{jl}} = \mathbb {C}\hbox {ov}(\hbox {U}_{\mathrm{QMij}}\hbox {U}_{\mathrm{QMil}},\hbox {G}_{\mathrm{QMjl}})$. It is intuitive that $\sigma _{\mathrm{ijl},\mathrm{jl}} \ge \sigma _{\mathrm{ijl},\mathrm{Qjl}}$, and therefore $\mathbb {E}(\delta _{\mathrm{i}}) \le 0$. Thus, a genomic model that contains all QTL and markers that are correlated to the QTL will yield $\hat{\gamma }_{_{\mathrm{QM}}}$ such that $\lim _{\mathrm{n}\rightarrow \infty }\mathbb {E}(\hat{\gamma }_{_{\mathrm{QM}}})=\gamma _{_{\mathrm{Q}}}$, only when $\sigma _{\mathrm{ijl},\mathrm{jl}} \approx \sigma _{\mathrm{ijl},\mathrm{Qjl}}$, resulting in $\mathbb {E}(\delta _{\mathrm{i}}) \approx 0$.

$\mathbb {E}\left( \delta _{\mathrm{i}}\right)$ depends greatly on the distributions of the minor allele frequencies of the QTL and markers, $\hbox {f}_{\mathrm{MAF}(\mathrm{QTL})}$ and $\hbox {f}_{\mathrm{MAF}(\mathrm{markers})}$, respectively. When $\hbox {f}_{\mathrm{MAF}(\mathrm{QTL})} = \hbox {f}_{\mathrm{MAF}(\mathrm{markers})}$, unless the number of QTL is very small (say $\hbox {q} \le 10$), we find that $\mathbf{G }_{\mathrm{Q}}$ and $\mathbf{G }_{\mathrm{QM}}$ are very similar. It is intuitively obvious that in this case $\sigma _{\mathrm{ijl},\mathrm{jl}} \approx \sigma _{\mathrm{ijl},\mathrm{Qjl}}$. Hence, $\mathbb {E}\left( \delta _{\mathrm{i}}\right) \approx 0$, and consequently $\lim _{\mathrm{n}\rightarrow \infty }\mathbb {E}(\hat{\gamma }_{_{\mathrm{QM}}})=\gamma _{_{\mathrm{Q}}}$. When $\hbox {f}_{\mathrm{MAF}(\mathrm{QTL})} \ne \hbox {f}_{\mathrm{MAF}(\mathrm{markers})}$, the larger the number $\hbox {m}$ of markers, the more different $\mathbf{G }_{\mathrm{Q}}$ and $\mathbf{G }_{\mathrm{QM}}$ will be. Moreover, $\lim _{\mathrm{m}\rightarrow \infty }\mathbf{G }_{\mathrm{QM}} = \lim _{\mathrm{m}\rightarrow \infty }\mathbf{G }_{\mathrm{M}}$. This difference between $\mathbf{G }_{\mathrm{Q}}$ and $\mathbf{G }_{\mathrm{QM}}$ will ensure the inequality $\sigma _{\mathrm{ijl},\mathrm{jl}} \ge \sigma _{\mathrm{ijl},\mathrm{Qjl}}$. Hence, $\mathbb {E}\left( \delta _{\mathrm{i}}\right) < 0$, and consequently $\mathbb {E}(\hat{\gamma }_{_{\mathrm{QM}}}) < \gamma _{_{\mathrm{Q}}}$.

We show in “Appendix 4” that $\mathbf{G }_{\mathrm{Q}} = \mathbf{A } + \varvec{\Delta }_{\mathrm{Q}}$, with the number of QTL $\hbox {q}$ being fixed, $\mathbf{G }_{\mathrm{QM}} = \mathbf{A } + \varvec{\Delta }_{\mathrm{QM}}$, and $\mathbf{G }_{\mathrm{M}} = \mathbf{A } + \varvec{\Delta }_{\mathrm{M}}$. The relationship between individuals increases the speed of convergence of $\varvec{\Delta }_{\mathrm{QM}} \rightarrow \mathbf{0 }$ (when $\hbox {f}_{\mathrm{MAF}(\mathrm{QTL})} \ne \hbox {f}_{\mathrm{MAF}(\mathrm{markers})}$) and of $\varvec{\Delta }_{\mathrm{M}} \rightarrow \mathbf{0 }$, when $\hbox {m}$ increases. Therefore, $\mathbb {V}\hbox {ar}\left( \delta _{\mathrm{QMij}}\right)$ and $\mathbb {V}\hbox {ar}\left( \delta _{\mathrm{Mij}}\right)$ decreases when the number of generations increases. Finally, we find that $\mathbf{G }$ and $\mathbf{G }_{\mathrm{Q}}$ are more similar when the number of generations increases. Thus, the choice of populations with increasing relationship between individuals tends to reduce the bias of heritability estimators (when these are biased). However, when $\hbox {f}_{\mathrm{MAF}(\mathrm{QTL})} \ne \hbox {f}_{\mathrm{MAF}(\mathrm{markers})}$, stronger relationships are necessary, so the downward-bias becomes less perceptible. The explanation for this is related to the range of the MAF. For any $\mathbf{G } = \mathbf{A } + \varvec{\Delta }$, $\mathbb {V}\hbox {ar}\left( \delta _{\mathrm{ij}}\right)$ for SNPs with low MAF is lower than $\mathbb {V}\hbox {ar}\left( \delta _{\mathrm{ij}}\right)$ for SNPs with high MAF, and populations with more closely related individuals are necessary to compensate for this difference in $\mathbb {V}\mathrm{ar}\left( \delta _{\mathrm{ij}}\right)$ across different MAF ranges.

We also show in “Appendix 6” that for genomic models that include the markers only,

15 $$\begin{aligned} \kappa _{_{\mathrm{M}}\mathrm{i}} = 1 + \sum _{\mathrm{j}=1}^{\mathrm{n}}\mathrm{U}_{\mathrm{Mij}}^{2}\delta _{\mathrm{Qjj}} + \sum _{\mathrm{j}=1}^{\mathrm{n}}\sum _{\mathrm{l} \ne \mathrm{j}}\mathrm{U}_{\mathrm{Mij}}\mathrm{U}_{\mathrm{Mil}}\left( \mathrm{a}_{\mathrm{jl}} + \delta _{\mathrm{Qjl}}\right) , \quad \forall \quad \mathrm{i}=1,\ldots ,\mathrm{n}-1. \end{aligned}$$

Equation (15) is not so straightforward to understand analytically. Assume that we randomly pick just a few markers. These markers will most likely be in low LD with the QTL and $\kappa _{_{\mathrm{M}}\mathrm{i}} \approx 0$, as shown in the previous section. When the density of marker data increases, we obtain Eq. (15). We show in “Appendix 6” that $\lim _{\mathrm{m}\rightarrow \infty }\kappa _{_{\mathrm{M}}\mathrm{i}} = \lim _{\mathrm{m}\rightarrow \infty }\kappa _{_{\mathrm{QM}}\mathrm{i}}$. This means that $\lim _{\mathrm{n}\rightarrow \infty }\mathbb {E}(\hat{\gamma }_{_{\mathrm{M}}}) \le \lim _{\mathrm{n}\rightarrow \infty }\mathbb {E}(\hat{\gamma }_{_{\mathrm{QM}}})$, with equality only when $\hbox {m}\rightarrow \infty$.

#### Simulations

Table 1 summarizes what is expected regarding the estimation of the heritability for a set of scenarios that are relevant in quantitative genetics studies, based on the theory detailed in the section Conditions for unbiased REML estimators. The REML estimation of $\gamma$ was performed on data containing QTL plus markers ($\hat{\gamma }_{\mathrm{QM}}$) and markers only ($\hat{\gamma }_{\mathrm{M}}$). It is known that $\hat{\gamma }_{\mathrm{M}} < \hat{\gamma }_{\mathrm{QM}}$, since the markers alone cannot capture more genetic variability than SNP data that contains both QTL and markers [12]. However, for scenarios in which markers are in LD with the QTL $\lim _{\mathrm{m}\rightarrow \infty }\hat{\gamma }_{\mathrm{M}}=\lim _{\mathrm{m}\rightarrow \infty }\hat{\gamma }_{\mathrm{QM}}$. When individuals are completely unrelated, such convergence is most probably unrealistic even with sequence data (although $\hat{\gamma }_{\mathrm{M}}$ approaches $\hat{\gamma }_{\mathrm{QM}}$). In populations with strongly related individuals $\hat{\gamma }_{\mathrm{M}} \longrightarrow \hat{\gamma }_{\mathrm{QM}}$ for $\hbox {m}$ finite and sufficiently large.

Figure 1 presents the simulation results for scenarios 1 and 2, Fig. 2 presents the simulation results for scenarios 3, 4 and 5, and Fig. 3 presents the simulation results for scenarios 6, 7 and 8. All three figures show the results for simulations that assigned 3000 SNPs as QTL. The results for the simulations that assigned 100 SNPs as QTL differed from those presented in Figs. 1, 2 and 3 only by a larger variance around the same means. In all three figures, panel (a) shows the relationship between $\lambda _{\mathrm{i}}$ and $\kappa _{\mathrm{i}}$, for the true model (QTL only) and for both genomic models evaluated (QTL plus markers and markers only); the relationship in the simulated data agreed with the theory in the section Genomic models for scenarios of interest in quantitative genetics, for QTL uncorrelated and correlated to markers, respectively. In all three figures, panel (b) presents the confidence ellipses for the simulated heritabilities ($\hbox {h}_{\mathrm{sim}}^{2} = \gamma _{_{\mathrm{sim}}}/(1+\gamma _{_{\mathrm{sim}}})$), with a simulation parameter $\hbox {h}^{2}=0.05,0.15,\ldots ,0.95$, and the heritabilities estimated using REML ($\hbox {h}_{\mathrm{REML}}^{2} = \gamma _{_{\mathrm{REML}}}/(1+\gamma _{_{\mathrm{REML}}})$), for the true model (QTL only) and for both genomic models evaluated (QTL plus markers and markers only); $\hbox {h}_{\mathrm{sim}}^{2}$ was very stable around the simulation parameters, and $\hbox {h}_{\mathrm{REML}}^{2}$ had confidence intervals that agreed with the results in Table 1. Note that when QTL were correlated with markers, in scenarios 3 to 8, the variability of $\hbox {h}_{\mathrm{REML}}^{2}$ was smaller than that of $\hbox {h}_{\mathrm{REML}}^{2}$ when QTL were uncorrelated with markers, in scenarios 1 and 2. In all three figures, panel (c) presents the confidence ellipses for the simulated heritabilities ($\hbox {h}_{\mathrm{sim}}^{2} = \gamma _{_{\mathrm{sim}}}/(1+\gamma _{_{\mathrm{sim}}})$), with a simulation parameter $\hbox {h}^{2}=0.05,0.15,\ldots ,0.95$, and the relative bias of $\hbox {h}_{\mathrm{REML}}^{2}$ ($\hbox {RB}(\hbox {h}_{\mathrm{REML}}^{2}) = (\hbox {h}_{\mathrm{REML}}^{2}-\hbox {h}_{\mathrm{sim}}^{2})/\hbox {h}_{\mathrm{sim}}^{2}$). Note that when QTL were correlated with markers, in scenarios 3 to 8, the variability of $\hbox {RB}(\hbox {h}_{\mathrm{REML}}^{2})$ was smaller than that of $\hbox {RB}(\hbox {h}_{\mathrm{REML}}^{2})$ when QTL were uncorrelated with markers, in scenarios 1 and 2. Note as well, that the variability of $\hbox {RB}(\hbox {h}_{\mathrm{REML}}^{2})$ decreases when $\hbox {h}^{2}_{\mathrm{sim}}$ increases. For scenarios 6, 7 and 8, in which QTL were correlated with markers and $\hbox {f}_{\mathrm{MAF}(\mathrm{QTL})}\ne \hbox {f}_{\mathrm{MAF}(\mathrm{makers})}$, we found that increasing the number of generations (thus, increasing the relationship between simulated individuals) decreases the bias of estimation and the variability of $\hbox {h}^{2}_{\mathrm{REML}}$ and $\hbox {RB}(\hbox {h}_{\mathrm{REML}}^{2})$.

## Discussion

We have performed a theoretical analysis of the likelihood equations of genomic models based on splitting these equations into components in order to isolate and identify those that contribute to incorrect inferences. We have shown that the term in the likelihood equations that is responsible for producing potentially biased heritability estimators ($\hat{\hbox {h}}^{2}_{\mathrm{gen}}$) is in fact a measure that evaluates whether $\mathbf{G }$ provides a proper description of $\mathbf{G }_{\mathrm{Q}}$ or not.

The key measure to evaluate whether bias will arise in the REML heritability estimators is $\kappa _{\mathrm{i}} = \sum _{\mathrm{k}=1}^{\mathrm{n}}(\mathbf{U }_ {\mathrm{i}}'\mathbf{U }_{\mathrm{Qk}})^{2}\lambda _{\mathrm{Qk}}$, for every $\hbox {i}=1,\ldots ,\hbox {n}$, as we have shown in the section Conditions for unbiased REML estimators. Elements $\kappa _{1},\ldots ,\kappa _{\mathrm{n}}$ correspond to the diagonal of $\mathbf{U }'\mathbf{U }_{\mathrm{Q}}\varvec{\Lambda }_{\mathrm{Q}}\mathbf{U }_{\mathrm{Q}}'\mathbf{U }$, and the condition for unbiased $\hat{\hbox {h}}^{2}_{\mathrm{gen}}$ is that the portion of variance explained by the $\hbox {i-th}$ component of $\mathbf{G }$ (defined by $\lambda _{\mathrm{i}}$) is equivalent to the sum of weighted correlations between its corresponding eigen-vector and the eigen-vectors of $\mathbf{G }_{\mathrm{Q}}$, *i.e.* $\kappa _{\mathrm{i}} = \lambda _{\mathrm{i}}$. This identity is equivalent to saying that $\varvec{\Lambda }_{\mathrm{Q}}$ and $\varvec{\Lambda }$ are similar matrices in the general linear group of $\mathbf{U }_{\mathrm{Q}}'\mathbf{U }$. Evaluation of this similarity of $\varvec{\Lambda }_{\mathrm{Q}}$ and $\varvec{\Lambda }$ is much more informative than a direct comparison of the elements of $\mathbf{G }_{\mathrm{Q}}$ with those of $\mathbf{G }$, or a comparison of their eigen-values (see Additional file 1, which presents the distribution of $\lambda _{\mathrm{i}}$ and $\kappa _{\mathrm{i}}$, Additional files 2, 3 and 4, which present the scatterplots of $\lambda _{\mathrm{i}}$ versus $(\mathbf y '\mathbf U _{\mathrm{i}})^{2}$, and Additional files 5 and 6, which present respectively a scenario where $\lambda _{\mathrm{QMi}} \ne \lambda _{\mathrm{Qi}}$ and $\kappa _{\mathrm{QMi}} = \lambda _{\mathrm{QMi}}$ with $\mathbb {E}(\hat{\hbox {h}}^{2}_{\mathrm{QM}}) = \hbox {h}^{2}$, and a scenario where $\lambda _{\mathrm{Mi}} = \lambda _{\mathrm{Qi}}$ and $\kappa _{\mathrm{Mi}} \ne \lambda _{\mathrm{Mi}}$ with $\mathbb {E}(\hat{\hbox {h}}^{2}_{\mathrm{M}}) \ne \hbox {h}^{2}$). Hence, in the scenarios studied here, we can detect when a genomic relationship estimated by the SNPs correctly represents the true genetic relationships by verifying whether $\varvec{\Lambda }_{\mathrm{Q}}$ and $\varvec{\Lambda }$ are similar matrices in the general linear group of $\mathbf{U }_{\mathrm{Q}}'\mathbf{U }$, by comparing $\kappa _{\mathrm{i}}$ with $\lambda _{\mathrm{i}}$. This comparison allows us to determine the presence and direction of the bias, as described in the section Conditions for unbiased REML estimators.

Scenarios in which QTL and markers are in LE have been explored theoretically in other studies, with particular emphasis on the effect of the eigen-values of $\mathbf{G }$ on the likelihood of the (misspecified) genomic model [8, 13]. Although Kumar et al. [8] discussed the relevance of the difference between the eigen-vectors of $\mathbf{G }$ and $\mathbf{G }_{\mathrm{Q}}$, they did not relate this difference expressed by the correlation between the eigen-vectors, implicit in $\kappa _{\mathrm{i}}$, to the portion of variance explained by each $\lambda _{\mathrm{i}}$ and $\lambda _{\mathrm{Qk}}$. The performance of genomic models in estimating heritability was assessed mainly by describing the sensitivity of the likelihood to changes in the eigen-values, under the Mar$\breve{c}$enko–Pastur distribution [18]. Indeed, the likelihood depends sensitively on all the eigen-values, but evaluating the likelihood given a change in each eigen-value separately is not as informative as evaluating the REML equation given a change in the distribution of all eigen-values simultaneously.

Assuming that the number of individuals ($\hbox {n}$) is sufficiently large, and that the numbers of QTL and markers ($\hbox {q}$ and $\hbox {m}$) both increase with increasing density of SNP data such that $\lim _{\mathrm{n},\mathrm{q},\mathrm{m}\rightarrow \infty }\mathrm{n}/(\mathrm{q}+\mathrm{m})=\hbox {c}_{1} \in (0,1)$ and $\lim _{\mathrm{q},\mathrm{m}\rightarrow \infty }\mathrm{q}/\mathrm{m}=\mathrm{c}_{2} \in (0,1)$, Jiang et al. [13] used the Mar$\breve{c}$enko–Pastur distribution of the eigen-values to evaluate the limiting behavior of the term $\mathbf{P }\mathbf{G }\mathbf{P }/\hbox {tr}\left( \mathbf{P }\mathbf{G }\right) - \mathbf{P }^{2}/\hbox {tr}\left( \mathbf{P }\right)$ in the REML Eq. (3) of the genomic model, proving that under these assumptions, $\hat{\hbox {h}}^{2}_{\mathrm{gen}}$ is unbiased and consistent. Although Jiang et al. [13] stated that $\hat{\hbox {h}}^{2}_{\mathrm{gen}}$ still remains unbiased and consistent when QTL and markers are in LD, we have raised a particular concern about inferences of $\hbox {h}^{2}$ in the case when MAF(QTL) $\ne$ MAF(markers), such as when QTL are rare mutations, for which the estimates of heritability are empirically known to be biased [1, 4, 8, 19]. Two remarks about the approach used in [13] must be made at this stage. First, the limiting behavior of $\mathbf{P }\mathbf{G }\mathbf{P }/\hbox {tr}\left( \mathbf{P }\mathbf{G }\right) - \mathbf{P }^{2}/\hbox {tr}\left( \mathbf{P }\right)$ relies strongly on the Mar$\breve{c}$enko–Pastur distribution, which holds only when the SNPs are in complete LE (and thus individuals are unrelated, since family relationships would induce LD). The second remark is that LD between markers and QTL and the distribution of their MAFs may alter correlations between phenotypes and genotypes, implied in $\mathbf{y }'\mathbf{P }\mathbf{G }\mathbf{P }\mathbf{y }$ and $\mathbf{y }'\mathbf{P }\mathbf{y }$, and this was not evaluated by Jiang et al. [13], whereas in our study the correlations between phenotypes and genotypes are implied in $\kappa _{\mathrm{i}}$ (see "Appendix 2 and 3"). Using our approach and the result that $\lim _{\mathrm{q},\mathrm{m}\rightarrow \infty }\mathbf{G } = \lim _{\mathrm{q}\rightarrow \infty }\mathbf{G }_{\mathrm{Q}} = \mathbf{A }$ [17], we demonstrated in section Genomic models for scenarios of interest in quantitative genetics, that the conclusions from Jiang et al. [13] about $\hat{\hbox {h}}^{2}_{\mathrm{gen}}$ are mathematically true, even when QTL and markers are in LD. However, in populations of unrelated individuals with QTL as rare mutations, an unrealistically large number (tending to infinity) of QTL would be necessary to ensure $\lim _{\mathrm{q}\rightarrow \infty }\mathbf{G }_{\mathrm{Q}}=\mathbf{A }$, and when we assume that the number of QTL is finite, $\lim _{\mathrm{m}\rightarrow \infty }\mathbf{G } \ne \mathbf{G }_{\mathrm{Q}}$.

With the knowledge that the method proposed by Yang et al. [1] may yield a biased $\hat{\hbox {h}}^{2}_{\mathrm{gen}}$ under some scenarios, several approaches have been proposed for solving the problem of biased estimates, exploring different genetic architectures of the trait and population structure. The theory presented in our study can be adapted to provide an explanation for the success of those approaches, and we offer an overview on how that can be done for five approaches.

First, addressing the different MAF of the SNPs, Speed et al. [4] suggests a weighting of the SNPs by their MAF, which would give the same weighting to terms involving $\gamma$ in the non-observable REML functions, owing to the change in the definition of the heritability. A G-matrix obtained using the SNPs suitably weighted according to the scenario will improve the relationship between $\kappa _{\mathrm{i}}$ and $\lambda _{\mathrm{i}}$, reducing the bias of $\hat{\hbox {h}}^{2}_{\mathrm{gen}}$. The definition of a suitable weight must be explored further, and the theory provided in this study provides a tool that can be used for such investigations. The genomic model in (1) can be generalized to assume different weights to the SNPs by simply changing the assumption for the distribution of $\mathbf{b }$ to $\mathbf{b }\sim \hbox {N}(\mathbf{0 },\mathbf{D }\sigma ^{2}_{\mathrm{b}})$, such that $\mathbf{D }$ is a diagonal matrix of weights. The G-matrix must then be defined as $\mathbf{G } = \mathbf{WDW'}/\hbox {tr}(\mathbf{D })$ to ensure that properties indicated in “Appendix 1” and theoretical evaluations in the Results section hold.

Second, and with the same objective of distinguishing SNPs by their MAF, Yang et al. [19] suggested a method that is analogous to that proposed in [1] by fitting the model with several genomic variance components, each of them relative to groups of SNPs with MAF values within the same range. In this approach, assuming the components to be independent, we can obtain a non-observable REML equation for each genomic variance component to be estimated, and the analysis then follows exactly as we have presented here. This approach also generalizes the genomic model in Eq. (1) by assuming $\mathbf{b }\sim \hbox {N}(\mathbf{0 },\mathbf{D }\sigma ^{2}_{\mathrm{b}})$, as described in the previous paragraph, with the difference that the values on the diagonal of $\mathbf{D }$ are to be estimated. Indeed the method is capable of removing the bias of $\hat{\hbox {h}}^{2}_{\mathrm{gen}}$. However, as observed by [19], the increase in the number of variance components may increase the variance of $\hat{\hbox {h}}^{2}_{\mathrm{gen}}$, especially when independence between the components cannot be ensured, and, depending on the scenario evaluated, the estimates may be less reliable than those obtained by fitting a single genomic variance component.

Edwards et al. [20] suggested a third approach, which fits genomic models by including a variance component for SNPs grouped based on genomic features (i.e. genes and their gene ontology) to the model, which requires the use of prior information about the genomic data. The results of their study showed that a relevant amount of variance was attributed to the significant feature, and $\hat{\hbox {h}}^{2}_{\mathrm{gen}}$ in this approach can be evaluated with a non-observable REML equation for each component (SNPs grouped based on genomic features and SNPs not grouped based on genomic features), just as we suggest for evaluating $\hat{\hbox {h}}^{2}_{\mathrm{gen}}$ obtained with the approach proposed by [19]. It is important to note that the genomic feature model works better than a single component when the feature component is enriched for the QTL; otherwise, this model can also lead to problems in the estimation of $\hbox {h}^{2}$. The advantage of grouping SNPs based on genomic features instead of MAF is that there are fewer variance components, reducing the variance of $\hat{\hbox {h}}^{2}_{\mathrm{gen}}$. The use of prior genomic information to fit genomic models with mutiple genomic variance components was previously suggested by Speed and Balding [21], who included a dynamic procedure to define a suitable partition of SNPs.

A fourth approach considers the situation where prior genomic feature information is absent and Bayesian mixture models, such as BayesB [22] or BayesR [23], are reasonable solutions for assigning different distributions to groups of SNP effects [20]. Again, non-observable REML equations for each component can be used to evaluate $\hat{\hbox {h}}^{2}_{\mathrm{gen}}$ based on the assumptions posed by the Bayesian mixture models, and the assumptions can be tuned using the information from our suggested theoretical analysis.

A fifth approach includes related individuals to study populations, which can greatly reduce the bias of $\hat{\hbox {h}}^{2}_{\mathrm{gen}}$, when it exists. This is because rare QTL induce genetic relationships between individuals. In populations of nominally unrelated individuals the common markers will disguise those induced genetic relationships at the QTL ($\lim _{\mathrm{m}\rightarrow \infty }\mathbf{G }_{\mathrm{QM}}=\mathbf{A }=\mathbf{I }_{\mathrm{n}}\ne \mathbf{G }_{\mathrm{Q}}$), drastically reducing the correlations between eigen-vectors $(1/\hbox {n})\mathbf{U }_{\mathrm{i}}'\mathbf{U }_{\mathrm{Qk}}$ and resulting in $\kappa _{\mathrm{i}} < \lambda _{\mathrm{i}}$. Conversely, in populations of related individuals, assuming no selection, the induced genetic relationships at the QTL will better reflect the kinship matrix ($\mathbf{G }_{\mathrm{Q}}\approx \mathbf{A }$), improving the correlation between eigen-vectors $(1/\mathrm{n})\mathbf{U }_{\mathrm{i}}'\mathbf{U }_{\mathrm{Qk}}$ and resulting in less biased $\hat{\hbox {h}}^{2}_{\mathrm{gen}}$.

A last point to be raised in this discussion, concerns the direction of the bias of $\hat{\hbox {h}}^{2}_{\mathrm{gen}}$. We show in the section Genomic models for scenarios of interest in quantitative genetics, with our theoretical analysis, that when we consider a single genomic component in the model to estimate heritability (assuming SNP effects are all *i.i.d.*), when it exists, the bias of the estimator will tend to be downward. An exception is observed when $\hbox {f}_{\mathrm{MAF}_{_{\mathrm{QTL}}}} \ne \hbox {f}_{\mathrm{MAF}_{_{\mathrm{markers}}}}$ and the number of QTL is smaller than the number of individuals. If genomic models are fitted including the QTL and markers, such that the total number of SNPs in the genomic data is smaller than the number of individuals, the heritability estimator will present an upwards bias. This fact is related to $\hbox {rank}(\mathbf{G }_{\mathrm{QM}})<\hbox {n}-1$, as eigen-values that are zero are overestimated by $\kappa _{\mathrm{i}}$. Increasing the number of markers will make $\hbox {rank}(\mathbf{G }_{\mathrm{QM}})$ approach $\hbox {n}-1$, forcing only the last eigen-value to zero and the overestimation will no longer be present (see Additional file 7).

When multiple genomic components are considered in the model, overestimation of heritability may be observed even when the total number of SNPs is larger than the number of individuals. When different variance parameters are estimated for each component, the multiple components approach is explicit, and overestimation of heritability will relate to components with a rank lower than $\hbox {n}-1$. When a single variance parameter is estimated for SNPs associated with pre-determined weights, the multiple component approach is implicit, and overestimation of heritability as observed in [24] may relate to $\kappa _{\mathrm{i}}$ overestimating the largest $\lambda _{\mathrm{i}}$. Associating pre-determined weights to the SNPs in the genomic model may inflate correlations $\mathbf{U }_{\mathrm{i}}'\mathbf{U }_{\mathrm{Qk}}$ for eigen-vectors $\mathbf{U }_{\mathrm{i}}$ that are associated with the highest eigen-values, resulting in $\kappa _{\mathrm{i}} > \lambda _{\mathrm{i}}$, while correlations $\mathbf{U }_{\mathrm{i}}'\mathbf{U }_{\mathrm{Qk}}$ for eigen-vectors $\mathbf{U }_{\mathrm{i}}$ that are associated with the lowest eigen-values will be deflated, resulting in $\kappa _{\mathrm{i}} < \lambda _{\mathrm{i}}$ (see Additional file 8).

## Conclusions

In a Gaussian setup, the likelihood of a genomic model is misspecified with respect to that of the true model that conceptually generated the data. When used for inferring variance parameters the misspecified likelihood may yield biased estimators of those parameters, and inferences must be interpreted with caution. Misspecification of the likelihood is due to the difference between the covariance structures of the data specified by the misspecified and true models ($\mathbf{G }$ and $\mathbf{G }_{\mathrm{Q}}$), and our study shows that the bias of REML estimators of variance parameters is linked to the relationship between the eigen-values and eigen-vectors of both models, occurring when $\kappa _{\mathrm{i}} = \sum _{\mathrm{k}=1}^{\mathrm{n}}(\mathbf{U }_ {\mathrm{i}}'\mathbf{U }_{\mathrm{Qk}})^{2}\lambda _{\mathrm{Qk}} \ne \lambda _{\mathrm{i}}$. Moreover, comparison of $\kappa _{\mathrm{i}}$ with the eigen-value $\lambda _{\mathrm{i}}$ not only identifies the potential bias of variance components estimators, but is also a very informative method for comparing $\mathbf{G }$ with $\mathbf{G }_{\mathrm{Q}}$. The eigen-vectors reflect how each individual contributes to the proportion of variance explained by the components of $\mathbf{G }$ and $\mathbf{G }_{\mathrm{Q}}$ (defined by $\lambda _{\mathrm{i}}$ and $\lambda _{\mathrm{Qk}}$), and if the contributions are similar, then $\kappa _{\mathrm{i}} \approx \lambda _{\mathrm{i}}$, meaning that the covariance structures of the data specified by the genomic and the true models are equivalent. In mathematical terms, $\kappa _{\mathrm{i}} = \lambda _{\mathrm{i}}$ is the same as stating that $\varvec{\Lambda }_{\mathrm{Q}}$ and $\varvec{\Lambda }$ are similar matrices in the general linear group of $\mathbf{U }_{\mathrm{Q}}'\mathbf{U }$. We have evaluated the similarity of $\varvec{\Lambda }_{\mathrm{Q}}$ and $\varvec{\Lambda }$ in a set of scenarios of interest to quantitative genetics studies, identifying those for which inferences must be interpreted with caution. Because of the many factors related to the genetic architecture that influence the similarity of $\varvec{\Lambda }_{\mathrm{Q}}$ and $\varvec{\Lambda }$ (LD between QTL and markers, presence and number of QTL in the SNP data, MAF, relationship between individuals) and the lack of information about the QTL, quantifying the bias (when it exists) of the estimators of variance parameters, is not trivial. Although the quantification of this bias is complex, we can determine that in genomic models that consider a single genomic component to estimate heritability (assuming SNP effects are all *i.i.d.*), the bias of the estimator will tend to be downward, when it exists.

### Additional files

**Additional file 1.** Distribution of $\lambda_{{\rm i}}$ and $\kappa_{{\rm i}}$ for the eight scenarios, when considering 3000 QTL and 2000 individuals. Distributions are presented for ${\mathbf{G}}$ assuming the true model (QTL only) and assuming both genomic models evaluated (QTL plus markers and markers only).

**Additional file 2.** Scatterplot of $\lambda_{{\rm i}}$ versus $({\mathbf{y}}\prime{\mathbf{U}}_{{\rm i}})^{2}$ for one replicate on all the eight scenarios, when considering 3000 QTL and 2000 individuals. Distributions are presented for ${\mathbf{G}}$ assuming the true model (QTL only).

**Additional file 3.** Scatterplot of $\lambda_{{\rm i}}$ versus $(\mathbf{y}\prime \mathbf{U}_{{\rm i}})^{2}$ for one replicate on all the eight scenarios, when considering 3000 QTL and 2000 individuals. Distributions are presented for ${\mathbf{G}}$ assuming the genomic model with markers only.

**Additional file 4.** Scatterplot of $\lambda_{{\rm i}}$ versus $(\mathbf{y}\prime \mathbf{U}_{{\rm i}})^{2}$ for one replicate on all the eight scenarios, when considering 3000 QTL and 2000 individuals. Distributions are presented for ${\mathbf{G}}$ assuming the genomic model with QTL plus markers.

**Additional file 5.** Comparison of ${\mathbf{G}}$ to ${\mathbf{G}}_{{\rm Q}}$ for 2000 completely unrelated individuals by: (1) direct comparison of values, (2) comparison of eigen-values ($\lambda_{{\rm i}}$), and (3) comparison of $\lambda_{{\rm i}}$ to $\kappa_{{\rm i}}$, and comparison of $\hat{\hbox{h}}^{2}_{{\rm REML}}$ to $\hbox{h}^{2}$ simulated with parameter 0.25,0.5,0.75, for 100 replicates of each simulation parameter. ${\mathbf{G}}_{{\rm Q}}$ consisted of 100 QTL and ${\mathbf{G}}$ consisted of these 100 QTL plus 1900 markers in complete LE with the QTL, such that $\hbox{f}_{{\rm MAF}_{_{{\rm QTL}}}} \neq {\rm f}_{{\rm MAF}_{_{{\rm markers}}}}$.

**Additional file 6.** Comparison of ${\mathbf{G}}$ to ${\mathbf{G}}_{{\rm Q}}$ for 2000 completely unrelated individuals by: (1) direct comparison of values, (2) comparison of eigen-values ($\lambda_{{\rm i}}$), and (3) comparison of $\lambda_{{\rm i}}$ to $\kappa_{{\rm i}}$, and comparison of $\hat{\hbox{h}}^{2}_{{\rm REML}}$ to $\hbox{h}^{2}$ simulated with parameter 0.25,0.5,0.75, for 100 replicates of each simulation parameter. ${\mathbf{G}}_{{\rm Q}}$ consisted of 3000 QTL and ${\mathbf{G}}$ consisted of 3000 markers in complete LE with the QTL, such that $\hbox{f}_{{\rm MAF}_{_{{\rm QTL}}}} \neq \hbox{f}_{{\rm MAF}_{_{{\rm markers}}}}$.

**Additional file 7.** 95\% confidence intervals, based on 100 replicates, for the absolute bias of $\hat{\hbox{h}}^{2}_{{\rm REML}}$ to $\hbox{h}^{2}$. Genomic data consisted of QTL plus markers for 2000 completely unrelated individuals. The number of markers in the SNP data varied from 0 to 19,900, and 100 QTL were used to simulate the phenotypes with $\hbox{h}^{2}$ parameter 0.6. QTL and markers were in LD, such that $\hbox{f}_{{\rm MAF}_{_{{\rm QTL}}}} \neq \hbox{f}_{{\rm MAF}_{_{{\rm markers}}}}$.

**Additional file 8.** Relationship between $\lambda_{{\rm i}}$ and $\kappa_{{\rm i}}$, and absolute bias of $\hat{\hbox{h}}^{2}_{{\rm REML}}$ to $\hbox{h}^{2}$. Genomic data consisted of QTL plus markers for 2000 completely unrelated individuals. 100 QTL were used to simulate the phenotypes with $\hbox{h}^{2}$ parameter 0.05,0.15,…,0.95. QTL and markers were in LD, such that $\hbox{f}_{{\rm MAF}_{_{{\rm QTL}}}} \neq \hbox{f}_{{\rm MAF}_{_{{\rm markers}}}}$. Markers were weighted according to their LD with the QTL.

## Appendix 1: REML equation to REML function

Given any SNP genotypes matrix $\mathbf{W }$ with $\hbox {s}$ SNPs, we define $\mathbf{G } = \hbox {s}^{-1}\mathbf{W }\mathbf{W }' = \mathbf{U }\varvec{\Lambda }\mathbf{U }'$ where $\mathbf{U }$ and $\varvec{\Lambda }$ are the eigen-decomposition matrices of $\mathbf{G }$, the variance matrix $\mathbf{V } = \gamma \mathbf{G } + \mathbf{I }_{\mathrm{n}}$, such that *n* is the number of individuals in the population, and $\mathbf{P } = \mathbf{V }^{-1} - \mathbf{V }^{-1}\mathbf{1 }_{\mathrm{n}}\left( \mathbf{1 }_{\mathrm{n}}'\mathbf{V }^{-1}\mathbf{1 }_{\mathrm{n}}\right) ^{-1}\mathbf{1 }_{\mathrm{n}}'\mathbf{V }^{-1}$. Using the eigen-decomposition of $\mathbf{G }$, it is straightforward that $\mathbf{V }^{-1} = \mathbf{U }\left( \gamma \varvec{\Lambda } + \mathbf{I }_{\mathrm{n}}\right) ^{-1}\mathbf{U }'$. Thus,

16 $$\begin{aligned} \mathbf{P } = \mathbf{U }\left[ \left( \gamma \varvec{\Lambda } + \mathbf{I }_{\mathrm{n}}\right) ^{-1} - \frac{\left( \gamma \varvec{\Lambda } + \mathbf{I }_{\mathrm{n}}\right) ^{-1}\mathbf{U }'\mathbf{1 }_{\mathrm{n}}\mathbf{1 }_{\mathrm{n}}'\mathbf{U }\left( \gamma \varvec{\Lambda } + \mathbf{I }_{\mathrm{n}}\right) ^{-1}}{\mathbf{1 }_{\mathrm{n}}'\mathbf{U }\left( \gamma \varvec{\Lambda } + \mathbf{I }_{\mathrm{n}}\right) ^{-1}\mathbf{U }'\mathbf{1 }_{\mathrm{n}}}\right] \mathbf{U }', \end{aligned}$$

and since $\left( \gamma \varvec{\Lambda } + \mathbf{I }_{\mathrm{n}}\right) ^{-1}\mathbf{U }'\mathbf{1 }_{\mathrm{n}}\mathbf{1 }_{\mathrm{n}}'\mathbf{U }\left( \gamma \varvec{\Lambda } + \mathbf{I }_{\mathrm{n}}\right) ^{-1}$ has elements $\hbox {n}^{2}\bar{\hbox {U}}_{\mathrm{i}}\bar{\hbox {U}}_{\mathrm{j}}/\left[ (1+\gamma \lambda _{\mathrm{i}})(1+\gamma \lambda _{\mathrm{j}})\right]$, and the scalar $\mathbf{1 }_{\mathrm{n}}'\mathbf{U }\left( \gamma \varvec{\Lambda } + \mathbf{I }_{\mathrm{n}}\right) ^{-1}\mathbf{U }'\mathbf{1 }_{\mathrm{n}} = \hbox {n}^{2}\sum _{\mathrm{i}=1}^{\mathrm{n}}\bar{\hbox {U}}_{\mathrm{i}}^{2}/(1+\gamma \lambda _{\mathrm{i}})$,

17 $$\begin{aligned} \mathbf{P } = \mathbf{U }\left\{ \left( \gamma \varvec{\Lambda } + \mathbf{I }_{\mathrm{n}}\right) ^{-1} - \left( \sum _{\mathrm{i}=1}^{\mathrm{n}}\frac{\bar{\mathrm{U}}_{\mathrm{i}}^{2}}{1+\gamma \lambda _{\mathrm{i}}}\right) ^{-1}\left[ \frac{\bar{\mathrm{U}}_{\mathrm{i}}\bar{\hbox {U}}_{\mathrm{j}}}{(1+\gamma \lambda _{\mathrm{i}})(1+\gamma \lambda _{\mathrm{j}})}\right] _{\mathrm{i},\mathrm{j}=1}^{\mathrm{n}}\right\} \mathbf{U }'. \end{aligned}$$

We use the following properties of the eigen-decomposition, when $\hbox {n}$ is sufficiently large, to go through the next steps:

**ED1.**    $$\lambda _{1} \ge \cdots \ge \lambda _{\mathrm{n}-1} \ge \lambda _{\mathrm{n}} = 0$$;

**ED2.**    $$\mathrm{s} < \mathrm{n} \Longrightarrow \lambda _{\mathrm{i}} = 0$$, $$\forall$$ $$\mathrm{i} > s$$;

**ED3.**    $$\sum _{\mathrm{i}=1}^{\mathrm{n}}\lambda _{\mathrm{i}} = \mathrm{n}-1$$;

**ED4.**    $$\mathbf{U } = [\mathbf{U }_{\mathrm{1}} \cdots \mathbf{U }_{\mathrm{n}}]:$$ $$\mathbf{U }_{\mathrm{i}}' = [\hbox {U}_{\mathrm{i1}} \cdots \hbox {U}_{\mathrm{in}}]$$, $$\forall$$ $$\hbox {i}=1,\ldots ,\mathrm{n}$$;

**ED5.**    $$\lambda _{\mathrm{i}} > 0 \Longrightarrow \mathbf{1 }_{\mathrm{n}}'\mathbf{U }_{\mathrm{i}} = \sum _{\mathrm{j}=1}^{\mathrm{n}}\mathrm{U}_{\mathrm{ij}} = \mathrm{n}\bar{\hbox {U}}_{\mathrm{i}} = 0$$;

**ED6.**    $$\sum _{\mathrm{i}=1}^{\mathrm{n}}\left( \sum _{\mathrm{j}=1}^{\mathrm{n}}\mathrm{U}_{\mathrm{ij}}\right) ^{2} = \mathrm{n} \Longrightarrow \sum _{\mathrm{i}=1}^{\mathrm{n}}\bar{\mathrm{U}}_{\mathrm{i}}^{2} = \mathrm{n}^{-2}\sum _{\mathrm{i}=1}^{\mathrm{n}}\left( \sum _{\mathrm{j}=1}^{\mathrm{n}}\mathrm{U}_{\mathrm{ij}}\right) ^{2} = \mathrm{n}^{-1}$$.

Thus, we have now that

18 $$\begin{aligned} \mathbf{P } = \mathbf{U }\left\{ \left( \gamma \varvec{\Lambda } + \mathbf{I }_{\mathrm{n}}\right) ^{-1} - \left( \sum _{\mathrm{i}=1}^{\mathrm{n}}\bar{\hbox {U}}_{\mathrm{i}}^{2}\mathbb {I}_{\left\{ \lambda _{\mathrm{i}} = 0\right\} }\right) ^{-1}\left[ \bar{\hbox {U}}_{\mathrm{i}}\bar{\hbox {U}}_{\mathrm{j}}\right] _{\mathrm{i},\mathrm{j}=1}^{\mathrm{n}}\right\} \mathbf{U }' = \mathbf{U }\varvec{\Gamma }\mathbf{U }', \end{aligned}$$

where $$\varvec{\Gamma } = \left( \gamma \varvec{\Lambda } + \mathbf{I }_{\mathrm{n}}\right) ^{-1} - \hbox {n}\left[ \bar{\hbox {U}}_{\mathrm{i}}\bar{\hbox {U}}_{\mathrm{k}}\right] _{\mathrm{i},\mathrm{k}=1}^{\mathrm{n}}$$. Therefore,

19 $$\begin{aligned} \mathbf{y }'\left[ \frac{\mathbf{P }\mathbf{G }\mathbf{P }}{\hbox {tr}\left( \mathbf{P }\mathbf{G }\right) } - \frac{\mathbf{P }^{2}}{\hbox {tr}\left( \mathbf{P }\right) }\right] \mathbf{y } = \mathbf{y }'\left[ \frac{\mathbf{U }\varvec{\Gamma \Lambda \Gamma }\mathbf{U }'}{\hbox {tr}\left( \varvec{\Gamma \Lambda }\right) } - \frac{\mathbf{U }\varvec{\Gamma }^{2}\mathbf{U }'}{\hbox {tr}\left( \varvec{\Gamma }\right) }\right] \mathbf{y } = \frac{\mathbf{y }'\mathbf{U }\varvec{\Gamma \Lambda \Gamma }\mathbf{U }'\mathbf{y }}{\hbox {tr}\left( \varvec{\Gamma \Lambda }\right) } - \frac{\mathbf{y }'\mathbf{U }\varvec{\Gamma }^{2}\mathbf{U }'\mathbf{y }}{\hbox {tr}\left( \varvec{\Gamma }\right) }, \end{aligned}$$

where,

1. $$\varvec{\Gamma }\varvec{\Lambda }\varvec{\Gamma } = \mathbf{diag }\left( \frac{\lambda _{1}}{(\gamma \lambda _{1}+1)^{2}},\ldots ,\frac{\lambda _{\mathrm{n}}}{(\gamma \lambda _{\mathrm{n}}+1)^{2}}\right) \Longrightarrow \mathbf{y }'\mathbf{U }\varvec{\Gamma \Lambda \Gamma }\mathbf{U }'\mathbf{y } = \sum _{\mathrm{i}=1}^{\mathrm{n}}\left( \frac{\mathbf{y }'\mathbf{U }_{\mathrm{i}}}{\gamma \lambda _{\mathrm{i}}+1}\right) ^{2}\lambda _{\mathrm{i}}$$,
2. $$\varvec{\Gamma }^{2} = \left( \gamma \varvec{\Lambda } + \mathbf{I }_{\mathrm{n}}\right) ^{-2} - \hbox {n}\left[ \bar{\hbox {U}}_{\mathrm{i}}\bar{\hbox {U}}_{\mathrm{k}}\right] _{\mathrm{i},\mathrm{k}=1}^{\mathrm{n}} \Longrightarrow \mathbf{y }'\mathbf{U }\varvec{\Gamma ^{2}}\mathbf{U }'\mathbf{y } = \sum _{\mathrm{i}=1}^{\mathrm{n}}\left( \frac{\mathbf{y }'\mathbf{U }_{\mathrm{i}}}{\gamma \lambda _{\mathrm{i}}+1}\right) ^{2} - \hbox {n}\bar{\hbox {y}}^{2}$$,
3. $$\hbox {tr}\left( \varvec{\Gamma \Lambda }\right) = \sum _{\mathrm{i}=1}^{\mathrm{n}}\frac{\lambda _{\mathrm{i}}}{\gamma \lambda _{\mathrm{i}}+1} = \sum _{\mathrm{i}=1}^{\mathrm{n}-1}\frac{\lambda _{\mathrm{i}}}{\gamma \lambda _{\mathrm{i}}+1}$$,
4. $$\hbox {tr}\left( \varvec{\Gamma }\right) = \sum _{\mathrm{i}=1}^{\mathrm{n}}\frac{1}{\gamma \lambda _{\mathrm{i}}+1} - 1 = \sum _{\mathrm{i}=1}^{\mathrm{n}-1}\frac{1}{\gamma \lambda _{\mathrm{i}}+1}$$.

Now, being $\hat{\gamma }$ the solution of $\mathbf{y }'\mathbf{U }\varvec{\Gamma \Lambda \Gamma }\mathbf{U }'\mathbf{y }/\hbox {tr}\left( \varvec{\Gamma \Lambda }\right) - \mathbf{y }'\mathbf{U }\varvec{\Gamma }^{2}\mathbf{U }'\mathbf{y }/\hbox {tr}\left( \varvec{\Gamma }\right) = 0$, then,

20 $$\begin{aligned} \left( \sum _{\mathrm{j}=1}^{\mathrm{n}-1}\frac{\lambda _{\mathrm{j}}}{\gamma \lambda _{\mathrm{j}}+1}\right) ^{-1}\sum _{\mathrm{i}=1}^{\mathrm{n}-1}\left( \frac{\mathbf{y }'\mathbf{U }_{\mathrm{i}}}{\gamma \lambda _{\mathrm{i}}+1}\right) ^{2}\lambda _{\mathrm{i}} - \left( \sum _{\mathrm{j}=1}^{\mathrm{n}-1}\frac{1}{\gamma \lambda _{\mathrm{j}}+1}\right) ^{-1}\left[ \sum _{\mathrm{i}=1}^{\mathrm{n}-1}\left( \frac{\mathbf{y }'\mathbf{U }_{\mathrm{i}}}{\gamma \lambda _{\mathrm{i}}+1}\right) ^{2} - \hbox {n}\bar{\hbox {y}}^{2}\right] = 0, \end{aligned}$$

multiplying the identity by $\left[ \sum _{\mathrm{j}=1}^{\mathrm{n}-1}\lambda _{\mathrm{j}}/(\gamma \lambda _{\mathrm{j}}+1)\right] \left[ \sum _{\mathrm{j}=1}^{\mathrm{n}-1}1/(\gamma \lambda _{\mathrm{j}}+1)\right]$,

21 $$\begin{aligned} \sum _{\mathrm{i}=1}^{\mathrm{n}-1}\left( \frac{\mathbf{y }'\mathbf{U }_{\mathrm{i}}}{\gamma \lambda _{\mathrm{i}}+1}\right) ^{2}\lambda _{\mathrm{i}}\left( \sum _{\mathrm{j}=1}^{\mathrm{n}-1}\frac{1}{\gamma \lambda _{\mathrm{j}}+1}\right) - \left( \sum _{\mathrm{j}=1}^{\mathrm{n}-1}\frac{\lambda _{\mathrm{j}}}{\gamma \lambda _{\mathrm{j}}+1}\right) \left[ \sum _{\mathrm{i}=1}^{\mathrm{n}-1}\left( \frac{\mathbf{y }'\mathbf{U }_{\mathrm{i}}}{\gamma \lambda _{\mathrm{i}}+1}\right) ^{2} - \hbox {n}\bar{\hbox {y}}^{2}\right] = 0, \end{aligned}$$

rewriting with $\sum _{\mathrm{i}=1}^{\mathrm{n}-1}(\mathbf{y }'\mathbf{U }_{\mathrm{i}})^{2}/(\gamma \lambda _{\mathrm{i}}+1)^{2}$ in evidence,

22 $$\begin{aligned} \sum _{\mathrm{i}=1}^{\mathrm{n}-1}\left( \frac{\mathbf{y }'\mathbf{U }_{\mathrm{i}}}{\gamma \lambda _{\mathrm{i}}+1}\right) ^{2}\left[ \sum _{\mathrm{j}=1}^{\mathrm{n}-1}\frac{\lambda _{\mathrm{i}}}{\gamma \lambda _{\mathrm{j}}+1} - \sum _{\mathrm{j}=1}^{\mathrm{n}-1}\frac{\lambda _{\mathrm{j}}}{\gamma \lambda _{\mathrm{j}}+1}\right] + \hbox {n}\bar{\hbox {y}}^{2}\sum _{\mathrm{j}=1}^{\mathrm{n}-1}\frac{\lambda _{\mathrm{j}}}{\gamma \lambda _{\mathrm{j}}+1} = 0, \end{aligned}$$

and symplifying further,

23 $$\begin{aligned} \sum _{\mathrm{i}=1}^{\mathrm{n}-1}\left( \frac{\mathbf{y }'\mathbf{U }_{\mathrm{i}}}{\gamma \lambda _{\mathrm{i}}+1}\right) ^{2}\left[ \sum _{\mathrm{j}=1}^{\mathrm{n}-1}\frac{\lambda _{\mathrm{i}}-\lambda _{\mathrm{j}}}{\gamma \lambda _{\mathrm{j}}+1}\right] + \hbox {n}\bar{\hbox {y}}^{2}\sum _{\mathrm{j}=1}^{\mathrm{n}-1}\frac{\lambda _{\mathrm{j}}}{\gamma \lambda _{\mathrm{j}}+1} = 0. \end{aligned}$$

Finally, $\hat{\gamma }$ is the root of what we now refer to as REML function,

24 $$\begin{aligned} \hbox {g}(\gamma ) = \sum _{\mathrm{i}=1}^{\mathrm{n}-1}\sum _{\mathrm{j}=1}^{\mathrm{n}-1}\frac{(\mathbf{y }'\mathbf{U }_{\mathrm{i}})^{2}}{(1 + \gamma \lambda _{\mathrm{i}})^{2}}\left( \frac{\lambda _{\mathrm{i}} - \lambda _{\mathrm{j}}}{1 + \gamma \lambda _{\mathrm{j}}}\right) + \hbox {n}\bar{\hbox {y}}^{2}\sum _{\mathrm{j}=1}^{\mathrm{n}-1}\frac{\lambda _{\mathrm{j}}}{1 + \gamma \lambda _{\mathrm{j}}}. \end{aligned}$$

## Appendix 2: Correlations between phenotypes and eigen-vectors

As defined by the true model, the phenotypes are $\mathbf{y } = \mathbf{1 }_{\mathrm{n}}\mu + \mathbf{W }_{\mathrm{Q}}\mathbf{b }_{\mathrm{Q}} + \varvec{\varepsilon }_{\mathbf{Q}}$. Since $\varvec{\varepsilon }_{\mathbf{Q}}$ is assumed (and simulated) to be independent from all the other elements, $\varvec{\varepsilon }_{\mathbf{Q}}'\mathbf{U }_{\mathrm{i}} = \hbox {n}\hat{\mathbb {C}}\hbox {ov}(\varvec{\varepsilon }_{\mathbf{Q}},\mathbf{U }_{\mathrm{i}}) \rightarrow 0$ for $\hbox {n}$ sufficiently large. Thus,

25 $$\begin{aligned} \mathbf{y }'\mathbf{U }_{\mathrm{i}} = \mathbf{1 }_{\mathrm{n}}'\mathbf{U }_{\mathrm{i}}\mu + \mathbf{b }_{\mathrm{Q}}'\mathbf{W }_{\mathrm{Q}}'\mathbf{U }_{\mathrm{i}} + \varvec{\varepsilon }_{\mathbf{Q}}'\mathbf{U }_{\mathrm{i}} \longrightarrow \mathbf{1 }_{\mathrm{n}}'\mathbf{U }_{\mathrm{i}}\mu + \mathbf{b }_{\mathrm{Q}}'\mathbf{W }_{\mathrm{Q}}'\mathbf{U }_{\mathrm{i}}, \end{aligned}$$

and the squared term,

26 $$\begin{aligned} \left( \mathbf{y }'\mathbf{U }_{\mathrm{i}}\right) ^{2} \longrightarrow \left( \mathrm{n}\mu \bar{\mathrm{U}}_{\mathrm{i}}\right) ^{2} + 2\mathrm{n}\mu \bar{\mathrm{U}}_{\mathrm{i}}\mathbf{b }_{\mathrm{Q}}'\mathbf{W }_{\mathrm{Q}}'\mathbf{U }_{\mathrm{Qi}} + \left( \mathbf{b }_{\mathrm{Q}}'\mathbf{W }_{\mathrm{Q}}'\mathbf{U }_{\mathrm{i}}\right) ^{2}. \end{aligned}$$

Assuming the true model, $\mathbf{U }_{\mathrm{i}} = \mathbf{U }_{\mathrm{Qi}} \Longrightarrow \bar{\hbox {U}}_{\mathrm{Qi}} = 0$, $\forall$ $\lambda _{\mathrm{Qi}} > 0$ and $\mathbf{b }_{\mathrm{Q}}'\mathbf{W }_{\mathrm{Q}}'\mathbf{U }_{\mathrm{i}} = 0$, $\forall$ $\lambda _{\mathrm{Qi}} = 0$. Therefore, $2\hbox {n}\mu \bar{\hbox {U}}_{\mathrm{Qi}}\mathbf{b }_{\mathrm{Q}}'\mathbf{W }_{\mathrm{Q}}'\mathbf{U }_{\mathrm{Qi}} = 0$, $\forall$ $\hbox {i}=1,\ldots ,\hbox {n}$. Assuming the genomic model, we will assume that the number of SNPs is greater than the number of individuals, *i.e.* $\hbox {s} > \hbox {n}$, meaning that $\lambda _{1} \ge \cdots \ge \lambda _{\mathrm{n}-1} > \lambda _{\mathrm{n}} = 0$, $\bar{\hbox {U}}_{1} = \cdots = \bar{\hbox {U}}_{\mathrm{n}-1} = 0$, and $\mathbf{b }_{\mathrm{Q}}'\mathbf{W }_{\mathrm{Q}}'\mathbf{U }_{\mathrm{n}} = 0$. Therefore, $2\hbox {n}\mu \bar{\hbox {U}}_{\mathrm{Qi}}\mathbf{b }_{\mathrm{Q}}'\mathbf{W }_{\mathrm{Q}}'\mathbf{U }_{\mathrm{Qi}} = 0$, $\forall$ $\hbox {i}=1,\ldots ,\hbox {n}$. Finally,

27 $$\begin{aligned} \left( \mathbf{y }'\mathbf{U }_{\mathrm{i}}\right) ^{2} \longrightarrow \left( \hbox {n}\mu \bar{\hbox {U}}_{\mathrm{i}}\right) ^{2} + \left( \mathbf{b }_{\mathrm{Q}}'\mathbf{W }_{\mathrm{Q}}'\mathbf{U }_{\mathrm{i}}\right) ^{2}. \end{aligned}$$

## Appendix 3: Non-observable REML function

When we use the result from (27) into the REML function (24), we obtain the following:

28 $$\begin{aligned} \hbox {g}(\gamma ) = \sum _{\mathrm{i}=1}^{\mathrm{n}-1}\sum _{\mathrm{j}=1}^{\mathrm{n}-1}\left( \frac{\hbox {n}\mu \bar{\hbox {U}}_{\mathrm{i}}}{1 + \gamma \lambda _{\mathrm{i}}}\right) ^{2}\left( \frac{\lambda _{\mathrm{i}} - \lambda _{\mathrm{j}}}{1 + \gamma \lambda _{\mathrm{j}}}\right) + \sum _{\mathrm{i}=1}^{\mathrm{n}-1}\sum _{\mathrm{j}=1}^{\mathrm{n}-1}\frac{\left( \mathbf{b }_{\mathrm{Q}}'\mathbf{W }_{\mathrm{Q}}'\mathbf{U }_{\mathrm{i}}\right) ^{2}}{(1 + \gamma \lambda _{i})^{2}}\left( \frac{\lambda _{\mathrm{i}} - \lambda _{\mathrm{j}}}{1 + \gamma \lambda _{\mathrm{j}}}\right) + \hbox {n}\bar{\mathrm{y}}^{2}\sum _{\mathrm{j}=1}^{\mathrm{n}-1}\frac{\lambda _{\mathrm{j}}}{1 + \gamma \lambda _{\mathrm{j}}}, \end{aligned}$$

in which we rewrite the elements of the first term as

29 $$\begin{aligned} \left( \frac{\hbox {n}\mu \bar{\hbox {U}}_{\mathrm{i}}}{1 + \gamma \lambda _{\mathrm{i}}}\right) ^{2}\left( \frac{\lambda _{\mathrm{i}} - \lambda _{\mathrm{j}}}{1 + \gamma \lambda _{\mathrm{j}}}\right) = \hbox {n}^{2}\mu ^{2}\left[ \left( \frac{\bar{\hbox {U}}_{\mathrm{i}}}{1 + \gamma \lambda _{\mathrm{i}}}\right) ^{2}\frac{\lambda _{\mathrm{i}}}{1 + \gamma \lambda _{\mathrm{j}}} - \left( \frac{\bar{\hbox {U}}_{\mathrm{i}}}{1 + \gamma \lambda _{\mathrm{i}}}\right) ^{2}\frac{\lambda _{\mathrm{j}}}{1 + \gamma \lambda _{\mathrm{j}}}\right] . \end{aligned}$$

Now, since $\bar{\hbox {U}}_{\mathrm{i}} = 0$, $\forall$ $\lambda _{\mathrm{i}} > 0 \Longrightarrow (\bar{\hbox {U}}_{\mathrm{i}})^{2}\lambda _{\mathrm{i}} = 0$, $\forall$ $\hbox {i}=1,\ldots ,\hbox {n}$. Thus,

30 $$\begin{aligned} \left( \frac{\hbox {n}\mu \bar{\hbox {U}}_{\mathrm{i}}}{1 + \gamma \lambda _{\mathrm{i}}}\right) ^{2}\left( \frac{\lambda _{\mathrm{i}} - \lambda _{\mathrm{j}}}{1 + \gamma \lambda _{\mathrm{j}}}\right) = -\hbox {n}^{2}\mu ^{2}\left( \frac{\bar{\hbox {U}}_{\mathrm{i}}}{1 + \gamma \lambda _{\mathrm{i}}}\right) ^{2}\frac{\lambda _{\mathrm{j}}}{1 + \gamma \lambda _{\mathrm{j}}}. \end{aligned}$$

Because $\bar{\hbox {U}}_{\mathrm{i}} \ne 0$ only when $\lambda _{\hbox {i}} = 0$ and $\sum _{\mathrm{i}=1}^{\mathrm{n}}\bar{\hbox {U}}_{\mathrm{i}}^{2} = \hbox {n}^{-1}$,

31 $$\begin{aligned} \sum _{\mathrm{i}=1}^{\mathrm{n}-1}\sum _{\mathrm{j}=1}^{\mathrm{n}-1}\left( \frac{\hbox {n}\mu \bar{\hbox {U}}_{\mathrm{i}}}{1 + \gamma \lambda _{\mathrm{i}}}\right) ^{2}\left( \frac{\lambda _{\mathrm{i}} - \lambda _{\mathrm{j}}}{1 + \gamma \lambda _{\mathrm{j}}}\right) = -\hbox {n}\mu ^{2}\sum _{\mathrm{j}=1}^{\mathrm{n}-1}\frac{\lambda _{\mathrm{j}}}{1 + \gamma \lambda _{\mathrm{j}}}. \end{aligned}$$

When $\hbox {n}$ is sufficiently large, $\bar{\hbox {y}} \longrightarrow \mu$, and $\hbox {n}\bar{\hbox {y}}^{2}\sum _{\mathrm{j}=1}^{\mathrm{n}-1}\lambda _{\mathrm{j}}/(1 + \gamma \lambda _{\mathrm{j}})-\hbox {n}\mu ^{2}\sum _{\mathrm{j}=1}^{\mathrm{n}-1}\lambda _{\mathrm{j}}/(1 + \gamma \lambda _{\mathrm{j}})\longrightarrow 0$. Therefore:

32 $$\begin{aligned} \hbox {g}(\gamma ) \longrightarrow \sum _{\mathrm{i}=1}^{\mathrm{n}-1}\sum _{\mathrm{j}=1}^{\mathrm{n}-1}\frac{\left( \mathbf{b }_{\mathrm{Q}}'\mathbf{W }_{\mathrm{Q}}'\mathbf{U }_{\mathrm{i}}\right) ^{2}}{(1 + \gamma \lambda _{\mathrm{i}})^{2}}\left( \frac{\lambda _{\mathrm{i}} - \lambda _{\mathrm{j}}}{1 + \gamma \lambda _{\mathrm{j}}}\right) . \end{aligned}$$

We now move to understand some properties of $\mathbf{b }_{\mathrm{Q}}'\mathbf{W }_{\mathrm{Q}}'\mathbf{U }_{\mathrm{i}}$, keeping in mind that in the assumptions (and simulations) that we make in our study the QTL are independent from each other, and the QTL effects are independent from all the other elements. Another important assumption used here is that $\mathbf{W }_{\mathrm{Q1}}'\mathbf{U }_{\mathrm{i}},\ldots ,\mathbf{W }_{\mathrm{Qq}}'\mathbf{U }_{\mathrm{i}}$ are $\hbox {iid}$, which allows us to define

1. $$\mathbb {E}\left( \mathbf{b }_{\mathrm{Q}}'\mathbf{W }_{\mathrm{Q}}'\mathbf{U }_{\mathrm{i}}\right) = 0$$,
2. $$\mathbb {V}\hbox {ar}\left( \mathbf{b }_{\mathrm{Q}}'\mathbf{W }_{\mathrm{Q}}'\mathbf{U }_{\mathrm{i}}\right) = \sigma _{\mathrm{T}_{\mathrm{Q}}}^{2}\mathbb {V}\hbox {ar}\left( \mathbf{W }_{\mathrm{Qj}}'\mathbf{U }_{\mathrm{i}}\right) = \mathbb {E}\left( \left[ \mathbf{b }_{\mathrm{Q}}'\mathbf{W }_{\mathrm{Q}}'\mathbf{U }_{\mathrm{i}}\right] ^{2}\right)$$.

Thus, we have $\left( \mathbf{b }_{\mathrm{Q}}'\mathbf{W }_{\mathrm{Q}}'\mathbf{U }_{\mathrm{i}}\right) ^{2}$ as an estimator,

33 $$\begin{aligned} \left( \mathbf{b }_{\mathrm{Q}}'\mathbf{W }_{\mathrm{Q}}'\mathbf{U }_{\mathrm{i}}\right) ^{2} = \hat{\mathbb {E}}\left( \left[ \mathbf{b }_{\mathrm{Q}}'\mathbf{W }_{\mathrm{Q}}'\mathbf{U }_{\mathrm{i}}\right] ^{2}\right) = \hat{\mathbb {V}}\hbox {ar}\left( \mathbf{b }_{\mathrm{Q}}'\mathbf{W }_{\mathrm{Q}}'\mathbf{U }_{\mathrm{i}}\right) = \hat{\sigma }_{\mathrm{T}_{\mathrm{Q}}}^{2}\hat{\mathbb {V}}\hbox {ar}\left( \mathbf{W }_{\mathrm{Qj}}'\mathbf{U }_{\mathrm{i}}\right) . \end{aligned}$$

And finally, the non-observable REML function:

34 $$\begin{aligned} \hbox {g}(\gamma ) \longrightarrow \hat{\sigma }_{\mathrm{T}_{\mathrm{Q}}}^{2}\sum _{\mathrm{i}=1}^{\mathrm{n}-1}\sum _{\mathrm{j}=1}^{\mathrm{n}-1}\frac{\hat{\mathbb {V}}\hbox {ar}\left( \mathbf{W }_{\mathrm{Qj}}'\mathbf{U }_{\mathrm{i}}\right) }{(1 + \gamma \lambda _{\mathrm{i}})^{2}}\left( \frac{\lambda _{\mathrm{i}} - \lambda _{\mathrm{j}}}{1 + \gamma \lambda _{\mathrm{j}}}\right) . \end{aligned}$$

Replacing $\hat{\mathbb {V}}\hbox {ar}\left( \mathbf{W }_{\mathrm{Qj}}'\mathbf{U }_{\mathrm{i}}\right)$ with an estimator $\hat{\mathbb {V}}\hbox {ar}\left( \mathbf{W }_{\mathrm{Qj}}'\mathbf{U }_{\mathrm{i}}\right) = \hbox {q}^{-1}\mathbf{U }_{\mathrm{i}}'\mathbf{W }_{\mathrm{Q}}\mathbf{W }_{\mathrm{Q}}'\mathbf{U }_{\mathrm{i}} - \hbox {q}^{-2}(\mathbf{1 }_{\mathrm{q}}'\mathbf{W }_{\mathrm{Q}}'\mathbf{U }_{\mathrm{i}})^{2} = \mathbf{U }_{\mathrm{i}}'\mathbf{U }_{\mathrm{Q}}\varvec{\Lambda }_{\mathrm{Q}}\mathbf{U }_{\mathrm{Q}}'\mathbf{U }_{\mathrm{i}} - \hbox {q}^{-2}(\mathbf{1 }_{\mathrm{q}}'\mathbf{W }_{\mathrm{Q}}'\mathbf{U }_{\mathrm{i}})^{2}$,

35 $$\begin{aligned} \hbox {g}(\gamma ) \longrightarrow \hat{\sigma }_{\mathrm{T}_{\mathrm{Q}}}^{2}\sum _{\mathrm{i}=1}^{\mathrm{n}-1}\sum _{\mathrm{j}=1}^{\mathrm{n}-1}\frac{\left[ \mathbf{U }_{\mathrm{i}}'\mathbf{U }_{\mathrm{Q}}\varvec{\Lambda }_{\mathrm{Q}}\mathbf{U }_{\mathrm{Q}}'\mathbf{U }_{\mathrm{i}} - \hbox {q}^{-2}(\mathbf{1 }_{\mathrm{q}}'\mathbf{W }_{\mathrm{Q}}'\mathbf{U }_{\mathrm{i}})^{2}\right] }{(1 + \gamma \lambda _{\mathrm{i}})^{2}}\left( \frac{\lambda _{\mathrm{i}} - \lambda _{\mathrm{j}}}{1 + \gamma \lambda _{\mathrm{j}}}\right) , \end{aligned}$$

and for $\hbox {n}$ sufficiently large, $\hbox {q}^{-2}(\mathbf{1 }_{\mathrm{q}}'\mathbf{W }_{\mathrm{Q}}'\mathbf{U }_{\mathrm{i}})^{2}<< < \mathbf{U }_{\mathrm{i}}'\mathbf{U }_{\mathrm{Q}}\varvec{\Lambda }_{\mathrm{Q}}\mathbf{U }_{\mathrm{Q}}'\mathbf{U }_{\mathrm{i}} = \sum _{\mathrm{k}=1}^{\mathrm{n}}(\mathbf{U }_ {\mathrm{i}}'\mathbf{U }_{\mathrm{Qk}})^{2}\lambda _{\mathrm{Qk}}$, with $\sum _{\mathrm{k}=1}^{\mathrm{n}}(\mathbf{U }_ {\mathrm{n}}'\mathbf{U }_{\mathrm{Qk}})^{2}\lambda _{\mathrm{Qk}} = 0$ due to properties of the eigen-decomposition. So we can approximate the non-observable REML function,

36 $$\begin{aligned} \hbox {g}(\gamma ) \longrightarrow \hat{\sigma }_{\mathrm{T}_{\mathrm{Q}}}^{2}\sum _{\mathrm{i}=1}^{\mathrm{n}-1}\sum _{\mathrm{j}=1}^{\mathrm{n}-1}\frac{\sum _{\mathrm{k}=1}^{\mathrm{n}}(\mathbf{U }_ {\mathrm{i}}'\mathbf{U }_{\mathrm{Qk}})^{2}\lambda _{\mathrm{Qk}}}{(1 + \gamma \lambda _{\mathrm{i}})^{2}}\left( \frac{\lambda _{\mathrm{i}} - \lambda _{\mathrm{j}}}{1 + \gamma \lambda _{\mathrm{j}}}\right) , \end{aligned}$$

and since our interest in evaluating $g(\hat{\gamma }) = 0$, it is equivalent to evaluate $\hat{\sigma }_{\mathrm{T}_{\mathrm{Q}}}^{-2}\hbox {g}(\hat{\gamma }) = 0$, thus:

37 $$\begin{aligned} \sum _{\mathrm{i}=1}^{\mathrm{n}-1}\sum _{\mathrm{j}=1}^{\mathrm{n}-1}\frac{\sum _{\mathrm{k}=1}^{\mathrm{n}}(\mathbf{U }_ {\mathrm{i}}'\mathbf{U }_{\mathrm{Qk}})^{2}\lambda _{\mathrm{Qk}}}{(1 + \gamma \lambda _{\mathrm{i}})^{2}}\left( \frac{\lambda _{\mathrm{i}} - \lambda _{\mathrm{j}}}{1 + \gamma \lambda _{\mathrm{j}}}\right) = 0. \end{aligned}$$

We call this function non-observable because we have now written it as a function of $\mathbf{U }_{\mathrm{Q}}$ and $\varvec{\Lambda }_{\mathrm{Q}}$, that cannot be observed directly from phenotypes and genomic data.

## Appendix 4: Summations as deviations from the A-matrix

Let $\mathbf{G }_{\mathrm{1}} = \mathbf{U }_{\mathrm{1}}\varvec{\Lambda }_{1}\mathbf{U }_{\mathrm{1}} = \mathbf{A } + \varvec{\Delta }_{1}$ and $\mathbf{G }_{\mathrm{2}} = \mathbf{U }_{\mathrm{2}}\varvec{\Lambda }_{2}\mathbf{U }_{\mathrm{2}} = \mathbf{A } + \varvec{\Delta }_{2}$ be two G-matrices based on any set of SNPs, such that $\varvec{\Delta }_{1}$ and $\varvec{\Delta }_{2}$ are how much $\mathbf{G }_{\mathrm{1}}$ and $\mathbf{G }_{\mathrm{2}}$ respectively, deviate from $\mathbf{A }$, the matrix of expected relationships between the individuals, expressed as correlations. We have then the following:

38 $$\begin{aligned} \sum _{\mathrm{k}=1}^{\mathrm{n}}\left( \mathbf{U }_{\mathrm{1i}}'\mathbf{U }_{\mathrm{2k}}\right) ^{2}\lambda _{2\mathrm{k}}= & \sum _{\mathrm{j}=1}^{\mathrm{n}}\hbox {U}_{\mathrm{1ij}}^{2}\sum _{\mathrm{k}=1}^{\mathrm{n}}\hbox {U}_{\mathrm{2kj}}^{2}\lambda _{\mathrm{2k}} + \sum _{\mathrm{j}=1}^{\mathrm{n}}\sum _{\mathrm{l}\ne \mathrm{j}}\hbox {U}_{\mathrm{1ij}}\hbox {U}_{\mathrm{1il}}\sum _{\mathrm{k}=1}^{\mathrm{n}}\hbox {U}_{\mathrm{2kj}}\hbox {U}_{\mathrm{2kl}}\lambda _{\mathrm{2k}} \nonumber \\= & \sum _{\mathrm{j}=1}^{\mathrm{n}}\hbox {U}_{\mathrm{1ij}}^{2}\hbox {G}_{\mathrm{2jj}} + \sum _{\mathrm{j}=1}^{\mathrm{n}}\sum _{\mathrm{l}\ne \mathrm{j}}\hbox {U}_{\mathrm{1ij}}\hbox {U}_{\mathrm{1il}}\hbox {G}_{\mathrm{2jl}} \nonumber \\= & \sum _{\mathrm{j}=1}^{\mathrm{n}}\hbox {U}_{\mathrm{1ij}}^{2}\left( \hbox {a}_{\mathrm{jj}} + \delta _{\mathrm{2jj}}\right) + \sum _{\mathrm{j}=1}^{\mathrm{n}}\sum _{\mathrm{l}\ne \mathrm{j}}\hbox {U}_{\mathrm{1ij}}\hbox {U}_{\mathrm{1il}}\left( \hbox {a}_{\mathrm{jl}} + \delta _{\mathrm{2jl}}\right) \nonumber \\= & \sum _{\mathrm{j}=1}^{\mathrm{n}}\hbox {U}_{\mathrm{1ij}}^{2}\left( 1 + \delta _{\mathrm{2jj}}\right) + \sum _{\mathrm{j}=1}^{\mathrm{n}}\sum _{\mathrm{l}\ne \mathrm{j}}\hbox {U}_{\mathrm{1ij}}\hbox {U}_{\mathrm{1il}}\left( \hbox {a}_{\mathrm{jl}} + \delta _{\mathrm{2jl}}\right) \nonumber \\= & \sum _{\mathrm{j}=1}^{\mathrm{n}}\hbox {U}_{\mathrm{1ij}}^{2} + \sum _{\mathrm{j}=1}^{\mathrm{n}}\hbox {U}_{\mathrm{1ij}}^{2}\delta _{\mathrm{2jj}} + \sum _{\mathrm{j}=1}^{\mathrm{n}}\sum _{\mathrm{l}\ne \mathrm{j}}\hbox {U}_{\mathrm{1ij}}\hbox {U}_{\mathrm{1il}}\left( \hbox {a}_{\mathrm{jl}} + \delta _{\mathrm{2jl}}\right) \nonumber \\= & 1 + \sum _{\mathrm{j}=1}^{\mathrm{n}}\hbox {U}_{\mathrm{1ij}}^{2}\delta _{\mathrm{2jj}} + \sum _{\mathrm{j}=1}^{\mathrm{n}}\sum _{\mathrm{l}\ne \mathrm{j}}\hbox {U}_{\mathrm{1ij}}\hbox {U}_{\mathrm{1il}}\left( \hbox {a}_{\mathrm{jl}} + \delta _{\mathrm{2jl}}\right) , \end{aligned}$$

and

39 $$\begin{aligned} \sum _{\mathrm{k}=1}^{\mathrm{n}}\mathbf{U }_{\mathrm{1i}}'\mathbf{U }_{\mathrm{2k}}\mathbf{U }_{\mathrm{2k}}'\mathbf{U }_{\mathrm{1r}}\lambda _{\mathrm{2k}}= & \sum _{\mathrm{j}=1}^{\mathrm{n}}\hbox {U}_{\mathrm{1ij}}\hbox {U}_{\mathrm{1rj}}\sum _{\mathrm{k}=1}^{\mathrm{n}}\hbox {U}_{\mathrm{2kj}}^{2}\lambda _{\mathrm{2k}} + \sum _{\mathrm{j}=1}^{\mathrm{n}}\sum _{\mathrm{l}\ne \mathrm{j}}\hbox {U}_{\mathrm{1ij}}\hbox {U}_{\mathrm{1rl}}\sum _{\mathrm{k}=1}^{\mathrm{n}}\hbox {U}_{\mathrm{2kj}}\hbox {U}_{\mathrm{2kl}}\lambda _{\mathrm{2k}} \nonumber \\= & \sum _{\mathrm{j}=1}^{\mathrm{n}}\mathrm{U}_{\mathrm{1ij}}\mathrm{U}_{\mathrm{1rj}}\mathrm{G}_{\mathrm{2jj}} + \sum _{\mathrm{j}=1}^{\mathrm{n}}\sum _{\mathrm{l}\ne \mathrm{j}}\mathrm{U}_{\mathrm{1ij}}\hbox {U}_{\mathrm{1rl}}\hbox {G}_{\mathrm{2jl}} \nonumber \\= & \sum _{\mathrm{j}=1}^{\mathrm{n}}\hbox {U}_{\mathrm{1ij}}\hbox {U}_{\mathrm{1rj}}\left( \hbox {a}_{\mathrm{jj}} + \delta _{\mathrm{2jj}}\right) + \sum _{\mathrm{j}=1}^{\mathrm{n}}\sum _{\mathrm{l}\ne \mathrm{j}}\hbox {U}_{\mathrm{1ij}}\hbox {U}_{\mathrm{1rl}}\left( \hbox {a}_{\mathrm{jl}} + \delta _{\mathrm{2jl}}\right) \nonumber \\= & \sum _{\mathrm{j}=1}^{\mathrm{n}}\mathrm{U}_{\mathrm{1ij}}\mathrm{U}_{\mathrm{1rj}}\left( 1 + \delta _{\mathrm{2jj}}\right) + \sum _{\mathrm{j}=1}^{\mathrm{n}}\sum _{\mathrm{l}\ne \mathrm{j}}\mathrm{U}_{\mathrm{1ij}}\mathrm{U}_{\mathrm{1rl}}\left( \mathrm{a}_{\mathrm{jl}} + \delta _{\mathrm{2jl}}\right) \nonumber \\= & \sum _{\mathrm{j}=1}^{\mathrm{n}}\mathrm{U}_{\mathrm{1ij}}\mathrm{U}_{\mathrm{1rj}} + \sum _{\mathrm{j}=1}^{\mathrm{n}}\mathrm{U}_{\mathrm{1ij}}\hbox {U}_{\mathrm{1rj}}\delta _{\mathrm{2jj}} + \sum _{\mathrm{j}=1}^{\mathrm{n}}\sum _{\mathrm{l}\ne \mathrm{j}}\hbox {U}_{\mathrm{1ij}}\hbox {U}_{\mathrm{1rl}}\left( \hbox {a}_{\mathrm{jl}} + \delta _{\mathrm{2jl}}\right) \nonumber \\= & \sum _{\mathrm{j}=1}^{\mathrm{n}}\hbox {U}_{\mathrm{1ij}}\hbox {U}_{\mathrm{1rj}}\delta _{\mathrm{2jj}} + \sum _{\mathrm{j}=1}^{\mathrm{n}}\sum _{\mathrm{l}\ne \mathrm{j}}\hbox {U}_{\mathrm{1ij}}\hbox {U}_{\mathrm{1rl}}\left( \hbox {a}_{\mathrm{jl}} + \delta _{\mathrm{2jl}}\right) , \end{aligned}$$

such that $\hbox {a}_{\mathrm{jl}} \in [0,0.5]$ is the expected relationship between the $\hbox {j-th}$ and $\hbox {l-th}$ individuals (in a non-inbred population), $\hbox {a}_{\mathrm{jj}} = 1$ (the expected relationship of and individual to itself, in a non-inbred population). Because of the way we defined the G-matrices, and $\mathbf{G }_{\mathrm{1}},\mathbf{G }_{\mathrm{2}} \rightarrow \mathbf{A }$ when the number of SNPs is sufficiently large [17], it is intuitive that $\mathbb {E}\left( \delta _{*\mathrm{jj}}\right) = \mathbb {E}\left( \delta _{*\mathrm{jl}}\right) = 0$, with $\mathbb {V}\hbox {ar}\left( \delta _{*\mathrm{jj}}\right)$ and $\mathbb {V}\hbox {ar}\left( \delta _{*\mathrm{jl}}\right)$ proportionally inverse to the number of SNPs.

## Appendix 5: QTL and markers in complete linkage equilibrium

### E.1 QTL + markers

We will use the following identities relating $\mathbf{G }_{\mathrm{QM}}$, $\mathbf{G }_{\mathrm{Q}}$ and $\mathbf{G }_{\mathrm{M}}$ to understand the properties of the REML estimators for the scenarios we simulated. Using the eigen-decompositions, we have:

40 $$\begin{aligned} \mathbf{G }_{\mathrm{QM}} = \frac{\hbox {q}}{\hbox {q}+\hbox {m}}\mathbf{G }_{\mathrm{Q}} + \frac{\hbox {m}}{\hbox {q}+\hbox {m}}\mathbf{G }_{\mathrm{M}} = \mathbf{U }_{\mathrm{Q}}\left( \frac{\hbox {q}}{\hbox {q}+\hbox {m}}\varvec{\Lambda }_{\mathrm{Q}} + \frac{\hbox {m}}{\hbox {q}+\hbox {m}}\mathbf{U }_{\mathrm{Q}}'\mathbf{U }_{\mathrm{M}}\varvec{\Lambda }_{\mathrm{M}}\mathbf{U }_{\mathrm{M}}'\mathbf{U }_{\mathrm{Q}}\right) \mathbf{U }_{\mathrm{Q}}'. \end{aligned}$$

Now, because $\mathbf{G }_{\mathrm{QM}} = \mathbf{U }_{\mathrm{QM}}\varvec{\Lambda }_{\mathrm{QM}}\mathbf{U }_{\mathrm{QM}}'$, using that identity in Eq. (40) we have:

41 $$\begin{aligned} \mathbf{U }_{\mathrm{QM}}\varvec{\Lambda }_{\mathrm{QM}}\mathbf{U }_{\mathrm{QM}}' = \mathbf{U }_{\mathrm{Q}}\left( \frac{\hbox {q}}{\hbox {q}+\hbox {m}}\varvec{\Lambda }_{\mathrm{Q}} + \frac{\hbox {m}}{\hbox {q}+\hbox {m}}\mathbf{U }_{\mathrm{Q}}'\mathbf{U }_{\mathrm{M}}\varvec{\Lambda }_{\mathrm{M}}\mathbf{U }_{\mathrm{M}}'\mathbf{U }_{\mathrm{Q}}\right) \mathbf{U }_{\mathrm{Q}}', \end{aligned}$$

hence,

42 $$\begin{aligned} \varvec{\Lambda }_{\mathrm{QM}} = \mathbf{U }_{\mathrm{QM}}'\mathbf{U }_{\mathrm{Q}}\left( \frac{\hbox {q}}{\hbox {q}+\hbox {m}}\varvec{\Lambda }_{\mathrm{Q}} + \frac{\hbox {m}}{\hbox {q}+\hbox {m}}\mathbf{U }_{\mathrm{Q}}'\mathbf{U }_{\mathrm{M}}\varvec{\Lambda }_{\mathrm{M}}\mathbf{U }_{\mathrm{M}}'\mathbf{U }_{\mathrm{Q}}\right) \times\,\mathbf{U }_{\mathrm{Q}}'\mathbf{U }_{\mathrm{QM}}. \end{aligned}$$

We must now understand what happens to the term $\mathbf{U }_{\mathrm{Q}}'\mathbf{U }_{\mathrm{M}}\varvec{\Lambda }_{\mathrm{M}}\mathbf{U }_{\mathrm{M}}'\mathbf{U }_{\mathrm{Q}}$ in Eq. (42), in order to understand what will happen the REML estimator of heritability. Applying the results from Eqs. (38) and (39), and because in these scenarios we only considered completely unrelated individuals (implying that $\hbox {a}_{\mathrm{jl}} = 0$), we have that the elements in the diagonal and off-diagonal of $\mathbf{U }_{\mathrm{Q}}'\mathbf{U }_{\mathrm{M}}\varvec{\Lambda }_{\mathrm{M}}\mathbf{U }_{\mathrm{M}}'\mathbf{U }_{\mathrm{Q}}$ are respectively,

43 $$\begin{aligned} \sum _{\mathrm{k}=1}^{\mathrm{n}}\left( \mathbf{U }_{\mathrm{Qi}}'\mathbf{U }_{\mathrm{Mk}}\right) ^{2}\lambda _{\mathrm{Mk}} = 1 + \sum _{\mathrm{j}=1}^{\mathrm{n}}\hbox {U}_{\mathrm{Qij}}^{2}\delta _{\mathrm{Mjj}} + \sum _{\mathrm{j}=1}^{\mathrm{n}}\sum _{\mathrm{l}\ne \mathrm{j}}\hbox {U}_{\mathrm{Qij}}\hbox {U}_{\mathrm{Qil}}\delta _{\mathrm{Mjl}}, \end{aligned}$$

and

44 $$\begin{aligned} \sum _{\mathrm{k}=1}^{\mathrm{n}}\mathbf{U }_{\mathrm{Qi}}'\mathbf{U }_{\mathrm{Mk}}\mathbf{U }_{\mathrm{Mk}}'\mathbf{U }_{\mathrm{Qr}}\lambda _{\mathrm{Mk}} = \sum _{\mathrm{j}=1}^{\mathrm{n}}\hbox {U}_{\mathrm{Qij}}\hbox {U}_{\mathrm{Qrj}}\delta _{\mathrm{Mjj}} + \sum _{\mathrm{j}=1}^{\mathrm{n}}\sum _{\mathrm{l}\ne \mathrm{j}}\hbox {U}_{\mathrm{Qij}}\hbox {U}_{\mathrm{Qrl}}\delta _{\mathrm{Mjl}}. \end{aligned}$$

Because the markers are in complete LE with the QTL, and remembering that $\mathbb {E}\left( \delta _{\mathrm{Mjj}}\right) = \mathbb {E}\left( \delta _{\mathrm{Mjl}}\right) = 0$, we have:

45 $$\begin{aligned} \mathbb {E}\left( \sum _{\mathrm{k}=1}^{\mathrm{n}}\left[ \mathbf{U }_{\mathrm{Qi}}'\mathbf{U }_{\mathrm{Mk}}\right] ^{2}\lambda _{\mathrm{Mk}}\right) = 1 + \sum _{\mathrm{j}=1}^{n}\mathbb {E}\left( \hbox {U}_{\mathrm{Qij}}^{2}\delta _{\mathrm{Mjj}}\right) + \sum _{\mathrm{j}=1}^{\mathrm{n}}\sum _{\mathrm{l}\ne \mathrm{j}}\mathbb {E}\left( \hbox {U}_{\mathrm{Qij}}\hbox {U}_{\mathrm{Qil}}\delta _{\mathrm{Mjl}}\right) = 1, \end{aligned}$$

and

46 $$\begin{aligned} \mathbb {E}\left( \sum _{\mathrm{k}=1}^{\mathrm{n}}\mathbf{U }_{\mathrm{Qi}}'\mathbf{U }_{\mathrm{Mk}}\mathbf{U }_{\mathrm{Mk}}'\mathbf{U }_{\mathrm{Qr}}\lambda _{\mathrm{Mk}}\right) = \sum _{\mathrm{j}=1}^{\mathrm{n}}\mathbb {E}\left( \mathrm{U}_{\mathrm{Qij}}\hbox {U}_{\mathrm{Qrj}}\delta _{\mathrm{Mjj}}\right) + \sum _{\mathrm{j}=1}^{\mathrm{n}}\sum _{\mathrm{l}\ne \mathrm{j}}\mathbb {E}\left( \hbox {U}_{\mathrm{Qij}}\hbox {U}_{\mathrm{Qrl}}\delta _{\mathrm{Mjl}}\right) = 0. \end{aligned}$$

Therefore, $\mathbb {E}\left( \mathbf{U }_{\mathrm{Q}}'\mathbf{U }_{\mathrm{M}}\varvec{\Lambda }_{\mathrm{M}}\mathbf{U }_{\mathrm{M}}'\mathbf{U }_{\mathrm{Q}}\right) = \mathbf{I }_{\mathrm{n}}$, meaning that we can write $\mathbf{U }_{\mathrm{Q}}'\mathbf{U }_{\mathrm{M}}\varvec{\Lambda }_{\mathrm{M}}\mathbf{U }_{\mathrm{M}}'\mathbf{U }_{\mathrm{Q}} = \mathbf{I }_{\mathrm{n}} + \varvec{\delta }$, such that $\varvec{\delta }$ is a matrix of random errors around $\mathbf{I }_{\mathrm{n}}$, with $\mathbb {E}\left( \varvec{\delta }\right) = \mathbf{0 }$. Back to Eq. (42), we have now:

47 $$\begin{aligned} \varvec{\Lambda }_{\mathrm{QM}} = \mathbf{U }_{\mathrm{QM}}'\mathbf{U }_{\mathrm{Q}}\left[ \frac{\hbox {q}}{\hbox {q}+\hbox {m}}\varvec{\Lambda }_{\mathrm{Q}} + \frac{\hbox {m}}{\hbox {q}+\hbox {m}}\left( \mathbf{I }_{\mathrm{n}} + \varvec{\delta }\right) \right] \mathbf{U }_{\mathrm{Q}}'\mathbf{U }_{\mathrm{QM}}, \end{aligned}$$

Therefore the elements in the diagonal of $\varvec{\Lambda }_{\mathrm{QM}}$ obey the following identity,

48 $$\begin{aligned} \lambda _{\mathrm{QMi}} = \frac{\hbox {q}}{\hbox {q}+\hbox {m}}\sum _{\mathrm{k}=1}^{\mathrm{n}}\left( \mathbf{U }_{\mathrm{QMi}}'\mathbf{U }_{\mathrm{Qk}}\right) ^{2}\lambda _{\mathrm{Qk}} + \frac{\hbox {m}}{\hbox {q}+\hbox {m}}\left( 1 + \delta _{\mathrm{i}}\right) , \end{aligned}$$

with $\delta {\hbox {i}} = \sum _{\mathrm{j}=1}^{\mathrm{n}}\mathrm{U}_{\mathrm{Qij}}^{2}\delta _{\mathrm{Mjj}} + \sum _{\mathrm{j}=1}^{\mathrm{n}}\sum _{\mathrm{l}\ne \mathrm{j}}\hbox {U}_{\mathrm{Qij}}\hbox {U}_{\mathrm{Qil}}\delta _{\mathrm{Mjl}}$, from Eq. (43), such that $\mathbb {E}\left( \delta _{\mathrm{i}}\right) = 0$, from Eq. (45). Finally, isolating $\sum _{\mathrm{k}=1}^{\mathrm{n}}\left( \mathbf{U }_{\mathrm{QMi}}'\mathbf{U }_{\mathrm{Qk}}\right) ^{2}\lambda _{\mathrm{Qk}}$ in Eq. (48):

49 $$\begin{aligned} \sum _{\mathrm{k}=1}^{\mathrm{n}}\left( \mathbf{U }_{\mathrm{QMi}}'\mathbf{U }_{\mathrm{Qk}}\right) ^{2}\lambda _{\mathrm{Qk}} = \lambda _{\mathrm{QMi}} + \frac{\hbox {m}}{\hbox {q}}\left( \lambda _{\mathrm{QMi}} - 1 - \delta _{\mathrm{i}}\right) . \end{aligned}$$

### E.2 Markers only

The genomic model containing the markers only is simpler than the genomic model containing the QTL and markers. Applying the result from Eq. (38), and because in these scenarios we only considered completely unrelated individuals (implying that $\hbox {a}_{\mathrm{jl}} = 0$), we have:

50 $$\begin{aligned} \sum _{\hbox {k}=1}^{\mathrm{n}}\left( \mathbf{U }_{\mathrm{Mi}}'\mathbf{U }_{\mathrm{Qk}}\right) ^{2}\lambda _{\mathrm{Qk}} = 1 + \sum _{\mathrm{j}=1}^{\mathrm{n}}\mathrm{U}_{\mathrm{Mij}}^{2}\delta _{\mathrm{Qjj}} + \sum _{\mathrm{j}=1}^{\mathrm{n}}\sum _{\mathrm{l}\ne \mathrm{j}}\mathrm{U}_{\mathrm{Mij}}\mathrm{U}_{\mathrm{Mil}}\delta _{\mathrm{Qjl}} = 1 + \delta _{\mathrm{i}}, \end{aligned}$$

with $\delta _{\mathrm{i}} = \sum _{\mathrm{j}=1}^{\mathrm{n}}\hbox {U}_{\mathrm{Mij}}^{2}\delta _{\mathrm{Qjj}} + \sum _{\mathrm{j}=1}^{\mathrm{n}}\sum _{\mathrm{l}\ne \mathrm{j}}\hbox {U}_{\mathrm{Mij}}\hbox {U}_{\mathrm{Mil}}\delta _{\mathrm{Qjl}}$. Because the markers are in complete LE with the QTL, and remembering that $\mathbb {E}\left( \delta _{\mathrm{Qjj}}\right) = \mathbb {E}\left( \delta _{\mathrm{Qjl}}\right) = 0$, we have:

51 $$\begin{aligned} \mathbb {E}\left( \delta _{\mathrm{i}}\right) = \sum _{\mathrm{j}=1}^{\mathrm{n}}\mathbb {E}\left( \hbox {U}_{\mathrm{Mij}}^{2}\delta _{\mathrm{Qjj}}\right) + \sum _{\mathrm{j}=1}^{\mathrm{n}}\sum _{\mathrm{l}\ne \mathrm{j}}\mathbb {E}\left( \hbox {U}_{\mathrm{Mij}}\hbox {U}_{\mathrm{Mil}}\delta _{\mathrm{Qjl}}\right) = 0. \end{aligned}$$

## Appendix 6: QTL and markers in linkage disequilibrium

### F.1 QTL + markers

We will use the following identities relating $\mathbf{G }_{\mathrm{QM}}$, $\mathbf{G }_{\mathrm{Q}}$ and $\mathbf{G }_{\mathrm{M}}$ to understand the properties of the REML estimators for the scenarios we simulated. Using the eigen-decompositions, we have:

52 $$\begin{aligned} \mathbf{G }_{\mathrm{QM}} = \frac{\hbox {q}}{\hbox {q}+\hbox {m}}\mathbf{G }_{\mathrm{Q}} + \frac{\hbox {m}}{\hbox {q}+\hbox {m}}\mathbf{G }_{\mathrm{M}} = \left( 1 - \frac{\hbox {m}}{\hbox {q}+\hbox {m}}\right) \mathbf{U }_{\mathrm{Q}}\varvec{\Lambda }_{\mathrm{Q}}\mathbf{U }_{\mathrm{Q}}' + \frac{\hbox {m}}{\hbox {q}+\hbox {m}}\mathbf{U }_{\mathrm{M}}\varvec{\Lambda }_{\mathrm{M}}\mathbf{U }_{\mathrm{M}}'. \end{aligned}$$

Now, because $\mathbf{G }_{\mathrm{QM}} = \mathbf{U }_{\mathrm{QM}}\varvec{\Lambda }_{\mathrm{QM}}\mathbf{U }_{\mathrm{QM}}'$, using that identity in Eq. (52) we have:

53 $$\begin{aligned} \mathbf{U }_{\mathrm{QM}}\varvec{\Lambda }_{\mathrm{QM}}\mathbf{U }_{\mathrm{QM}}' = \left( 1 - \frac{\hbox {m}}{\hbox {q}+\hbox {m}}\right) \mathbf{U }_{\mathrm{Q}}\varvec{\Lambda }_{\mathrm{Q}}\mathbf{U }_{\mathrm{Q}}' + \frac{\hbox {m}}{\hbox {q}+\hbox {m}}\mathbf{U }_{\mathrm{M}}\varvec{\Lambda }_{\mathrm{M}}\mathbf{U }_{\mathrm{M}}', \end{aligned}$$

hence,

54 $$\begin{aligned} \varvec{\Lambda }_{\mathrm{QM}} = \left( 1 - \frac{\hbox {m}}{\hbox {q}+\hbox {m}}\right) \mathbf{U }_{\mathrm{QM}}'\mathbf{U }_{\mathrm{Q}}\varvec{\Lambda }_{\mathrm{Q}}\mathbf{U }_{\mathrm{Q}}'\mathbf{U }_{\mathrm{QM}} + \frac{\hbox {m}}{\hbox {q}+\hbox {m}}\mathbf{U }_{\mathrm{QM}}'\mathbf{U }_{\mathrm{M}}\varvec{\Lambda }_{\mathrm{M}}\mathbf{U }_{\mathrm{M}}'\mathbf{U }_{\mathrm{QM}}. \end{aligned}$$

Therefore the elements in the diagonal of $\varvec{\Lambda }_{\mathrm{QM}}$ obey the following identity,

55 $$\begin{aligned} \lambda _{\mathrm{QMi}} = \left( 1 - \frac{\hbox {m}}{\hbox {q}+\hbox {m}}\right) \sum _{\mathrm{k}=1}^{\mathrm{n}}\left( \mathbf{U }_{\mathrm{QMi}}'\mathbf{U }_{\mathrm{Qk}}\right) ^{2}\lambda _{\mathrm{Qk}} + \frac{\hbox {m}}{\hbox {q}+\hbox {m}}\sum _{\mathrm{k}=1}^{\mathrm{n}}\left( \mathbf{U }_{\mathrm{QMi}}'\mathbf{U }_{\mathrm{Mk}}\right) ^{2}\lambda _{\mathrm{Mk}}. \end{aligned}$$

Isolating $\sum _{\mathrm{k}=1}^{\mathrm{n}}\left( \mathbf{U }_{\mathrm{QMi}}'\mathbf{U }_{\mathrm{Qk}}\right) ^{2}\lambda _{\mathrm{Qk}}$ in Eq. (55) gives us the following:

56 $$\begin{aligned} \sum _{\mathrm{k}=1}^{\mathrm{n}}\left( \mathbf{U }_{\mathrm{QMi}}'\mathbf{U }_{\mathrm{Qk}}\right) ^{2}\lambda _{\mathrm{Qk}} = \lambda _{\mathrm{QMi}} + \frac{\hbox {m}}{\hbox {q}+\hbox {m}}\left[ \sum _{\mathrm{k}=1}^{\mathrm{n}}\left( \mathbf{U }_{\mathrm{QMi}}'\mathbf{U }_{\mathrm{Qk}}\right) ^{2}\lambda _{\mathrm{Qk}} - \sum _{\mathrm{k}=1}^{\mathrm{n}}\left( \mathbf{U }_{\mathrm{QMi}}'\mathbf{U }_{\mathrm{Mk}}\right) ^{2}\lambda _{\mathrm{Mk}}\right] . \end{aligned}$$

Applying Eq. (38) to $\sum _{\mathrm{k}=1}^{\mathrm{n}}\left( \mathbf{U }_{\mathrm{QMi}}'\mathbf{U }_{\mathrm{Qk}}\right) ^{2}\lambda _{\mathrm{Qk}}$ and $\sum _{\mathrm{k}=1}^{\mathrm{n}}\left( \mathbf{U }_{\mathrm{QMi}}'\mathbf{U }_{\mathrm{Mk}}\right) ^{2}\lambda _{\mathrm{Mk}}$, we have:

57 $$\begin{aligned} \sum _{\mathrm{k}=1}^{\mathrm{n}}\left( \mathbf{U }_{\mathrm{QMi}}'\mathbf{U }_{\mathrm{Qk}}\right) ^{2}\lambda _{\mathrm{Qk}} = 1 + \sum _{\mathrm{j}=1}^{\mathrm{n}}\mathrm{U}_{\mathrm{QMij}}^{2}\delta _{\mathrm{Qjj}} + \sum _{\mathrm{j}=1}^{\mathrm{n}}\sum _{\mathrm{l} \ne \mathrm{j}}\mathrm{U}_{\mathrm{QMij}}\mathrm{U}_{\mathrm{QMil}}\left( \mathrm{a}_{\mathrm{jl}} + \delta _{\mathrm{Qjl}}\right) , \end{aligned}$$

and

58 $$\begin{aligned} \sum _{\mathrm{k}=1}^{\mathrm{n}}\left( \mathbf{U }_{\mathrm{QMi}}'\mathbf{U }_{\mathrm{Mk}}\right) ^{2}\lambda _{\mathrm{Mk}} = 1 + \sum _{\mathrm{j}=1}^{\mathrm{n}}\hbox {U}_{\mathrm{QMij}}^{2}\delta _{\mathrm{Mjj}} + \sum _{\mathrm{j}=1}^{\mathrm{n}}\sum _{\mathrm{l} \ne \mathrm{j}}\hbox {U}_{\mathrm{QMij}}\hbox {U}_{\mathrm{QMil}}\left( \hbox {a}_{\mathrm{jl}} + \delta _{\mathrm{Mjl}}\right) . \end{aligned}$$

Finally, using the identities from Eqs. (57) and (58) in Eq. (56):

59 $$\begin{aligned} \sum _{\mathrm{k}=1}^{\mathrm{n}}\left( \mathbf{U }_{\mathrm{QMi}}'\mathbf{U }_{\mathrm{Qk}}\right) ^{2}\lambda _{\mathrm{Qk}} = \lambda _{\mathrm{QMi}} + \delta _{\mathrm{i}}, \end{aligned}$$

with $$\delta _{\mathrm{i}} = \frac{\hbox {m}}{\hbox {q}+\hbox {m}}\sum _{\mathrm{j}=1}^{\mathrm{n}}\sum _{\mathrm{l}=1}^{\mathrm{n}}\mathrm{U}_{\mathrm{QMij}}\mathrm{U}_{\mathrm{QMil}}\left( \delta _{\mathrm{Qjl}} - \delta _{\mathrm{Mjl}}\right)$$.

To understand how $\delta _{\mathrm{i}}$ will affect the relationship between $\sum _{\mathrm{k}=1}^{\mathrm{n}}\,\,\left( \mathbf{U }_{\mathrm{QMi}}'\mathbf{U }_{\mathrm{Qk}}\right) ^{2}\lambda _{\mathrm{Qk}}$ and $\lambda _{\mathrm{QMi}}$, it is fundamental to understand $\mathbb {E}\left( \delta _{\mathrm{i}}\right)$.

60 $$\begin{aligned} \mathbb {E}\left( \delta _{\mathrm{i}}\right) &= \frac{\hbox {m}}{\hbox {q}+\hbox {m}}\sum _{\mathrm{j}=1}^{\mathrm{n}}\sum _{\mathrm{l}=1}^{\mathrm{n}}\mathbb {E}\left( \hbox {U}_{\mathrm{QMij}}\hbox {U}_{\mathrm{QMil}}\left[ \delta _{\mathrm{Qjl}} - \delta _{\mathrm{Mjl}}\right] \right) \nonumber \\&= \frac{\hbox {m}}{\hbox {q}+\hbox {m}}\sum _{\mathrm{j}=1}^{\mathrm{n}}\sum _{\mathrm{l}=1}^{\mathrm{n}}\left[ \mathbb {E}\left( \hbox {U}_{\mathrm{QMij}}\hbox {U}_{\mathrm{QMil}}\delta _{\mathrm{Qjj}}\right) - \mathbb {E}\left( \hbox {U}_{\mathrm{QMij}}\hbox {U}_{\mathrm{QMil}}\delta _{\mathrm{Mjj}}\right) \right] \nonumber \\&= \frac{\hbox {m}}{\hbox {q}+\hbox {m}}\sum _{\mathrm{j}=1}^{\mathrm{n}}\sum _{\mathrm{l}=1}^{\mathrm{n}}\left[ \mathbb {C}\hbox {ov}\left( \hbox {U}_{\mathrm{QMij}}\hbox {U}_{\mathrm{QMil}},\delta _{\mathrm{Qjj}}\right) - \mathbb {C}\hbox {ov}\left( \hbox {U}_{\mathrm{QMij}}\hbox {U}_{\mathrm{QMil}},\delta _{\mathrm{Mjj}}\right) \right] \nonumber \\&= \frac{\hbox {m}}{\hbox {q}+\hbox {m}}\sum _{\mathrm{j}=1}^{\mathrm{n}}\sum _{\mathrm{l}=1}^{\mathrm{n}}\left[ \mathbb {C}\hbox {ov}\left( \hbox {U}_{\mathrm{QMij}}\hbox {U}_{\mathrm{QMil}},\hbox {G}_{\mathrm{Qjj}} - \hbox {a}_{\mathrm{jj}}\right) - \mathbb {C}\hbox {ov}\left( \hbox {U}_{\mathrm{QMij}}\hbox {U}_{\mathrm{QMil}},\hbox {G}_{\mathrm{Mjj}} - \hbox {a}_{\mathrm{jj}}\right) \right] \nonumber \\&= \frac{\hbox {m}}{\hbox {q}+\hbox {m}}\sum _{\mathrm{j}=1}^{\mathrm{n}}\sum _{\mathrm{l}=1}^{\mathrm{n}}\left[ \mathbb {C}\hbox {ov}\left( \hbox {U}_{\mathrm{QMij}}\hbox {U}_{\mathrm{QMil}},\hbox {G}_{\mathrm{Qjj}}\right) - \mathbb {C}\hbox {ov}\left( \hbox {U}_{\mathrm{QMij}}\hbox {U}_{\mathrm{QMil}},\hbox {G}_{\mathrm{Mjj}}\right) \right] \nonumber \\&= \frac{\hbox {m}}{\hbox {q}+\hbox {m}}\sum _{\mathrm{j}=1}^{\mathrm{n}}\sum _{\mathrm{l}=1}^{\mathrm{n}}\mathbb {C}\hbox {ov}\left( \hbox {U}_{\mathrm{QMij}}\hbox {U}_{\mathrm{QMil}},\hbox {G}_{\mathrm{Qjj}}\right) \nonumber \\&\quad- \frac{\hbox {m}}{\hbox {q}+\hbox {m}}\sum _{\mathrm{j}=1}^{\mathrm{n}}\sum _{\mathrm{l}=1}^{\mathrm{n}}\mathbb {C}\hbox {ov}\left( \mathrm{U}_{\mathrm{QMij}}\hbox {U}_{\mathrm{QMil}},\left[ 1 + \frac{\hbox {q}}{\hbox {m}}\right] \hbox {G}_{\mathrm{QMjj}} - \frac{\hbox {q}}{\hbox {m}}\hbox {G}_{\mathrm{Qjj}}\right) \nonumber \\&= \frac{\hbox {m}}{\hbox {q}+\hbox {m}}\left( 1 + \frac{\hbox {q}}{\hbox {m}}\right) \sum _{\mathrm{j}=1}^{\mathrm{n}}\sum _{\mathrm{l}=1}^{\mathrm{n}}\mathbb {C}\hbox {ov}\left( \hbox {U}_{\mathrm{QMij}}\hbox {U}_{\mathrm{QMil}},\hbox {G}_{\mathrm{Qjj}}\right) \nonumber \\&\quad- \frac{\hbox {m}}{\hbox {q}+\hbox {m}}\left( 1 + \frac{\hbox {q}}{\hbox {m}}\right) \sum _{\mathrm{j}=1}^{\mathrm{n}}\sum _{\mathrm{l}=1}^{\mathrm{n}}\mathbb {C}\hbox {ov}\left( \hbox {U}_{\mathrm{QMij}}\hbox {U}_{\mathrm{QMil}},\hbox {G}_{\mathrm{QMjj}}\right) \nonumber \\&= \sum _{\mathrm{j}=1}^{\mathrm{n}}\sum _{\mathrm{l}=1}^{\mathrm{n}}\left[ \mathbb {C}\hbox {ov}\left( \hbox {U}_{\mathrm{QMij}}\hbox {U}_{\mathrm{QMil}},\hbox {G}_{\mathrm{Qjl}}\right) - \mathbb {C}\hbox {ov}\left( \hbox {U}_{\mathrm{QMij}}\hbox {U}_{\mathrm{QMil}},\hbox {G}_{\mathrm{QMjl}}\right) \right] \nonumber \\&= \sum _{\mathrm{j}=1}^{\mathrm{n}}\sum _{\mathrm{l}=1}^{\mathrm{n}}\left( \sigma _{\mathrm{ijl},\mathrm{Qlj}} - \sigma _{\mathrm{ijl},\mathrm{jl}}\right) \end{aligned}$$

Thus, $\mathbb {E}\left( \delta _{\mathrm{i}}\right) = \sum _{\mathrm{j}=1}^{\mathrm{n}}\sum _{\mathrm{l}=1}^{\mathrm{n}}\left( \sigma _{\mathrm{ijl},\mathrm{Qlj}} - \sigma _{\mathrm{ijl},\mathrm{jl}}\right)$. It is intuitive that $\mathbb {C}\hbox {ov}\left( \hbox {U}_{\mathrm{QMij}}\hbox {U}_{\mathrm{QMil}},\hbox {G}_{\mathrm{QMjl}}\right) \ge \mathbb {C}\hbox {ov}\left( \hbox {U}_{\mathrm{QMij}}\hbox {U}_{\mathrm{QMil}},\hbox {G}_{\mathrm{Qjl}}\right)$, therefore $\mathbb {E}\left( \delta _{\mathrm{i}}\right) \le 0$.

### F.2 Markers only

When we use the genomic model containing the markers only, from Eq. (38) it is straightforward that:

61 $$\begin{aligned} \sum _{\mathrm{k}=1}^{\mathrm{n}}\left( \mathbf{U }_{\mathrm{Mi}}'\mathbf{U }_{\mathrm{Qk}}\right) ^{2}\lambda _{\mathrm{Qk}} = 1 + \sum _{\mathrm{j}=1}^{\mathrm{n}}\hbox {U}_{\mathrm{Mij}}^{2}\delta _{\mathrm{Qjj}} + \sum _{\mathrm{j}=1}^{\mathrm{n}}\sum _{\mathrm{l} \ne \mathrm{j}}\hbox {U}_{\mathrm{Mij}}\hbox {U}_{\mathrm{Mil}}\left( \hbox {a}_{\mathrm{jl}} + \delta _{\mathrm{Qjl}}\right) . \end{aligned}$$

If we randomly pick just a few markers, they will most likely be in low LD with the QTL ($\mathbf{U }_{\mathrm{Mi}}'\mathbf{U }_{\mathrm{Qk}} \approx 0$ for any $\hbox {i},\hbox {k}=1,\ldots ,\hbox {n}$) and $\sum _{\mathrm{k}=1}^{\mathrm{n}}\left( \mathbf{U }_{\mathrm{Mi}}'\mathbf{U }_{\mathrm{Qk}}\right) ^{2}\lambda _{\mathrm{Qk}} \approx 0$. When the density of marker data increases, $\sum _{\mathrm{k}=1}^{\mathrm{n}}\left( \mathbf{U }_{\mathrm{Mi}}'\mathbf{U }_{\mathrm{Qk}}\right) ^{2}\lambda _{\mathrm{Qk}}$ also increases, but Eq. (61) is not so straightforward to understand analytically. Therefore, we use the identity $\mathbf{G }_{\mathrm{QM}} = \hbox {q}(\hbox {q}+\hbox {m})^{-1}\mathbf{G }_{\mathrm{Q}} + \hbox {m}(\hbox {q}+\hbox {m})^{-1}\mathbf{G }_{\mathrm{M}}$ to help us understand what happens when we use a genomic model that contains only markers in LD with the QTL in the SNP data. When the number of markers is sufficiently large, $\lim _{\mathrm{m}\rightarrow \infty }\mathbf{G }_{\mathrm{M}} = \lim _{\mathrm{m}\rightarrow \infty }\mathbf{G }_{\mathrm{QM}}$, and consequently $\lim _{\mathrm{m}\rightarrow \infty }\sum _{\mathrm{k}=1}^{\mathrm{n}}\left( \mathbf{U }_{\mathrm{Mi}}'\mathbf{U }_{\mathrm{Qk}}\right) ^{2}\lambda _{\mathrm{Qk}} = \lim _{\mathrm{m}\rightarrow \infty }\sum _{\mathrm{k}=1}^{\mathrm{n}}\left( \mathbf{U }_{\mathrm{QMi}}'\mathbf{U }_{\mathrm{Qk}}\right) ^{2}\lambda _{\mathrm{Qk}}$. This means that when we have a genomic model with markers only, $\mathbf{G }_{\mathrm{M}}$ will be able to explain, at most, the same amount of variability that $\mathbf{G }_{\mathrm{QM}}$ can explain.

## Appendix 7: Algorithm used to simulate genotype data

Given a sample size $\hbox {n}$, a number of markers $\hbox {m}$ and a number of QTL $\hbox {q}$:

1. Generate $\varvec{\theta }_{(\mathrm{m}) \times 1} = \left[ \varvec{\theta }_{\mathrm{Q}} \quad \varvec{\theta }_{\mathrm{M}}\right]$ a vector of MAF for all the $\hbox {m}$ markers, such that:
  - if QTL/marker are common variants: $\theta _{\mathrm{Qj}} {\mathop {\sim }\limits ^{\mathrm{iid}}} \hbox {U}(0.05,0.5) \forall \hbox {j}=1,\ldots ,\mathrm{q}$, $\theta _{\mathrm{Mj}} {\mathop {\sim }\limits ^{\mathrm{iid}}} \hbox {U}(0.05,0.5) \forall \hbox {j}=1,\ldots ,\mathrm{m}$;
  - if QTL/marker are rare variants: $\theta _{\mathrm{Qj}} {\mathop {\sim }\limits ^{\mathrm{iid}}} \hbox {U}(0.01,0.03) \forall \hbox {j}=1,\ldots ,\mathrm{q}$, $\theta _{\mathrm{Mj}} {\mathop {\sim }\limits ^{\mathrm{iid}}} \hbox {U}(0.01,0.03) \forall \hbox {j}=1,\ldots ,\mathrm{m}$;
2. Generate $\hbox {n} \times (\hbox {q}+\hbox {m})$ genotype matrix $\mathbf{Z }$ containing both QTL and markers:
  - if the SNPs are independent: $\hbox {Z}_{\mathrm{1j}},\ldots ,\hbox {Z}_{\mathrm{nj}} {\mathop {\sim }\limits ^{\mathrm{iid}}} \hbox {B}(2,\theta _{\mathrm{j}})$, $\forall \hbox {j}=1,\ldots ,\hbox {q}+\hbox {m}$;
  - if the SNPs are correlated, use a routine to generate the correlated binomial data (details in next algorithm), such that:
    $$\bullet$$ $$(\hbox {Z}_{\mathrm{1j}},\ldots ,\hbox {Z}_{\mathrm{nj}})\mid \varvec{\theta } {\mathop {\sim }\limits ^{\mathrm{iid}}} \hbox {B}(2,\theta _{\mathrm{j}})$$;
    $$\bullet$$ $$\mathbb {C}\hbox {or}(\hbox {Z}_{\mathrm{ij}},\hbox {Z}_{\mathrm{il}}) = \rho _{\mathrm{jl}} \forall \hbox {i}=1,\ldots ,\hbox {n}$$;
3. Obtain the standardized genotype matrix $\mathbf{W }$,
  - $$\hat{\theta }_{\mathrm{j}} = (1/2\hbox {n})\sum _{\mathrm{i}=1}^{\mathrm{n}}\hbox {Z}_{\mathrm{ij}} \forall \hbox {j}=1,\ldots ,\hbox {q}+\hbox {m}$$,
  - $$\hbox {w}_{\mathrm{ij}} = \frac{\hbox {z}_{\mathrm{ij}} - 2\hat{\theta }_{\mathrm{j}}}{\sqrt{2\hat{\theta }_{\mathrm{j}}(1 - \hat{\theta }_{\mathrm{j}})}} \forall \hbox {i}=1,\ldots ,\hbox {n}$$, $$\hbox {j}=1,\ldots ,\mathrm{q}+\mathrm{m}$$,
4. Use the group of $\hbox {q}$ simulated QTL to create $\mathbf{W }_{\mathrm{Q}}$,
5. Use the group of *m* simulated markers to create $\mathbf{W }_{\mathrm{M}}$.

## Appendix 8: Algorithm used to simulate correlated SNP data

In order to simulate correlated binary data, we have looked into approaches such as the Bahadur’s representation [25], and the algorithms of [26–28] and [29]. Finally, we have opted for the use of a method that involves the multivariate normal distribution, as proposed by [30].

1. Given the vector $\varvec{\theta }_{(\mathrm{q}+\mathrm{m}) \times 1}$ of MAF, calculate $\mu _{\mathrm{j}} = \Phi ^{-1}(\theta _{\mathrm{j}}) \forall \hbox {j}=1,\ldots ,\mathrm{q}+\mathrm{m}$,
2. For a matrix $\varvec{\Xi } = [\xi _{\mathrm{kj}}]_{\mathrm{k},\mathrm{j}=1}^{\mathrm{q}+\mathrm{m}}$, simulate $\mathbf{X }_{\mathrm{n}\times (\mathrm{p+m})}^{(1)},\mathbf{X }_{\mathrm{n}\times (\mathrm{q}+\mathrm{m})}^{(2)} {\mathop {\sim }\limits ^{\mathrm{iid}}} \hbox {N}(\varvec{\mu },\varvec{\Xi })$,
3. $$\forall \hbox {i}=1,\ldots ,\hbox {n}$$, $$\hbox {j}=1,\ldots ,\hbox {q}+\hbox {m}$$:
  - $$\hbox {Z}_{\mathrm{ij}}^{(1)} = \mathbb {I}\{\hbox {X}_{\mathrm{ij}}^{(1)} > 0\}$$: $$\mathbb {P}(\hbox {X}_{\mathrm{ij}}^{(1)} > 0) = \theta _{\mathrm{j}}$$;
  - $$\hbox {Z}_{\mathrm{ij}}^{(2)} = \mathbb {I}\{\hbox {X}_{\mathrm{ij}}^{(2)} > 0\}$$: $$\mathbb {P}(\hbox {X}_{\mathrm{ij}}^{(2)} > 0) = \theta _{\mathrm{j}}$$;
4. $$\hbox {Z}_{\mathrm{ij}} = \hbox {Z}_{\mathrm{ij}}^{(1)} + \hbox {Z}_{\mathrm{ij}}^{(2)}$$, such that:
  - $$\rho _{\mathrm{kj}} = \mathbb {C}\hbox {or}(\hbox {Z}_{\mathrm{ik}},\hbox {Z}_{\mathrm{ij}}) = \mathbb {C}\hbox {or}(\hbox {Z}_{\mathrm{ik}}^{(1)},\hbox {Z}_{\mathrm{ij}}^{(1)}) = \frac{\mathbb {E}\left( \hbox {Z}_{\mathrm{ik}}^{(1)}\hbox {Z}_{\mathrm{ij}}^{(1)}\right) - \theta _{\mathrm{k}}\theta _{\mathrm{j}}}{\sqrt{\theta _{\mathrm{k}}(1-\theta _{\mathrm{k}})\theta _{\mathrm{j}}(1-\theta _{\mathrm{j}})}}$$, $$\forall \hbox {k} \ne \mathrm{j}$$;
  - $$\mathbb {E}\left( \hbox {Z}_{\mathrm{ik}}^{(1)}\hbox {Z}_{\mathrm{ij}}^{(1)}\right) = \mathbb {P}\left( \hbox {X}_{\mathrm{ik}}^{(1)}> 0,\hbox {X}_{\mathrm{ij}}^{(1)} > 0\right) = \mathbb {P}\left( \hbox {X}_{\mathrm{ik}}^{(1)}< 0,\hbox {X}_{\mathrm{ij}}^{(1)} < 0\right) + \theta _{\mathrm{j}} + \theta _{\mathrm{k}} - 1$$.

Details about how to relate input matrix $\varvec{\Xi }$ to the output correlation matrix $\varvec{\Sigma }$ between SNPs are in “Appendix 10”.

## Appendix 9: Algorithm used to simulate phenotype data

Setting $$\sigma ^{2}_{\mathrm{b}_{\mathrm{Q}}}=(1/\mathrm{q})\mathrm{h}^{2}$$ and $$\sigma ^{2}_{\varepsilon _{\mathrm{Q}}}=1-\mathrm{h}^{2}$$:

1. Generate $$\mathbf{b }_{\mathrm{Q}} {\mathop {\sim }\limits ^{\mathrm{iid}}} \hbox {N}(\mathbf{0 },\mathbf{I }_{\mathrm{q}}\sigma ^{2}_{\mathrm{b}_{\mathrm{Q}}})$$,
2. Generate $$\varvec{\varepsilon }_{\mathrm{Q}} {\mathop {\sim }\limits ^{\mathrm{iid}}} \hbox {N}(\mathbf{0 },\mathbf{I }_{\mathrm{n}}\sigma ^{2}_{\varepsilon _{\mathrm{Q}}})$$,
3. Define a value to $\mu$,
4. Calculate $$\mathbf{y } = \mathbf{1 }\mu + \mathbf{W }_{\mathrm{Q}} \mathbf{b }_{\mathrm{Q}} + \varvec{\varepsilon }_{\mathrm{Q}}$$.

## Appendix 10: Correlation structure between the SNPs

Our aim was to simulate genotypes according to a particular correlation structure between the SNPs, defined by $\varvec{\Sigma }$. It is very important to note that $\varvec{\Sigma } \ne \varvec{\Xi }$. Thus, we need to describe here how we defined the elements in matrix $\varvec{\Xi }$, so that after the data was simulated, we obtained the desired $\varvec{\Sigma }$. Without loss of generality, we can define straight away that the diagonal elements of $\varvec{\Xi }$ are all 1s.

Keeping in mind that $\theta _{\mathrm{j}} {\mathop {\sim }\limits ^{\hbox {indep}.}} \hbox {U}(\hbox {a}_{\mathrm{j}},\hbox {b}_{\mathrm{j}})$, $\mu _{\mathrm{j}} = \Phi ^{-1}(\hbox {t}_{\mathrm{j}})$ and the joint cumulative normal distribution $\mathbb {P}\left( \hbox {X}_{\mathrm{ik}}^{(1)}< 0,\hbox {X}_{\mathrm{ij}}^{(1)} < 0\right) = \Phi _{2}(-\mu _{\mathrm{j}},-\mu _{\mathrm{k}},\xi _{\mathrm{kj}}) = \int _{-\infty }^{-\mu _{\mathrm{j}}}\int _{-\infty }^{-\mu _{\mathrm{k}}} \frac{1}{2\pi \sqrt{1-\xi ^{2}_{\mathrm{jk}}}} \exp \left[ \frac{-(\hbox {u}^{2} - 2\xi _{\mathrm{kj}}\hbox {uv} +\hbox {v}^{2})}{2(1-\xi ^{2}_{\mathrm{jk}})}\right] \hbox {dudv}$, which has to be evaluated numerically [31], and defining $\varvec{\Sigma }=[\mathbb {E}_{\varvec{\theta }}(\rho _{\mathrm{jk}})]_{\mathrm{k},\mathrm{j}=1}^{\mathrm{p}+\mathrm{m}}$, such that $\mathbb {E}_{\varvec{\theta }}(\rho _{\mathrm{jk}}) = \rho _{\mid \mathrm{k}-\mathrm{j} \mid }$, for all $\mathrm{j} \ne \mathrm{k}$:

62 $$\begin{aligned} \rho _{\mid \mathrm{j}-\mathrm{k} \mid }= & \mathbb {E}_{\varvec{\theta }}\left( \mathbb {C}\hbox {or}(\hbox {Z}_{\mathrm{ik}},\hbox {Z}_{\mathrm{ij}})\right) = \int _{\mathrm{a}_{\mathrm{j}}}^{\hbox {b}_{\mathrm{j}}}\int _{\mathrm{a}_{\mathrm{k}}}^{\hbox {b}_{\mathrm{k}}} \mathbb {C}\hbox {or}(\hbox {Z}_{\mathrm{ik}},\hbox {Z}_{\mathrm{ij}}) \hbox {f}_{\theta _{\mathrm{j}}}(\hbox {t}_{\mathrm{j}})\hbox {f}_{\theta _{\mathrm{k}}}(\hbox {t}_{\mathrm{k}}) \mathrm{dt}_{\mathrm{k}}\mathrm{dt}_{\mathrm{j}} \nonumber \\= & \int _{\mathrm{a}_{\mathrm{j}}}^{\mathrm{b}_{\mathrm{j}}}\int _{\mathrm{a}_{\mathrm{k}}}^{\mathrm{b}_{\mathrm{k}}} \frac{\left[ \mathbb {P}(\mathrm{X}_{\mathrm{ik}}^{(1)}> 0,\hbox {X}_{\mathrm{ij}}^{(1)} > 0) - \hbox {t}_{\mathrm{k}}\mathrm{t}_{\mathrm{j}}\right] }{\sqrt{\mathrm{t}_{\mathrm{k}}(1-\mathrm{t}_{\mathrm{k}})\mathrm{t}_{\mathrm{j}}(1-\mathrm{t}_{\mathrm{j}})}} \mathrm{f}_{\theta _{\mathrm{j}}}(\mathrm{t}_{\mathrm{j}})\mathrm{f}_{\theta _{\mathrm{k}}}(\mathrm{t}_{\mathrm{k}}) \mathrm{dt}_{\mathrm{k}}\mathrm{dt}_{\mathrm{j}} \nonumber \\= & \int _{\mathrm{a}_{\mathrm{j}}}^{\mathrm{b}_{\mathrm{j}}}\int _{\mathrm{a}_{\mathrm{k}}}^{\mathrm{b}_{\mathrm{k}}} \frac{\left[ \Phi _{2}(-\mu _{\mathrm{j}},-\mu _{\mathrm{k}},\xi _{\mathrm{kj}}) + \hbox {t}_{\mathrm{j}} + \hbox {t}_{\mathrm{k}} - \hbox {t}_{\mathrm{j}}\hbox {t}_{\mathrm{k}} - 1\right] }{\sqrt{\hbox {t}_{\mathrm{k}}(1-\mathrm{t}_{\mathrm{k}})\hbox {t}_{\mathrm{j}}(1-\mathrm{t}_{\mathrm{j}})}} \hbox {f}_{\theta _{\mathrm{j}}}(\hbox {t}_{\mathrm{j}})\hbox {f}_{\theta _{\mathrm{k}}}(\hbox {t}_{\mathrm{k}}) \hbox {dt}_{\mathrm{k}}\mathrm{dt}_{\mathrm{j}} \nonumber \\= & \int _{\mathrm{a}_{\mathrm{j}}}^{\mathrm{b}_{\mathrm{j}}}\int _{\mathrm{a}_{\mathrm{k}}}^{\mathrm{b}_{\mathrm{k}}} \left[ \frac{\Phi _{2}(-\mu _{\mathrm{j}},-\mu _{\mathrm{k}},\xi _{\mathrm{kj}})}{\sqrt{\mathrm{t}_{\mathrm{k}}(1-\mathrm{t}_{\mathrm{k}})\mathrm{t}_{\mathrm{j}}(1-\mathrm{t}_{\mathrm{j}})}} - \sqrt{\frac{(1-\mathrm{t}_{\mathrm{j}})(1-\mathrm{t}_{\mathrm{k}})}{\mathrm{t}_{\mathrm{j}}\mathrm{t}_{\mathrm{k}}}}\right] \mathrm{f}_{\theta _{\mathrm{j}}}(\mathrm{t}_{\mathrm{j}})\mathrm{f}_{\theta _{\mathrm{k}}}(\mathrm{t}_{\mathrm{k}}) \mathrm{dt}_{\mathrm{k}}\mathrm{dt}_{\mathrm{j}} \nonumber \\= & \frac{1}{(\mathrm{b}_{\mathrm{j}}-\mathrm{a}_{\mathrm{j}})(\mathrm{b}_{\mathrm{k}}-\mathrm{a}_{\mathrm{k}})} \int _{\mathrm{a}_{\mathrm{j}}}^{\mathrm{b}_{\mathrm{j}}}\int _{\mathrm{a}_{\mathrm{k}}}^{\mathrm{b}_{\mathrm{k}}} \frac{\Phi _{2}(-\mu _{\mathrm{j}},-\mu _{\mathrm{k}},\xi _{\mathrm{kj}})}{\sqrt{\mathrm{t}_{\mathrm{k}}(1-\mathrm{t}_{\mathrm{k}})\mathrm{t}_{\mathrm{j}}(1-\mathrm{t}_{\mathrm{j}})}} \mathrm{dt}_{\mathrm{k}}\mathrm{dt}_{\mathrm{j}} \nonumber \\&- \left. \left[ \frac{\sqrt{\mathrm{u}(1-\mathrm{u})}}{(\mathrm{b}_{\mathrm{j}}-\mathrm{a}_{\mathrm{j}})} - \frac{\mathrm{arctg}\left( \sqrt{\frac{1-\mathrm{u}}{\mathrm{u}}}\right) }{(\mathrm{b}_{\mathrm{j}}-\mathrm{a}_{\mathrm{j}})}\right] \right| _{\mathrm{a}_{\mathrm{j}}}^{\mathrm{b}_{\mathrm{j}}} \times \left. \left[ \frac{\sqrt{\mathrm{u}(1-\mathrm{u})}}{(\mathrm{b}_{\mathrm{k}}-\mathrm{a}_{\mathrm{k}})} - \frac{\mathrm{arctg}\left( \sqrt{\frac{1-\mathrm{u}}{\mathrm{u}}}\right) }{(\mathrm{b}_{\mathrm{k}}-\mathrm{a}_{\mathrm{k}})}\right] \right| _{\mathrm{a}_{\mathrm{k}}}^{\mathrm{b}_{\mathrm{k}}}. \end{aligned}$$

Thus, when we have the value for $\rho _{\mid \mathrm{k}-\mathrm{j} \mid }$ and the MAF’s $\theta _{\mathrm{j}}$ and $\theta _{\mathrm{k}}$, along with their uniform distributions boundaries $(\mathrm{a}_{\mathrm{j}},\mathrm{b}_{\mathrm{j}})$ and $(\mathrm{a}_{\mathrm{k}},\mathrm{b}_{\mathrm{k}})$, from Eq. (62) we define the function of $\xi _{\mathrm{kj}}$,

63 $$\begin{aligned} \mathrm{f}(\xi _{\mathrm{kj}})= & - \rho _{\mid \mathrm{j}-\mathrm{k} \mid } + \frac{1}{(\mathrm{b}_{\mathrm{j}}-\mathrm{a}_{\mathrm{j}})(\mathrm{b}_{\mathrm{k}}-\mathrm{a}_{\mathrm{k}})} \int _{\mathrm{a}_{\mathrm{j}}}^{\mathrm{b}_{\mathrm{j}}}\int _{\mathrm{a}_{\mathrm{k}}}^{\mathrm{b}_{\mathrm{k}}} \frac{\Phi _{2}(-\mu _{\mathrm{j}},-\mu _{\mathrm{k}},\xi _{\mathrm{kj}})}{\sqrt{\mathrm{t}_{\mathrm{k}}(1-\mathrm{t}_{\mathrm{k}})\mathrm{t}_{\mathrm{j}}(1-\mathrm{t}_{\mathrm{j}})}} \mathrm{dt}_{\mathrm{k}}\mathrm{dt}_{\mathrm{j}} \nonumber \\&- \left. \left[ \frac{\sqrt{\mathrm{u}(1-\mathrm{u})}}{(\mathrm{b}_{\mathrm{j}}-\mathrm{a}_{\mathrm{j}})} - \frac{\mathrm{arctg}\left( \sqrt{\frac{1-\mathrm{u}}{\mathrm{u}}}\right) }{(\mathrm{b}_{\mathrm{j}}-\mathrm{a}_{\mathrm{j}})}\right] \right| _{\mathrm{a}_{\mathrm{j}}}^{\mathrm{b}_{\mathrm{j}}} \times \left. \left[ \frac{\sqrt{\mathrm{u}(1-\mathrm{u})}}{(\mathrm{b}_{\mathrm{k}}-\mathrm{a}_{\mathrm{k}})} - \frac{\mathrm{arctg}\left( \sqrt{\frac{1-\mathrm{u}}{\mathrm{u}}}\right) }{(\mathrm{b}_{\mathrm{k}}-\mathrm{a}_{\mathrm{k}})}\right] \right| _{\mathrm{a}_{\mathrm{k}}}^{\mathrm{b}_{\mathrm{k}}}. \end{aligned}$$

Finally, each element $\xi _{\mathrm{kj}}$ from the correlation matrix $\varvec{\Xi }$, that must be used to simulate $\mathbf{X }_{\mathrm{n}\times (\mathrm{q}+\mathrm{m})}^{(1)}$, $\mathbf{X }_{\mathrm{n}\times (\mathrm{q}+\mathrm{m})}^{(2)}$ ${\mathop {\sim }\limits ^{\mathrm{iid}}} \mathrm{N}(\varvec{\mu },\varvec{\Xi })$, is the root of $\hbox {f}(\xi _{\mathrm{kj}})$, which can be obtained by using numerical methods.
