# Supplementary figures and images for: Understanding the potential bias of variance components estimators when using genomic models
Source: Genet Sel Evol. 2018 Aug 6;50:41. doi: 10.1186/s12711-018-0411-0 (PMC6080367; doi:10.1186/s12711-018-0411-0)

Supplementary Figure 1

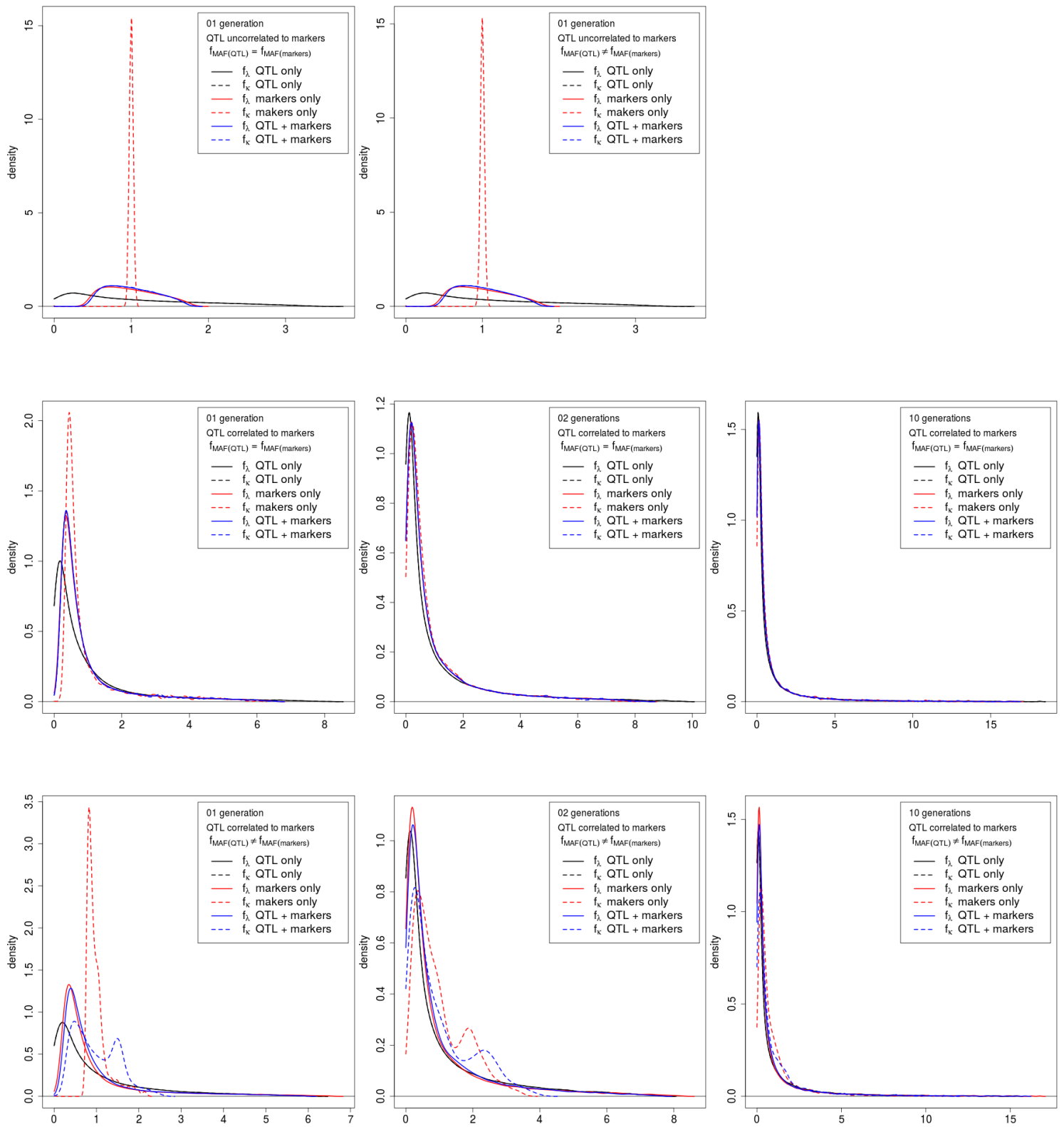

Supplement: Supplementary file 1 — Additional file 1. Distribution of $\lambda_{{\rm i}}$λi and $\kappa_{{\rm i}}$κi for the eight scenarios, when considering 3000 QTL and 2000 individuals. Distributions are presented for ${\mathbf{G}}$G assuming the true model (QTL only) and assuming both genomic models evaluated (QTL plus markers and markers only). [file 12711_2018_411_MOESM1_ESM.pdf]

Supplementary Figure 2

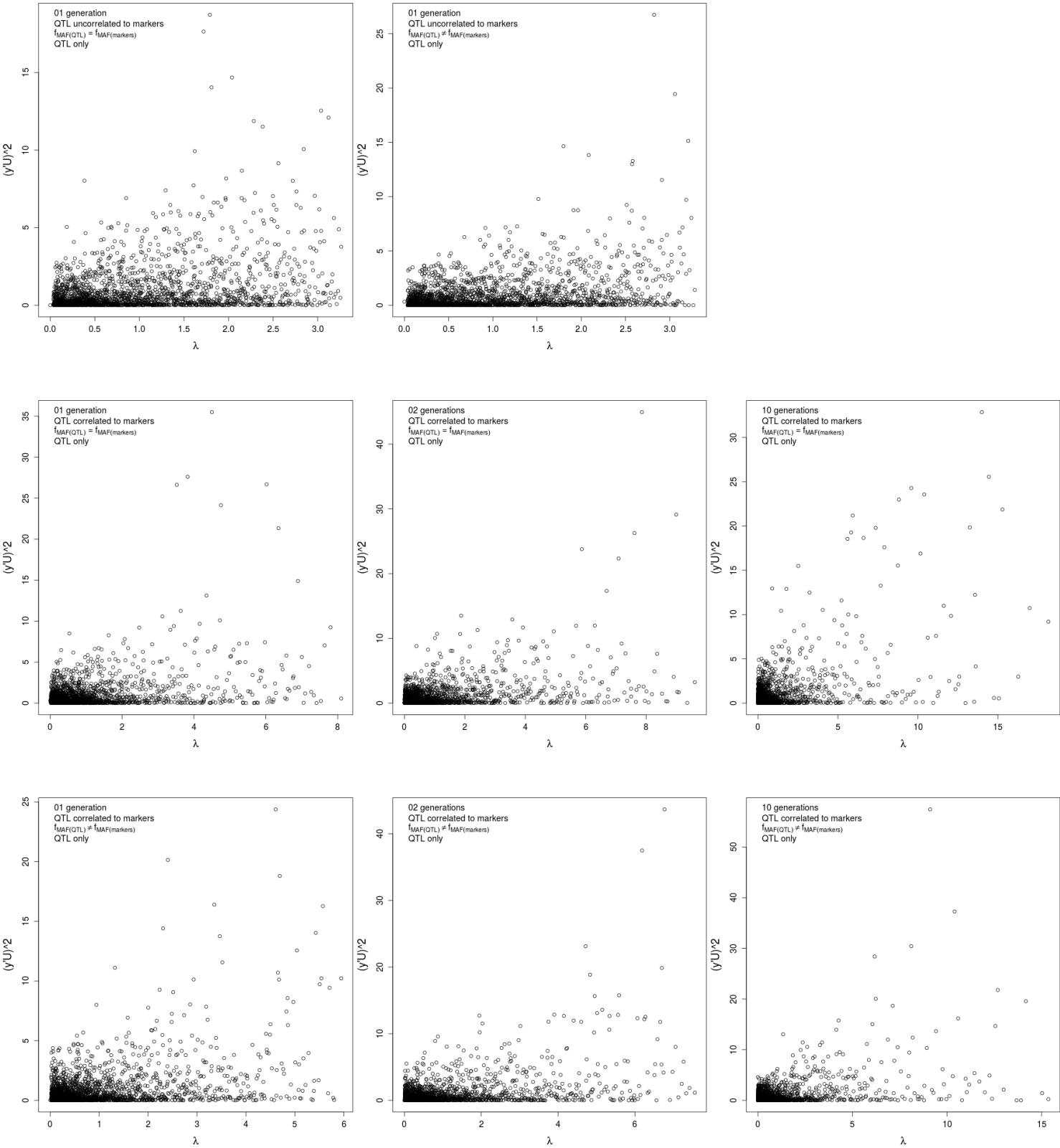

Supplement: Supplementary file 2 — Additional file 2. Scatterplot of $\lambda_{{\rm i}}$λi versus $({\mathbf{y}}\prime{\mathbf{U}}_{{\rm i}})^{2}$(y′Ui)2 for one replicate on all the eight scenarios, when considering 3000 QTL and 2000 individuals. Distributions are presented for ${\mathbf{G}}$G assuming the true model (QTL only). [file 12711_2018_411_MOESM2_ESM.pdf]

Supplementary Figure 3

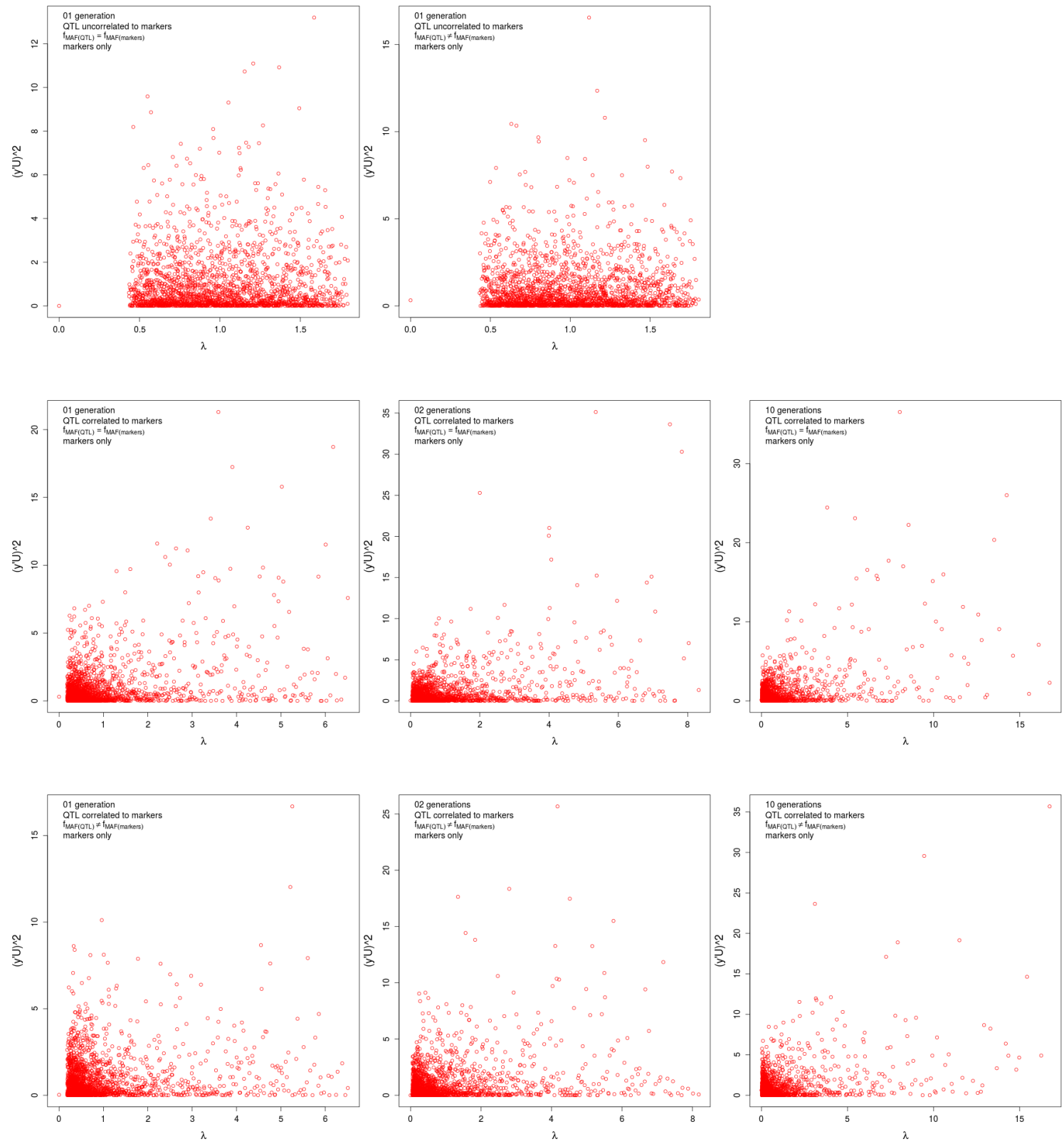

Supplement: Supplementary file 3 — Additional file 3. Scatterplot of $\lambda_{{\rm i}}$λi versus $(\mathbf{y}\prime \mathbf{U}_{{\rm i}})^{2}$(y′Ui)2 for one replicate on all the eight scenarios, when considering 3000 QTL and 2000 individuals. Distributions are presented for ${\mathbf{G}}$G assuming the genomic model with markers only. [file 12711_2018_411_MOESM3_ESM.pdf]

Supplementary Figure 4

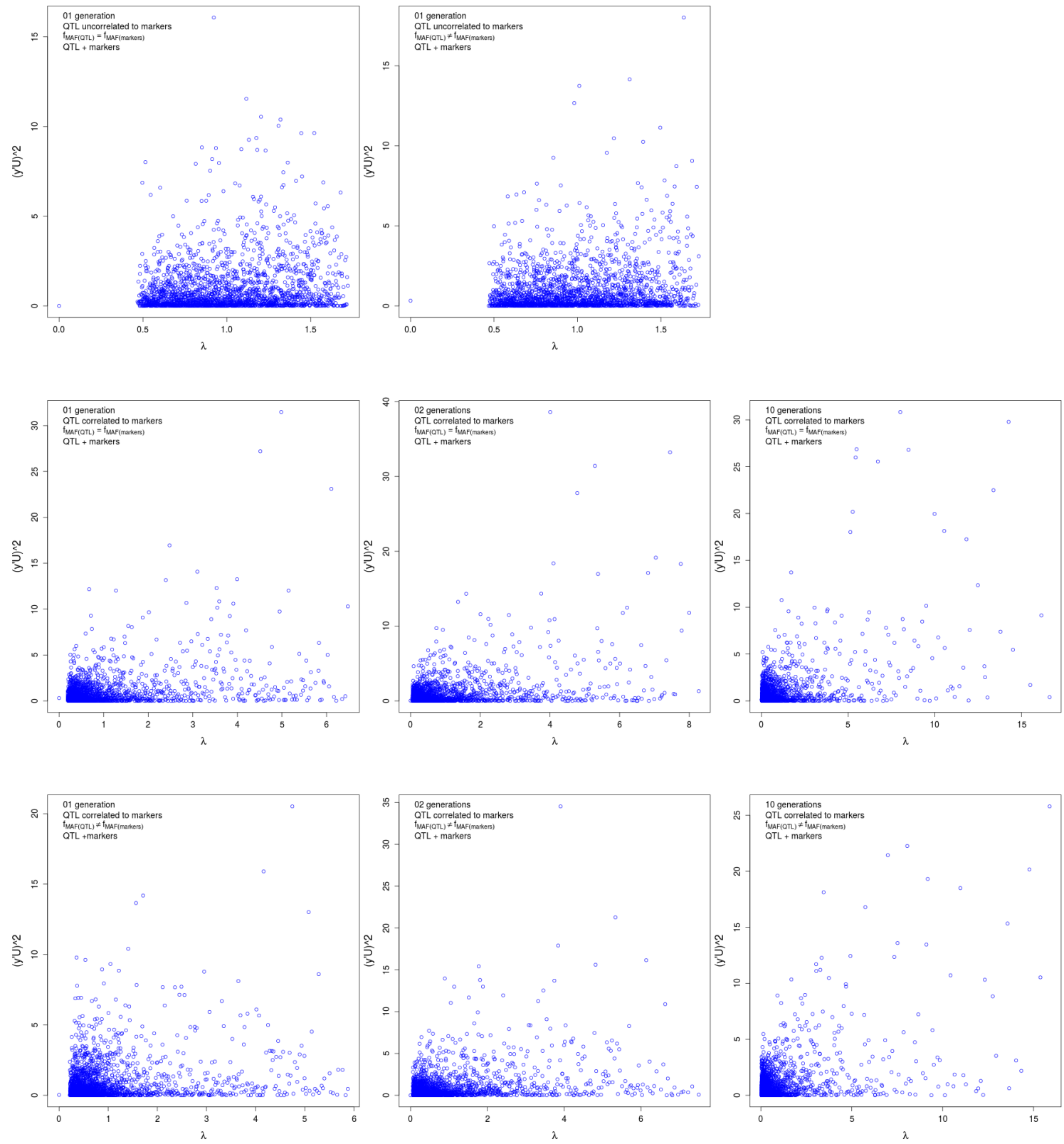

Supplement: Supplementary file 4 — Additional file 4. Scatterplot of $\lambda_{{\rm i}}$λi versus $(\mathbf{y}\prime \mathbf{U}_{{\rm i}})^{2}$(y′Ui)2 for one replicate on all the eight scenarios, when considering 3000 QTL and 2000 individuals. Distributions are presented for ${\mathbf{G}}$G assuming the genomic model with QTL plus markers. [file 12711_2018_411_MOESM4_ESM.pdf]

Supplementary Figure 5

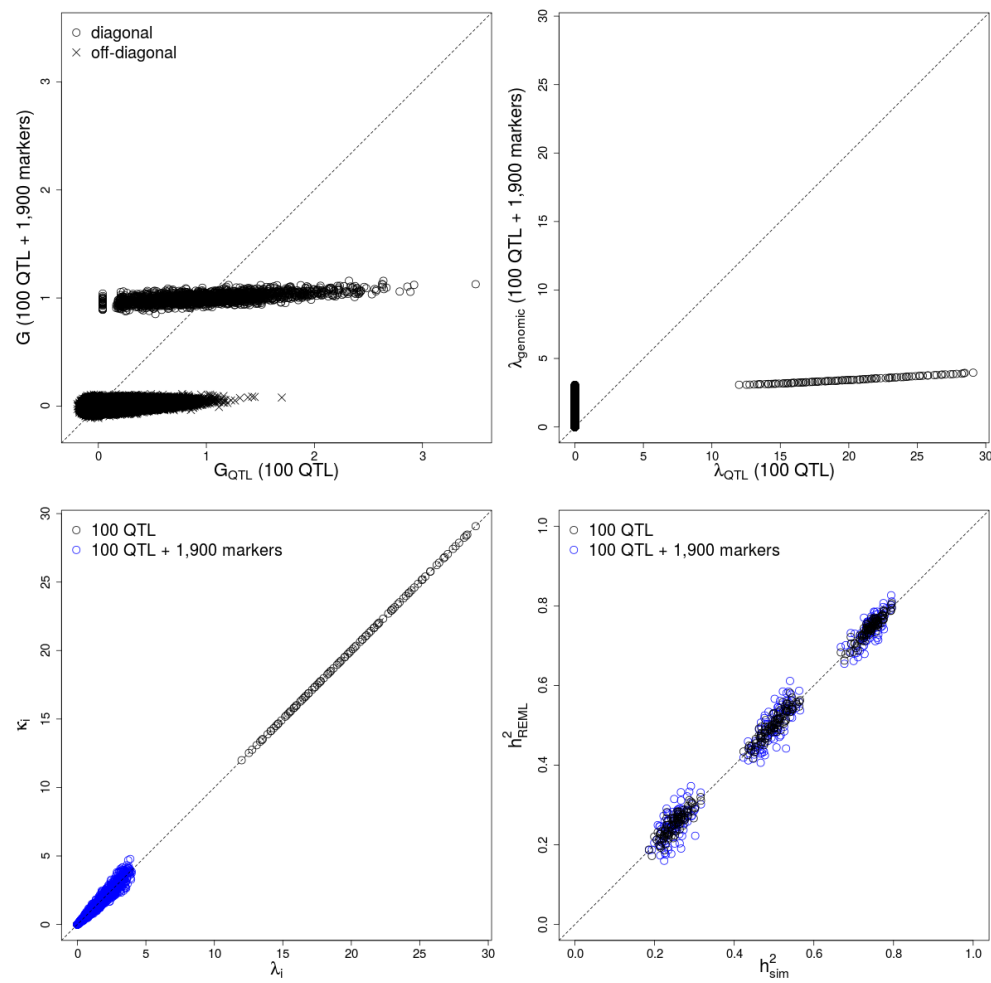

Supplement: Supplementary file 5 — Additional file 5. Comparison of ${\mathbf{G}}$G to ${\mathbf{G}}_{{\rm Q}}$GQ for 2000 completely unrelated individuals by: (1) direct comparison of values, (2) comparison of eigen-values ($\lambda_{{\rm i}}$λi), and (3) comparison of $\lambda_{{\rm i}}$λi to $\kappa_{{\rm i}}$κi, and comparison of $\hat{\hbox{h}}^{2}_{{\rm REML}}$h^REML2 to [file 12711_2018_411_MOESM5_ESM.pdf]

Supplementary Figure 6

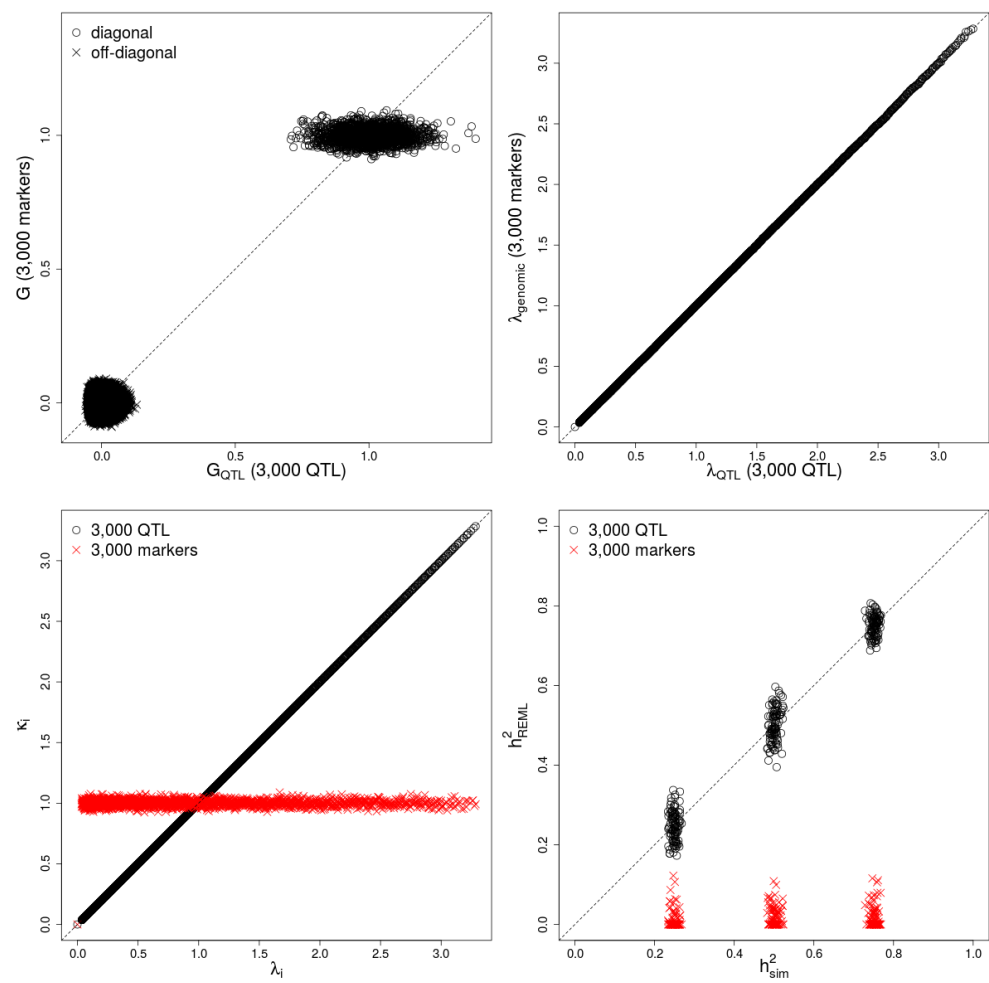

Supplement: Supplementary file 6 — Additional file 6. Comparison of ${\mathbf{G}}$G to ${\mathbf{G}}_{{\rm Q}}$GQ for 2000 completely unrelated individuals by: (1) direct comparison of values, (2) comparison of eigen-values ($\lambda_{{\rm i}}$λi), and (3) comparison of $\lambda_{{\rm i}}$λi to $\kappa_{{\rm i}}$κi, and comparison of $\hat{\hbox{h}}^{2}_{{\rm REML}}$h^REML2 to [file 12711_2018_411_MOESM6_ESM.pdf]

Supplementary Figure 7

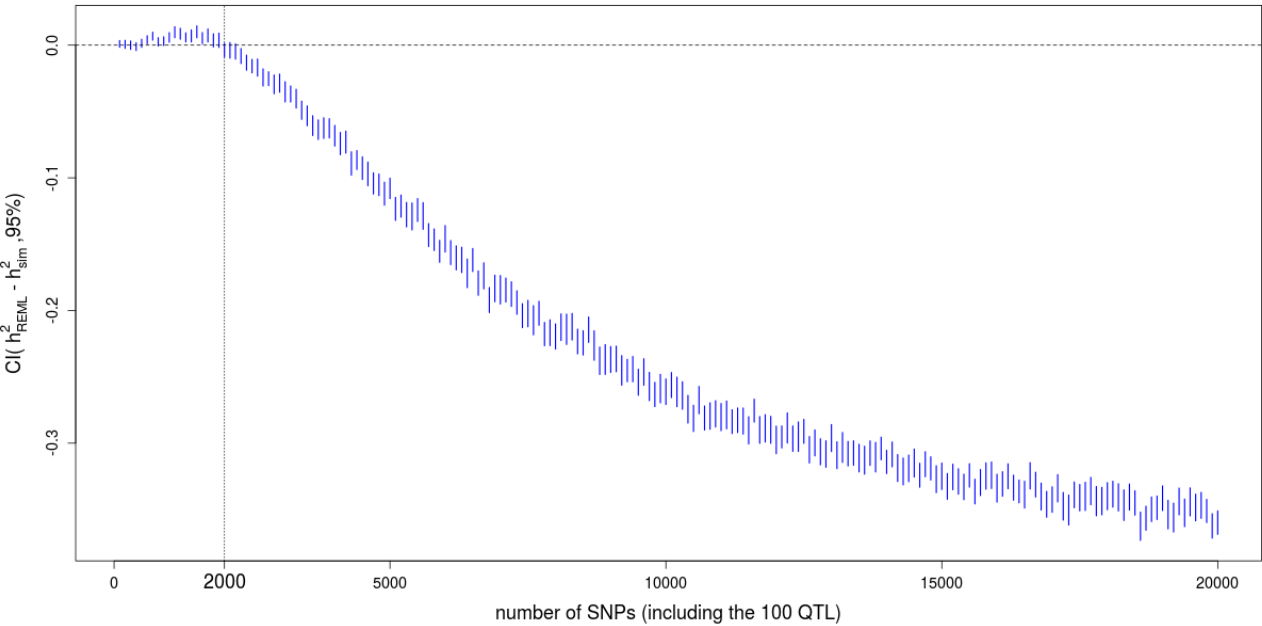

Supplement: Supplementary file 7 — Additional file 7. 95\% confidence intervals, based on 100 replicates, for the absolute bias of $\hat{\hbox{h}}^{2}_{{\rm REML}}$h^REML2 to $\hbox{h}^{2}$h2. Genomic data consisted of QTL plus markers for 2000 completely unrelated individuals. The number of markers in the SNP data varied from 0 to 19,900, and 100 QTL were used to simulate the phenotypes with $\hbox{h}^{2}$h2 parameter 0.6. QTL and markers were in LD, such that $\hbox{f}_{{\rm MAF}_{_{{\rm QTL}}}} \neq \hbox{f}_{{\rm MAF}_{_{{\rm markers}}}}$fMAFQTL≠fMAFmarkers. [file 12711_2018_411_MOESM7_ESM.pdf]

Supplementary Figure 8

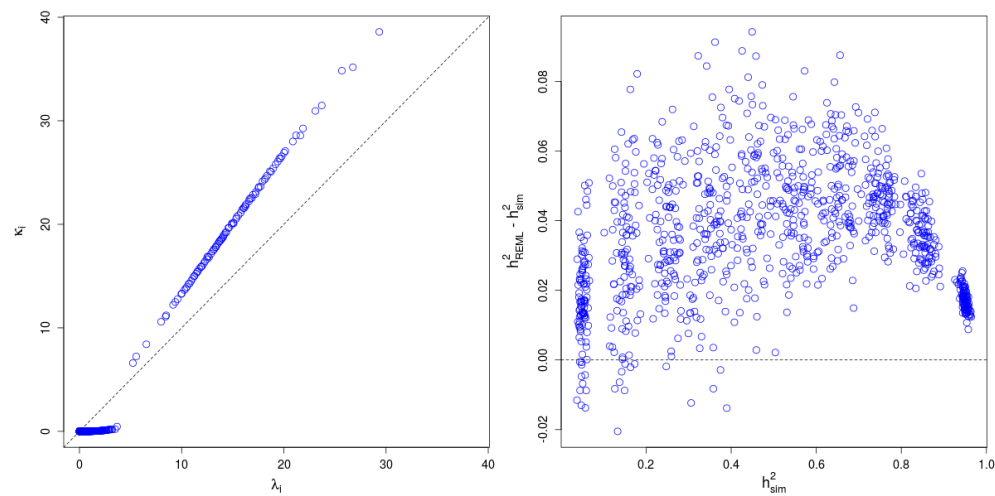

Supplement: Supplementary file 8 — Additional file 8. Relationship between $\lambda_{{\rm i}}$λi and $\kappa_{{\rm i}}$κi, and absolute bias of $\hat{\hbox{h}}^{2}_{{\rm REML}}$h^REML2 to $\hbox{h}^{2}$h2. Genomic data consisted of QTL plus markers for 2000 completely unrelated individuals. 100 QTL were used to simulate the phenotypes with $\hbox{h}^{2}$h2 parameter 0.05,0.15,…,0.95. QTL and markers were in LD, such that $\hbox{f}_{{\rm MAF}_{_{{\rm QTL}}}} \neq \hbox{f}_{{\rm MAF}_{_{{\rm markers}}}}$fMAFQTL≠fMAFmarkers. Markers were weighted according to their LD [file 12711_2018_411_MOESM8_ESM.pdf]
